# CBX7 silencing promoted liver regeneration by interacting with BMI1 and activating the Nrf2/ARE signaling pathway

**DOI:** 10.1038/s41598-024-58248-8

**Published:** 2024-05-14

**Authors:** Zhimin Dou, Fei Lu, Jinjing Hu, Bin Li, Xun Li

**Affiliations:** 1 The First School of Clinical Medicine, Lanzhou University, No. 199, Donggang West Road, Chengguan District, Lanzhou, 730000 Gansu China; 2 Department of Critical Care Medicine, The First Hospital of Lanzhou University, Lanzhou, 730000 China; 3 Key Laboratory of Biotherapy and Regenerative Medicine of Gansu Province, No. 1 Donggang West Road, Chengguan District, Lanzhou, 730000 Gansu China; 4 Department of General Surgery, The First Hospital of Lanzhou University, No. 1 Donggang West Road, Chengguan District, Lanzhou, 730000 Gansu China

**Keywords:** CBX7, BMI1, Liver regeneration, Cell biology, Molecular biology

## Abstract

Multiple studies have shown knockdown of chromobox 7 (CBX7) promotes the regenerative capacity of various cells or tissues. We examined the effect of CBX7 on hepatocyte proliferation and liver regeneration after 2/3 hepatectomy in a mouse model. For in vitro experiments, NCTC 1469 and BNL CL.2 hepatocytes were co-transfected with siRNA-CBX7-1 (si-CBX7-1), siRNA-CBX7-2 (si-CBX7-2), pcDNA-CBX7, si-BMI1-1, si-BMI1-2, pcDNA-BMI1, or their negative control. For in vivo experiments, mice were injected intraperitoneally with lentivirus-packaged shRNA and shRNA CBX7 before hepatectomy. Our results showed that CBX7 was rapidly induced in the early stage of liver regeneration. CBX7 regulated hepatocyte proliferation, cell cycle, and apoptosis of NCTC 1469 and BNL CL.2 hepatocytes. CBX7 interacted with BMI1 and inhibited BMI1 expression in hepatocytes. Silencing BMI1 aggregated the inhibitory effect of CBX7 overexpression on hepatocyte viability and the promotion of apoptosis. Furthermore, silencing BMI1 enhanced the regulatory effect of CBX7 on Nrf2/ARE signaling in HGF-induced hepatocytes. In vivo, CBX7 silencing enhanced liver/body weight ratio in PH mice. CBX7 silencing promoted the Ki67-positive cell count and decreased the Tunel-positive cell count after hepatectomy, and also increased the expression of nuclear Nrf2, HO-1, and NQO-1. Our results suggest that CBX7 silencing may increase survival following hepatectomy by promoting liver regeneration.

## Introduction

The mammalian liver has a strong regenerative ability due to the cell division of cholangiocytes and hepatocytes^1^. After removing 2/3 of the liver of the mouse the residual liver tissue can restore its original size in about 10 days and maintain the original function of the liver^2^. Liver regeneration is an important protection mechanism that guarantees the liver to maintain metabolic homeostasis^3^. However, when the liver suffers destructive damage or chronic liver damage, the liver's regenerative ability is significantly destroyed, and it is no longer possible to repair itself through regeneration, resulting in acute and chronic liver failure^4,5^. The prevalence of liver disease is increasing worldwide^6^. Acute and chronic liver insufficiency and liver failure caused by various liver diseases are major problems that clinicians need to solve. Thus, studying the molecular regulation mechanism of liver regeneration will provide potential biological therapeutic targets for the treatment of chronic hepatitis, liver fibrosis, liver failure, and other liver diseases, and has important scientific and clinical significance.

Polycomb Group (PcG) proteins, a family of master epigenetic regulators, regulate gene expression mainly through repression of gene transcription^7^. There are a variety of biological and pathological functions carried out by PCG proteins, including maintaining stemness, controlling the cell cycle, inactivating the X chromosome, and tumorigenesis^8–10^. The best-characterized protein complexes composed of PcG proteins are Polycomb Repressive Complexes 1 and 2 (PRC1 and PRC2)^11,12^. They act synergistically in chromatin recognition and transcription regulation at their common target genes. As one of the core components in PRC1, chromobox 7 (CBX7) protein facilitates the recruitment and stabilization of PRC1 to target chromatin^13^. Multiple studies have shown that knockout of CBX7 promotes the regenerative capacity of a variety of cells or tissues^14^. It has been shown that the knockdown of CBX7 has a positive effect on tooth extraction socket healing^15^. CBX7 was reported as a novel regulator of axon growth and regeneration. In particular, CBX7 deficiency increases the ability of dorsal root ganglion neurons (DRGs) to grow axons^16^. Similarly, CBX7 plays a critical role in the regenerative properties of adult pluripotent-like olfactory stem cells (APOSCs)^17^. The downregulation of hepatic CBX7 was confirmed in patients diagnosed with portal hypertension and exhibited a significant correlation with the hepatic venous pressure gradient^18^. In addition, CBX7 has been shown to inhibit liver cancer progression^19,20^. However, the effects of CBX7 on the regulation of liver regeneration have not been investigated.

B-lymphoma Mo-MLV insertion region (BMI1) is also an important member of the PcG protein family, which regulates cell differentiation and self-renewal. A prior study established that BMI1 contributed to the regeneration of the exocrine pancreas after cerulein-induced injury^21^. In addition, BMI1 (High)-positive cells played important roles in maintaining germ stem cells (GSCs) and in regenerating spermatogenic progenitors after injury^22^. In a mouse model of dystrophinopathy, BMI1 promoted skeletal muscle regeneration through metallothionein 1 (MT1)-mediated oxidative stress protection^23^. BMI1 was essential for efficient muscle regeneration after injury. BMI1^–/–^ mice leading to reduced postnatal muscle fiber size and impaired regeneration upon injury^24^.

The present study explored the regulatory effect and molecular mechanism of CBX7 on liver regeneration. Knockdown of CBX7 promotes liver regeneration, and its mechanism is closely related to the binding of BMI1 and the regulation of Nrf2-ARE pathway activity.

## Material and methods

### Animal model

Male C57/BL6 mice were purchased from Chengdu Dossy Experimental Animals CO., LTD. (Chengdu, Sichuan). Mice were fed and watered freely. Feeding conditions were 20–25 °C, relative humidity 50 ± 1%, and light/darkness for 12 h. The experimental protocol was reviewed and approved by the Experimental Animal Care and Ethics Committee of the First Hospital of Lanzhou University (LDYYLL2022-380). Animal studies were conducted by the Animal Research: Reporting of in Vivo Experiments (ARRIVE) guidelines, and all breeding and research on experimental animals strictly abide by the regulations on the administration of experimental animals at Lanzhou University.

In experiment 1, mice were randomly divided into two groups: sham group and partial hepatectomy (PH) group (24 per group). In experiment 2, using a random number generator, four groups of mice were divided: sham group, PH group, shRNA NC group, and shRNA CBX7 group (10 per group). Mice in the PH group were performed two-thirds PH using standard procedures^25^. The surgeries were performed between 9:00 AM and 12:00 PM. Mice in the shRNA NC group and shRNA CBX7 group were injected with lentivirus-packaged shRNA and shRNA CBX7 (50 μl 1 × 10^10 IU/ml) with the tail vein before modeling, respectively. the mice were euthanized with 70% (VDR/min) carbon dioxide (CO_2_) at 72 h after surgery. The livers were immediately excised and weighed for follow-up experiments.

### Cell culture and transfection

Mouse normal hepatocytes NCTC 1469 (CL-0407), were obtained from ProCell (Wuhan, China). The murine embryonic liver cell line BNL CL.2 was purchased from the Cell storeroom of the Chinese Academy of Sciences (Shanghai, China). After centrifugation at 1000xg for 10 min, cells were grown in DMEM complete medium (Gibco BRL, USA) containing 10% fetal bovine serum (FBS, Gibco BR L, USA) at 5% CO_2_ concentration at 37 °C.

BNL CL.2 and NCTC 1469 cells were transfected using Lipofectamine®2000 reagent (Invitrogen, Grand Island, NY), with different plasmids and RNA sequences transfecting different numbers of cells according to different experimental purposes. Liposomes, antibiotic-free OPTI-MEM medium (Gibco), and plasmids, or RNA interference sequences, or their respective negative controls were mixed and added to serum-free cell culture medium according to the Lipofectamine®2000 reagent instructions, and the medium containing the liposome complex was replaced with serum-containing complete medium after 4–6 h of incubation. The final concentration of siRNA and control sequence transfection was 100 nmol. After 48 h post-transfection, transfected cells were collected and treated with different concentrations of hepatocyte growth factor (HGF, 5, 10, and 20 ng/ml) for 24 h to induce hepatocyte proliferation in vitro.

### Cell proliferation assay

BNL CL.2 and NCTC 1469 cells were digested and seeded in 96-well plates (1.0 × 10^9^ cells/well). 100μL of DMEM medium containing 10% FBS and 10 μL cell counting kit-8 (CCK-8) solution were added to each well, and incubation was continued for 0.5–2 h in a cell incubator.

Then the absorbance was measured at 450 nm.

### RT-qPCR assay

The mRNA expression of CBX7 and BMI1 was evaluated using RT-qPCR. For gene analysis, equal amounts of cDNA were added to a reaction mixture containing gene-specific forward and reverse primers deoxynucleotide Taq DNA polymerase and SYBR (Bio-Rad, Her cules, CA) in a reaction mixture. Quantification of cDNA was based on monitoring increased SYBR fluorescence during exponential phase amplification in an RT-qPCR Machine (Bio-Rad, Hercules, CA), and determination of the PCR cycle number at which the amplified product exceeded a defined threshold.

### Western blot analysis

Liver tissues, NCTC 1469, and BNL CL.2 cell lyses solution was fabricated using RIPA buffer (Santa Cruz Biotechnology, Dallas, TX) to collect total proteins. Protein concentration was determined using the bicinchoninic acid (BCA) protein assay kit (Pierce, Rockford, IL). Thirty micrograms of total cellular protein were subjected to SDS-PAGE, followed by electrophoretic transfer to nitrocellulose. Filters were probed with a primary antibody followed by an HRP-conjugated anti-rabbit-IgG secondary antibody and then developed using the ECL system (Amersham, Piscataway, NJ). Densitometric analysis was performed with Scion Image analysis software v 4.02 (http://www.scioncorp.com/, Scion Corporation, Frederick, MD). The corresponding protein primary antibodies are present in Table 1.

**Table 1:** Antibodies in western blot.

Primary antibody	Commercial source	Catalog number	Species	Antibody type	Working concentration
CBX7	Abcam	ab21873	Rabbit	Polyclonal	1:1000
BMI1	Abcam	ab38295	Rabbit	Polyclonal	1:1000
CyclinD1	Abcam	ab16663	Rabbit	Monoclonal	1:50
CyclinE	Abclonal	A12000	Rabbit	Polyclonal	1:500
HO-1	Abcam	ab21873	Rabbit	Polyclonal	1:2000
NQO-1	Abclonal	A19586	Rabbit	Polyclonal	1:500
LaminB	Abcam	ab16048	Rabbit	Polyclonal	1:1000
β-actin	Abcam	ab8227	Rabbit	Polyclonal	1:1000

### Cell apoptosis and cell cycle analysis

Cells were washed with PBS (Invitrogen, Carlsbad, CA, USA) and diluted to 1.0 × 10^6^ cells/mL. A 150 μl buffer solution was then used to suspend the cells. An additional staining step was performed at 4 °C with 10 μg/ml Annexin V-FITC and 5 μl PI for 20 min in darkness. BD FACSCelestaTM Flow Cytometer (Becton, Dickinson, and Company) was used to analyze apoptotic cells. The cell cycle of NCTC1469 and BNL CL.2 cells was detected by BD FACSCalibur Flow Cytometry System using propidium iodide (Beyotime, Shanghai, China; C1052).

### Blood biochemical index detection

Venous blood was collected 12 h after PH, and the levels of alanine aminotransferase (ALT, #C009-2–1), aspartate aminotransferase (AST, #C010-2–1), albumin (ALB, #A028-2–1) and hepatocyte growth factor (HGF, #H181) were detected by biochemical kits (Nanjing Jiancheng Bioengineering Institute, Nanjing, China).

### Hematoxylin–eosin (H&E) stain

The hepatic lobules were fixed in a 4% paraformaldehyde solution overnight. Subsequently, the samples were processed and embedded in paraffin. The tissue sections, measuring 5 μm, were subjected to dewaxing with toluene, dehydration with ethanol, and washing with distilled water. The sections were then stained with hematoxylin, followed by rinsing and separation using an alcohol solution containing 1% hydrochloric acid. Eosin staining was applied, followed by a 10-min immersion in distilled water. The sections were dehydrated with xylene and sealed with a neutral glue. The resulting samples were examined using a digital trinocular camera microscope at magnifications of 400×.

### Immunohistochemistry (IHC) stain

The streptavidin-peroxidase (SP) method was performed in strict accordance with the kit instructions. 4 μm thick liver tissue sections were routinely dewaxed and hydrated with gradient ethanol. The samples were treated with antigen repair solution at 95–99 ℃ for 40 min and cooled at room temperature for 20 min. After washing 3 times, Ki-67 primary antibody (No. ab15580, Abcam, Cambridge, MA, USA; 1:100) or CK19 primary antibody (No. bs-15590R, Bioss, Beijing, China; 1:100) was added and incubated overnight at 4 ℃. Then the EnVision detection and color development kit was used for DAB color development, hematoxylin re-staining, gradient ethanol dehydration, xylene transparency, and then treacle sealing for observation. IHC images were evaluated microscopically (BA400Digital, Motic Instruments, Inc., Baltimore, MD, USA).

### Tunel stain

The paraffin section of liver tissues was dewaxed with different concentrations (100%, 95%, 80%, and 70%) of ethanol. The sections were then exposed to sodium citrate solution for antigenic repair. Deparaffinized brain sections were permeabilized with 0.1% Triton X-100 (ST795, Beyotime, Shanghai, China) for 8 min and were incubated with the Tunel reaction mix at 37 °C for 60 min. The sections were re-incubated with 4’,6’-diamidino-2-phenylindole (DAPI, Vector Laboratories, Burlingame, CA, USA) before the visualization of the sections with an optical microscope. Green Tunel dots were identified by the BX53 fluorescence microscope (Olympus, Tokyo, Japan).

### Co-immunoprecipitation (Co-IP)

CBX7 and BMI1 interactions were analyzed by Co-IP experiments. Briefly, we overexpressed His-tagged CBX7 or HA-tagged BMI1 in NCTC 1469 cells for 24 h. Next, NCTC 1469 cells were lysed in an IP buffer for 15 min. The lysate was centrifuged at 17,000 × g for 10 min at 4 °C. 2.5 mg of precleared lysate was then incubated overnight with 6 μg of anti-BMI1 or anti-CBX7 at 4 °C with gentle rotation. A 50% slurry of protein G-Sepharose beads was used to bind the immune complexes. SDS-PAGE and immunoblotting were used to separate the samples.

### Statistical analysis

The results of the experiments were statistically analyzed using SPSS 20.0 software (http://www.spss.com, SPSS, Chicago, IL, USA). The experimental results were expressed using the mean ± standard deviation (SD). One-way analysis of variance (ANOVA) was used for statistical analysis. A difference of p < 0.05 was defined as significant.

## Results

### CBX7 regulated hepatocyte proliferation, cell cycle, and apoptosis

**Figure 1:**
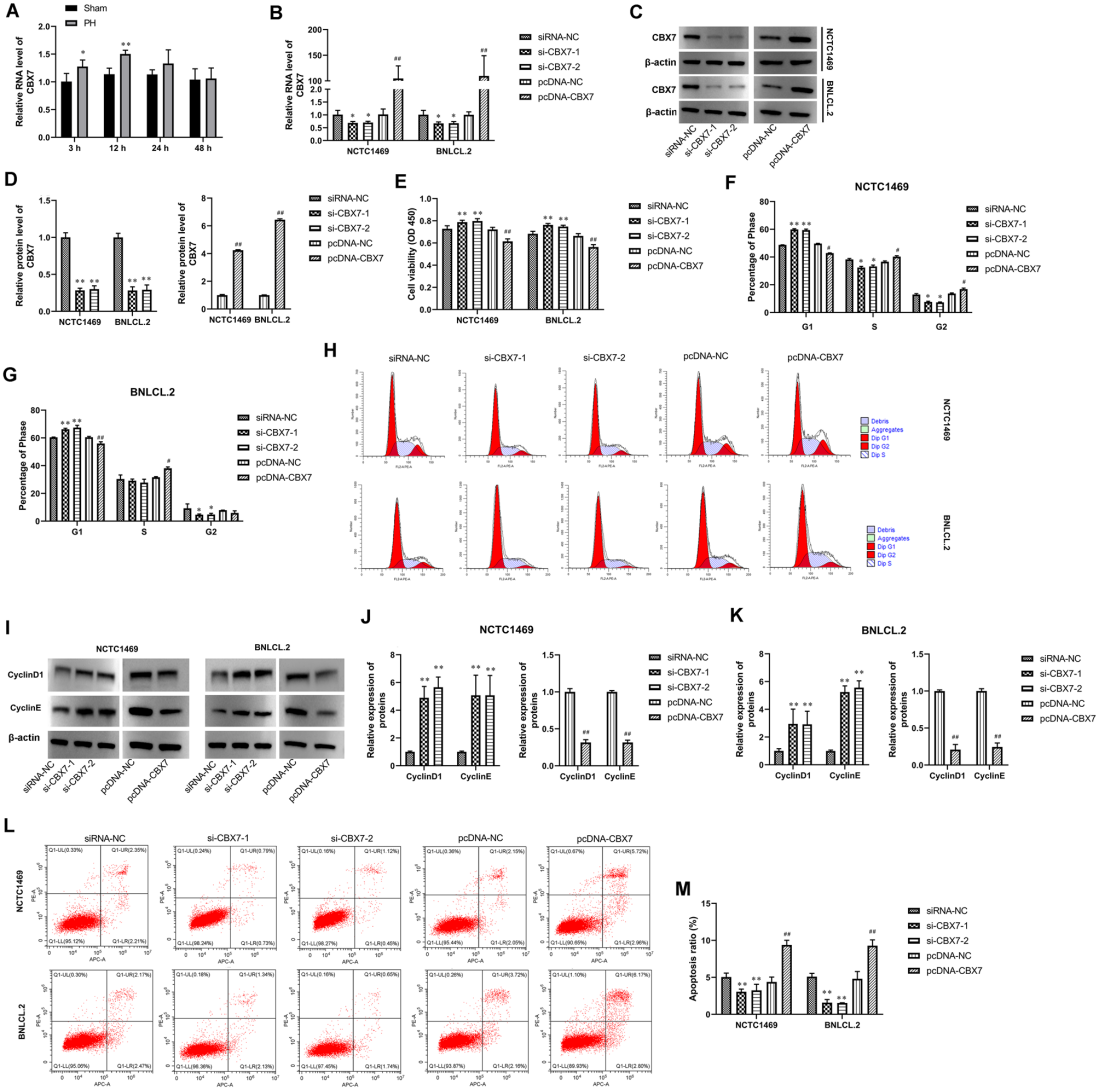
CBX7 regulated hepatocyte proliferation, cell cycle, and apoptosis. (**A**) CBX7 expression in liver tissues of PH mice at different time points was tested by RT-qPCR. (**B**) The RNA expression of CBX7 in NCTC 1469 and BNL CL.2 cells was assayed by RT-qPCR. (**C**,**D**) The protein expression of CBX7 in NCTC 1469 and BNL CL.2 cells was determined by Western Blot analysis. β-actin was a loading control. (**E**) The proliferation of NCTC 1469 and BNL CL.2 cells was evaluated by CCK-8 assay. (**F**–**H**) Cell cycle of NCTC 1469 and BNL CL.2 were observed by flow cytometry. (**I**–**K**) Western blot was used to assay CyclinD1 and CyclinE expression in NCTC 1469 and BNL CL.2 cells. (**L**,**M**) Cell apoptosis was tested by flow cytometry. Values are means ± SD, **P < 0.01 vs normal tissue. *P < 0.05, **P < 0.01 vs siRNA-NC; ^*#*^*P* < 0.05, ^*##*^*P* < 0.01 vs pcDNA-NC.

To investigate CBX7 expression in liver tissues of PH mice, liver tissue samples were collected at different time points after PH. The expression of CBX7 in liver tissues was determined using RT-qPCR. The expression of CBX7 was significantly higher than that in the sham group at 3 h and 12 h after PH (Fig. 1A). At 48 h after PH, the expression of CBX7 decreased to the same level as that in the sham group (Fig. 1A).

Next, to explore the potential function of CBX7 in liver cell viability, si-CBX7-1, si-CBX7-2, pcDNA-CBX7, and their negative control were transfected into NCTC 1469 and BNL CL.2 cell lines. The RNA and protein expression of CBX7 was decreased and increased by si-CBX7-1, si-CBX7-2, and pcDNA-CBX7 transfection, respectively (Fig. 1B–D). CCK-8 assay indicated that CBX7 silencing caused an increase in cell proliferation of hepatocytes compared with the siRNA-NC group (Fig. 1E). Conversely, CBX7 overexpression showed the opposite trend (Fig. 1E). The transfection of CBX7-siRNA successfully promoted cell cycle arrest (Fig. 1F–H). Meanwhile, western blot analysis evidenced that CBX7 silencing significantly induced cyclin D1 and cyclin E expression in hepatocytes, and pcDNA-CBX7 transfection showed the opposite results (Fig. 1I–K). In addition, the apoptosis level of hepatocytes was inhibited by CBX7-siRNA and was promoted by pcDNA-CBX7 (Fig. 1L,M).

### CBX7 interacted with BMI1 and inhibited BMI1 expression in hepatocytes

**Figure 2:**
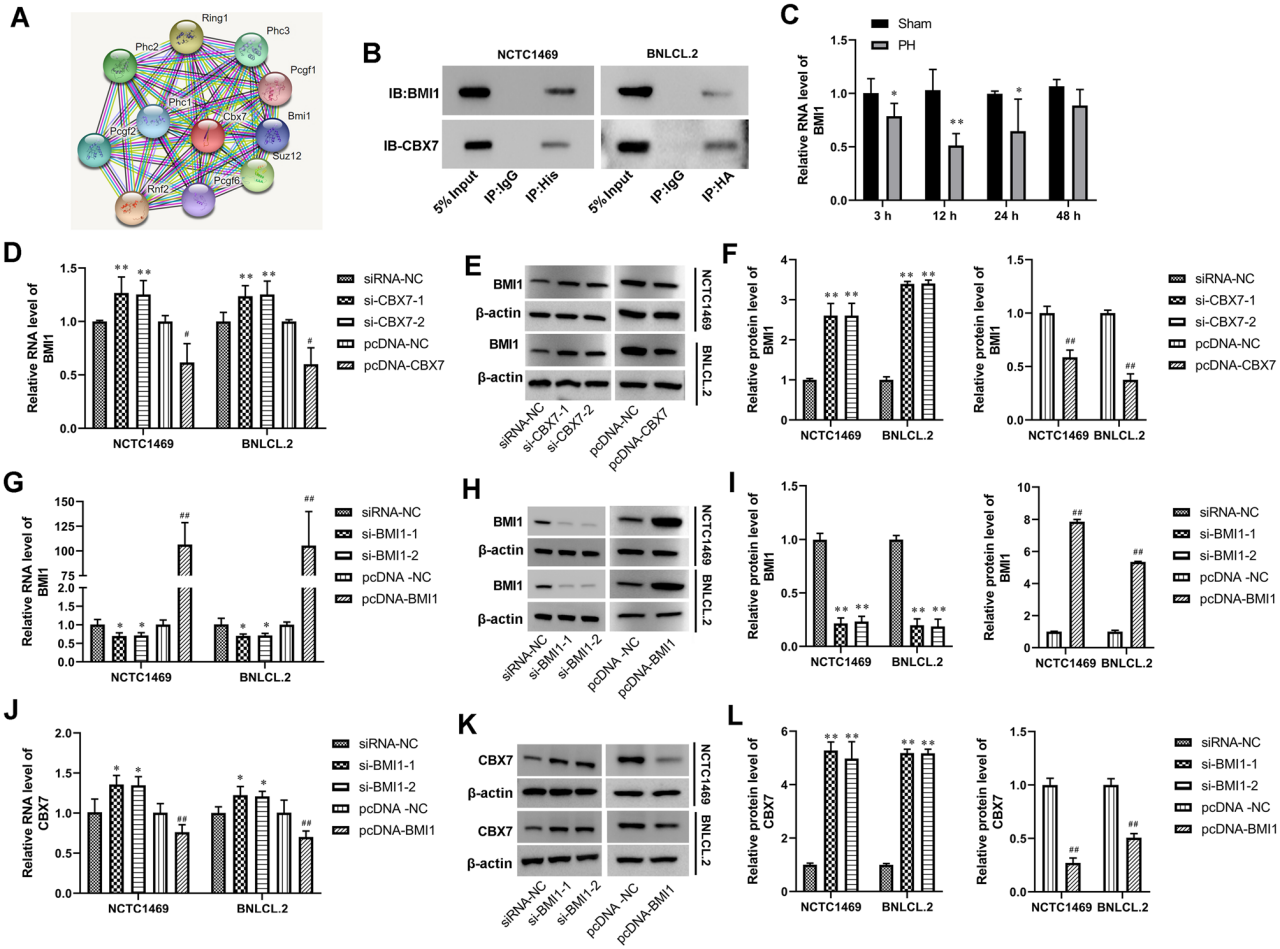
CBX7 interacted with BMI1 and inhibited BMI1 expression in hepatocytes. (**A**) proteins that interact with CBX7 are predicted by STRING (https://string-db.org/cgi/input.pl). (**B**) Co-IP assay was used to verify the mutual binding of CBX7 and BMI1. (**C**) BMI1 expression in liver tissues of PH mice at different time points was tested by RT-qPCR. BMI1 protein and RNA expression in NCTC 1469 and BNL CL.2 cells were analyzed by RT-qPCR (**D**,**G**) and western blot (**E**,**F**,**H**,**I**), respectively. CBX7 protein and RNA expression in NCTC 1469 and BNL CL.2 cells was detected by RT-qPCR (**J**) and Western blot (**K**,**L**), respectively. β-actin was a loading control. Values are means ± SD, **P < 0.01 vs normal tissue. *P < 0.05, **P < 0.01 vs siRNA-NC; ^*#*^*P* < 0.05, ^*##*^*P* < 0.01 vs pcDNA-NC.

As shown in Fig. 2A, A STRING prediction predicts proteins that interact with CBX7. The results suggested that CBX7 could interact with BMI1 (Fig. 2A). Co-IP further revealed an interaction of CBX7 with BMI1 (Fig. 2B). Moreover, The expression of BMI1 was significantly lower than that in the sham group at 3 h, 12 h, 24 h after PH (Fig. 2C). At 48 h after PH, the expression of BMI1 increased to the same level as that in the sham group (Fig. 2C). In NCTC 1469 and BNL CL.2 cells, the mRNA and protein expression of BMI1 was promoted by CBX7 silencing and was inhibited by CBX7 overexpression (Fig. 2D–F). Subsequently, si-BMI1 or pcDNA-BMI1 was transfected into NCTC 1469 and BNL CL.2 cells to efficiently silence and overexpress BMI1 (Fig. 2G–I). Our data demonstrated that BMI1 silencing induced CBX7 expression, whereas BMI1 overexpression reduced CBX7 expression in hepatocytes (Fig. 2J–L). Collectively, these results demonstrated that CBX7 could interact with BMI1 and negatively regulate the expression of BMI1.

### Silencing BMI1 aggregated the inhibitory effect of CBX7 overexpression on hepatocyte viability and the promotion of apoptosis

**Figure 3:**
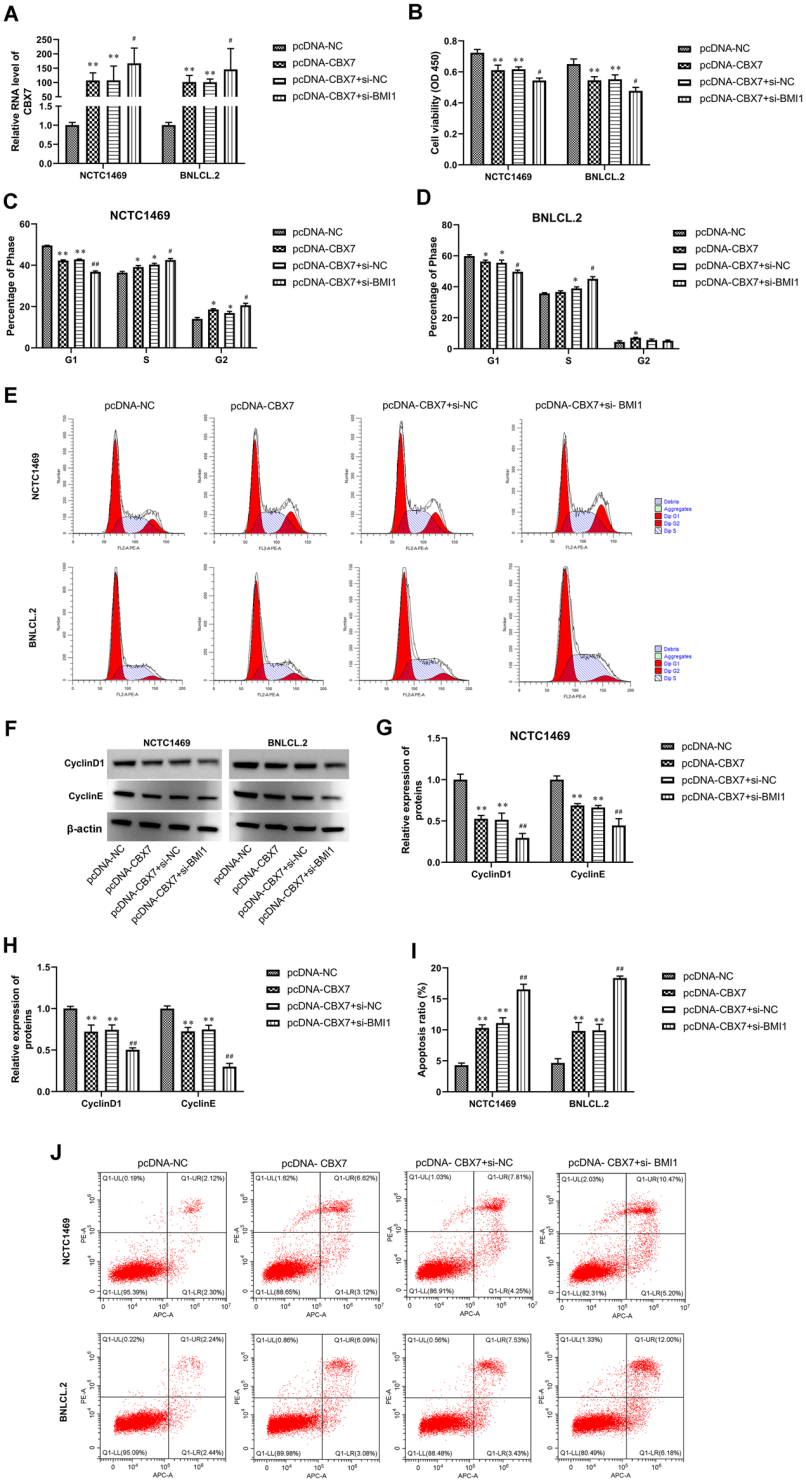
Silencing BMI1 aggregated the inhibitory effect of CBX7 overexpression on hepatocyte viability and the promotion of apoptosis. (**A**) CBX7 RNA expression in NCTC 1469 and BNL CL.2 cells was detected by RT-qPCR. (**B**) The proliferation of NCTC 1469 and BNL CL.2 cells was evaluated by CCK-8 assay. (**C**–**E**) Cell cycle of NCTC 1469 and BNL CL.2 were observed by flow cytometry. (**F**–**H**) Western blot was used to assay CyclinD1 and CyclinE expression in NCTC 1469 and BNL CL.2 cells. (**I**,**J**) Cell apoptosis was tested by flow cytometry. Values are means ± SD, *P < 0.05, **P < 0.01 vs pcDNA-NC; ^*#*^*P* < 0.05, ^*##*^*P* < 0.01 vs pcDNA-CBX7 + si-NC.

CBX7 expression was promoted by CBX7 overexpression, and further strengthened by BMI1 silencing co-transfection in hepatocytes (Fig. 3A). Furthermore, CBX7 overexpression inhibited cell proliferation of hepatocytes, which were further weakened by BMI1 silencing (Fig. 3B). Flow cytometry results showed that CBX7 overexpression suppressed cell cycle arrest of hepatocytes, which were further inhibited by BMI1 silencing (Fig. 3C–E). In addition, the decreased expression of CyclinD1 and CyclinE in pcDNA-CBX7-transfected NCTC 1469 and BNL CL.2 cell lines was further decreased by BMI1 silencing (Fig. 3F–H). Meanwhile, the apoptosis of hepatocytes was further eliminated by si-BMI1 co-transfection compared with the pcDNA- CBX7-transfected groups (Fig. 3I,J).

### Silencing BMI1 enhanced the regulatory effect of CBX7 on the Nrf2/ARE signaling pathway in HGF-induced hepatocytes

**Figure 4:**
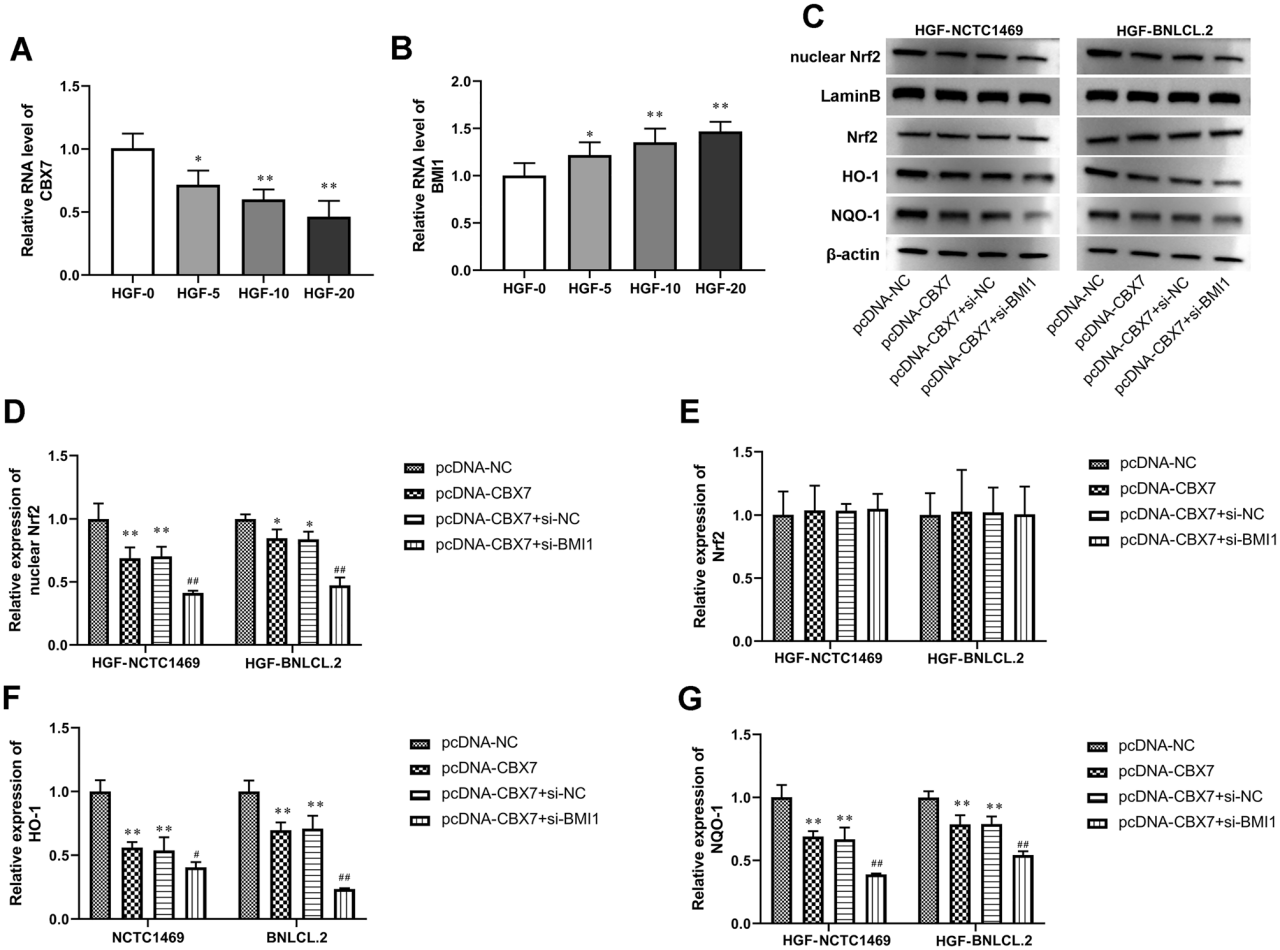
Silencing BMI1 enhanced the regulatory effect of CBX7 on the Nrf2/ARE signaling pathway in HGF-induced hepatocytes. (**A**,**B**) The RT-qPCR was used to assay the expression of CBX7 and BMI1 in NCTC 1469 cells induced by different concentrations of hepatocyte growth factor (HGF, 5, 10, and 20 ng/ml). (**C**–**G**) Nuclear Nrf2, Nrf2, HO-1, and NQO-1 expression in NCTC 1469 and BNL CL.2 cells was tested by Western blot. β-actin and lamin B was loading control. Values are means ± SD, *P < 0.05, **P < 0.01 vs HGF-0 or pcDNA-NC; ^*#*^*P* < 0.05, ^*##*^*P* < 0.01 vs pcDNA-CBX7 + si-NC.

HGF induces mitosis in hepatocytes by activating the tyrosine kinase receptor c-Met, which is the predominant mitogen in hepatocytes^26^. Thus, We used different doses of HGF (5, 10, and 20 ng/ml) to induce NCTC1469 cells and detected the changes in the expression of CBX7 and BMI1. As shown in Fig. 4A,B, HGF induction concentration-dependently decreased the expression level of NCTC 1469 cells, while it increased the expression level of BMI1 in a concentration-dependent manner. Mechanically, CBX7 overexpression suppressed the nuclear expression of Nrf2 and decreased the expression of downstream genes HO-1 and NQO-1 in HGF-induced hepatocytes, all of which were enhanced by silencing of BMI1 (Fig. 4C–G). These results suggest that CBX7 overexpression inhibited Nrf2/ARE signaling pathway activity during HGF-induced hepatocyte regeneration, which was further enhanced by BMI1 silencing.

### CBX7 silencing promoted liver regeneration in vivo

**Figure 5:**
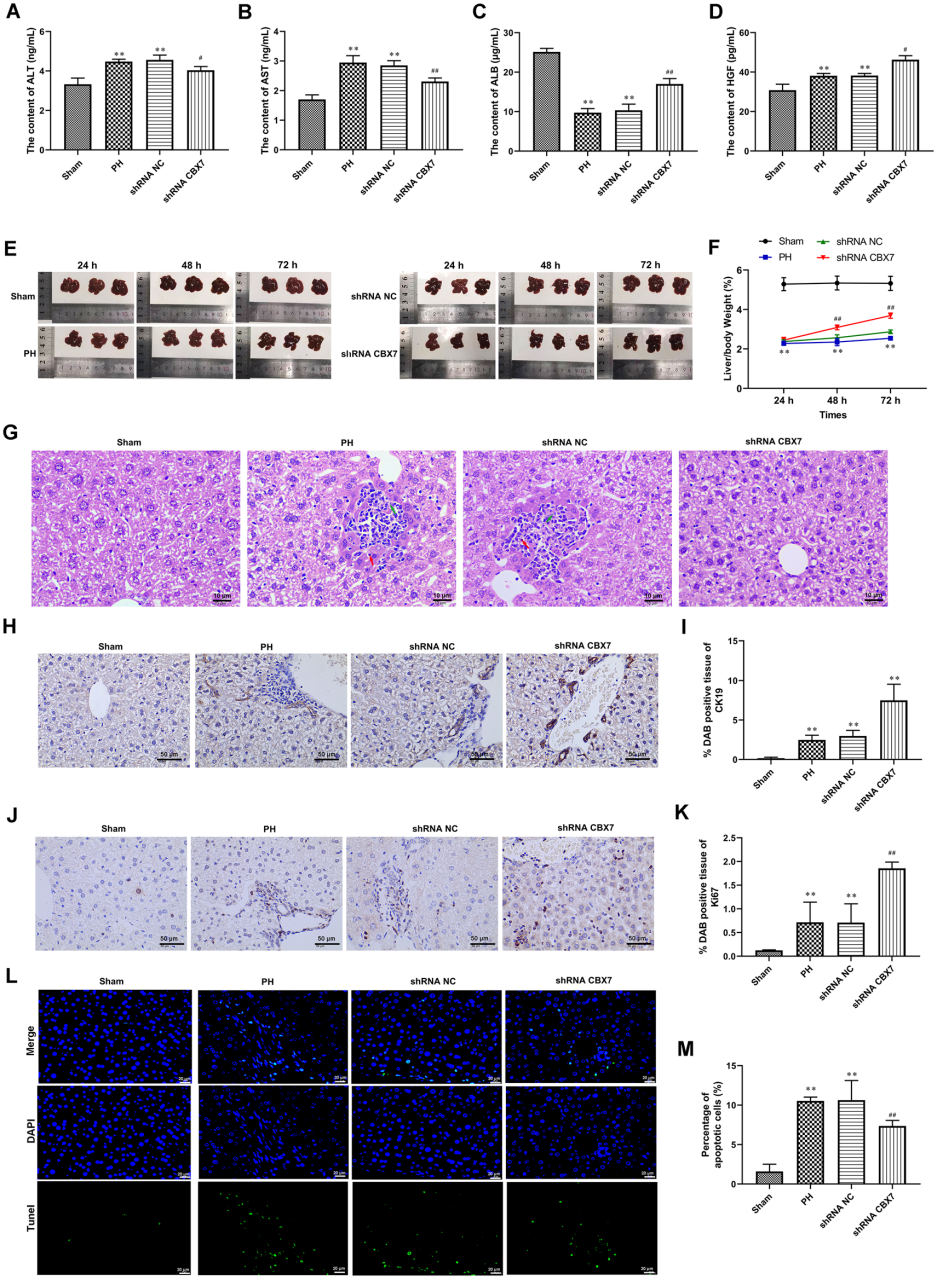
CBX7 silencing promoted liver regeneration in vivo. (**A**–**D**) The serum levels of alanine transaminase (ALT), aspartate transaminase (AST), total protein, albumin (ALB), and HGF were assayed automatic Biochemical Detector. (**E**) Liver regeneration grossing images. (**F**) Liver/body weight ratio. (**G**) H&E stain of liver tissues. Green arrows, hepatocyte degeneration and necrosis, red arrows, lymphocyte. Scale bar, 10 μm. (**J**,**K**) The expression of bile duct marker CK19 in liver tissues of mice was tested by IHC stain. Scale bar, 50 μm. (**J**,**K**) Ki67 expression in liver tissues of mice was tested by IHC stain. Scale bar, 50 μm. (**L**,**M**) Cell apoptosis of liver tissues was analyzed using a Tunel assay. Scale bar, 20 μm.

**Figure 6:**
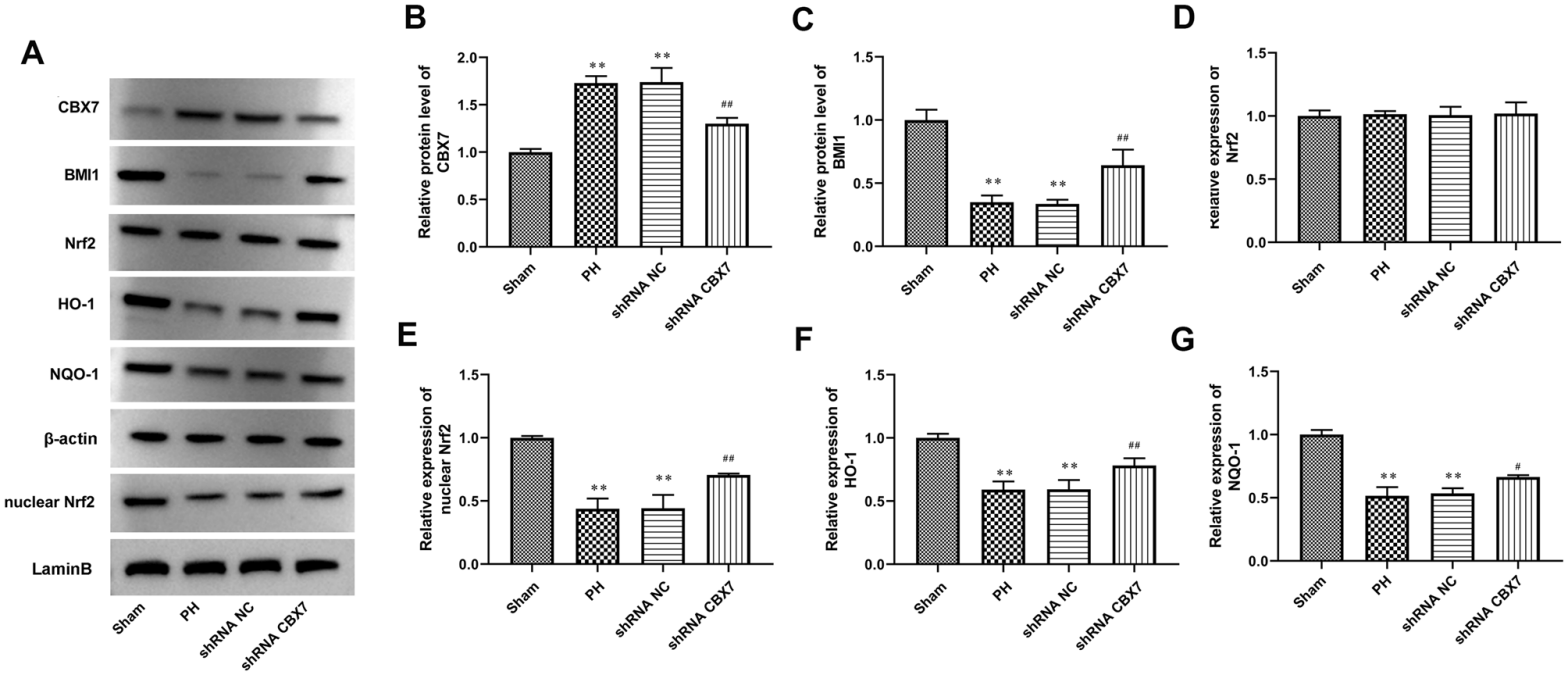
CBX7 silencing stimulated Nrf2/ARE signaling pathway activity in liver tissue. (**A**–**G**) CBX7, BMI1, nuclear Nrf2, Nrf2, HO-1, and NQO-1 expression in liver tissues was tested by Western blot. β-actin and lamin B were a loading control. Values are means ± SD, **P < 0.01 vs Sham; ^*#*^*P* < 0.05, ^*##*^*P* < 0.01 vs shRNA NC.

The liver function of mice was detected using an automatic biochemical detector. Compared with the sham group, the levels of ALT, AST, and HGF in the serum of mice after PH were significantly increased, while the levels of ALB and were significantly decreased (Fig. 5A–D). Notably, CBX7 silencing significantly decreased the levels of ALT, AST and increased the levels of ALB and HGF in the serum of mice after PH (Fig. 5A–D). As shown in Fig. 5E–F, the liver weight of mice in the PH group was decreased significantly after 24 h, 48 h and 72 h compared with the sham group. In addition, compared with the shRNA NC group, the liver/body weight ratio was increased significantly after CBX7 silencing (Fig. 5E–F). H&E staining suggested focal necrosis and inflammatory cell infiltration in the model group (Fig. 5G). However, CBX7 silencing decreased the necrotic cells and inflammatory infiltration (Fig. 5G). Then, the expression of the bile duct epithelial cell marker CK19 in liver tissue was detected by IHC stain (Fig. 5H,I). Compared with sham operation group, CK19 stain showed significant hyperplasia of bile duct in liver tissue of mice after PH (Fig. 5H,I). In addition, CBX7 silencing further promoted the CK19 positive percentage of bile duct epithelial cells (Fig. 5H,I). IHC results confirmed that the expression of the proliferation marker Ki67 was significantly induced in the liver tissue of mice after PH and was further enhanced by CBX7 silencing (Fig. 5J,K). The level of apoptosis in mouse liver tissue was significantly increased after PH and was counteracted by CBX7 silencing (Fig. 5L,M). Western blot analysis showed that the increased expression of CBX7 in liver tissue was significantly decreased by CBX7 silencing (Fig. 6A,B). Meanwhile, the decreased expression of BMI1 in liver tissue was significantly increased by CBX7 silencing (Fig. 5I,K). Mechanically, CBX7 silencing promoted Nrf2 nuclear translocation and enhanced the HO-1 and NQO-1 expression in the liver tissue of PH mice (Fig. 6A,C–G).

## Discussion

The mouse liver regeneration process is affected by circadian rhythm. Matsuo et al. found that after PHx in mice, mouse hepatocytes from G2 to S phase always occurred at the same time every day^27^. Weglarz et al. confirmed that DNA synthesis and replication in mice began to increase significantly at 32 h after PH and peaked at 40 h, while DNA synthesis in rats peaked 12–16 h earlier than in mice^28^. In the present study, CBX7 expression was increased at 3 h after PH peaked at 12 h, and returned to normal level at 48 h. In addition, the expression of BMI showed the opposite trend. To investigate whether CBX7 could directly regulate hepatocyte proliferation, we used NCTC 1469 and BNLCL.2 hepatocytes as in vitro models. The results showed that CBX7 overexpression inhibited hepatocyte proliferation and entered the cell cycle by negatively regulating BMI1 expression.

The process of liver regeneration is a complex pathophysiological process, which is mainly divided into the initial stage, the proliferation stage, and the termination stage. The proliferation of hepatocytes during liver regeneration requires the action of several growth factors, such as HGF and EGF. c-Met is a receptor for HGF, which was secreted by hepatic stromal cells next to hepatocytes. HGF bound to c-Met t on the surface of hepatocytes and activated ERK1/2, thereby promoting hepatocytes to enter the cell cycle. Cell cycle-related proteins are important players in liver regeneration and proliferation. Cyclin D1, mainly expressed in the early stage of DNA replication (G1 phase), was a key protein for cells to cross the G1/S checkpoint and was also the most important protein in regulating cells from the early stage of DNA replication to DNA replication (S phase). The synthesis of Cyclin E started in the mid-G1 phase, and the expression level was highest after the cells entered the S phase. Meanwhile, the degradation of Cyclin E also occurred in the S phase, while Cyclin E was not expressed in the G2 and M phases. Similar to Cyclin D1, Cyclin E is a key protein that assists cells to transition from the G1 phase to the S phase. The previous report documented that remifentanil increased the expression of cyclin D1 in the livers of PH rats^29^. In addition, prostaglandin E1 (PGE1) significantly increased the expression levels of Cyclin C and Cyclin D1 after hepatectomy^30^. In male Wistar rats, the absence of bile in the intestinal tract delayed liver regeneration associated with cyclin E-associated kinase inactivation at 18 h after 70% hepatectomy^31^. Oltipraz enhanced the expression of cyclin E and the activity of cyclin E-dependent kinase in PH rats^32^. In this study, we found that CBX7 overexpression inhibited cyclin D1 and cyclin E in NCTC 1469 and BNLCL.2 hepatocytes by negatively regulating the expression of BMI1. These data indicated that CBX7 overexpression blocked liver cell regeneration in vitro.

ALT and AST are enzymatic catalysts intricately involved in the intricate process of protein metabolism^33^. These enzymes assume a pivotal function in the intricate process of liver regeneration subsequent to PH, actively participating in the intricate synthesis and intricate repair of hepatic proteins^34,35^. ALB is a prominent hepatic protein that assumes a crucial role in various physiological processes^36^. It serves as an essential transport protein, contributing significantly to the maintenance of plasma osmolarity, nutrient transportation, and drug metabolism^36,37^. HGF is a pivotal protein that plays a crucial role in facilitating liver regeneration^38^. Its primary function involves stimulating the proliferation of hepatocytes, thereby aiding in the repair and restoration of liver function subsequent to surgical interventions^38,39^. Our data showed that the silencing of CBX7 resulted in a significant reduction in the levels of ALT, AST, while concurrently increasing the levels of ALB and HGF in the serum of mice after PH. The regenerative process following liver resection may trigger apoptotic pathways, resulting in the activation of apoptotic cells^40^. In addition, the surgical trauma itself can cause oxidative stress and inflammation, which may contribute to cell death and apoptosis^41,42^. The previous study showed that apoptotic liver cells were significantly increased in the liver resection group compared with the sham group^43^. Meanwhile, it was reported that the percentage of TUNEL-positive cells was already increased 3 days after PH in control animals, and was decreased again after 10 days^44^. Similarly, the present study indicated that revealed a noteworthy elevation in the apoptotic rate of mouse liver tissue after 72 h of PH, which was subsequently alleviated by the inhibition of CBX7.

The Keap1/Nrf2/ARE pathway is one of the most important endogenous antioxidant stress pathways known at present^45^. Nrf2 transcription factor is anchored in the cytoplasm through binding to Keapl and facilitates ubiquitination/proteolysis of Nrf2^45,46^. Inactivation of Keap1 leads to stabilization of Nrf2, which in turn translocates into the nuclei to activate cytoprotective target genes through binding to ARE^47^. Nrf2/HO-1 driven regulation of anti-oxidant and anti-inflammatory functions is important in cytoprotection^48,49^. Moreover, the report demonstrated the regulator role of Nrf-2 in the cellular cycle of the hepatocyte^50^. Keap1/Nrf2/ARE signaling attenuated hypoxia/reoxygenation injury in hepatocytes by inhibiting apoptosis and oxidative stress^51^. Particularly, previous reports revealed that Nrf2 impeded liver regeneration after PH^52,53^. Meanwhile, dynamic and coordinated regulation of Keap1/Nrf2/ARE and p53/p21 signaling pathways was associated with compensatory liver regeneration after acetaminophen (APAP) -induced acute liver injury^54^. In this study, the overexpression of CBX7 inhibited Nrf2 nuclear translocation and decreased HO-1 and NQO-1 expression, which were further aggravated by BMI1 silencing. These results suggest that CBX7 inhibited Nrf2/ARE signaling pathway activity after PH.

In conclusion, after PHx in mice, CBX7 was rapidly induced in the early stage of liver regeneration. CBX7 overexpression could significantly inhibit the proliferation and promote DNA damage of liver cells in vivo and in vitro. Mechanically, CBX7 decreased Nrf2/ARE signaling pathway activity by negatively regulating BMI1 expression. Taken together, our results highlight the importance of CBX7 knockdown during liver regeneration.

### Supplementary Information

Supplementary Information.

## Data Availability

The datasets used or analyzed during the current study are available from the corresponding author upon reasonable request.
