# Supplementary material for: CBX7 silencing promoted liver regeneration by interacting with BMI1 and activating the Nrf2/ARE signaling pathway
Source: Sci Rep. 2024 May 14;14:11008. doi: 10.1038/s41598-024-58248-8 (PMC11094083; doi:10.1038/s41598-024-58248-8)

**Figure 1C**

**NCTC1469: CBX7**


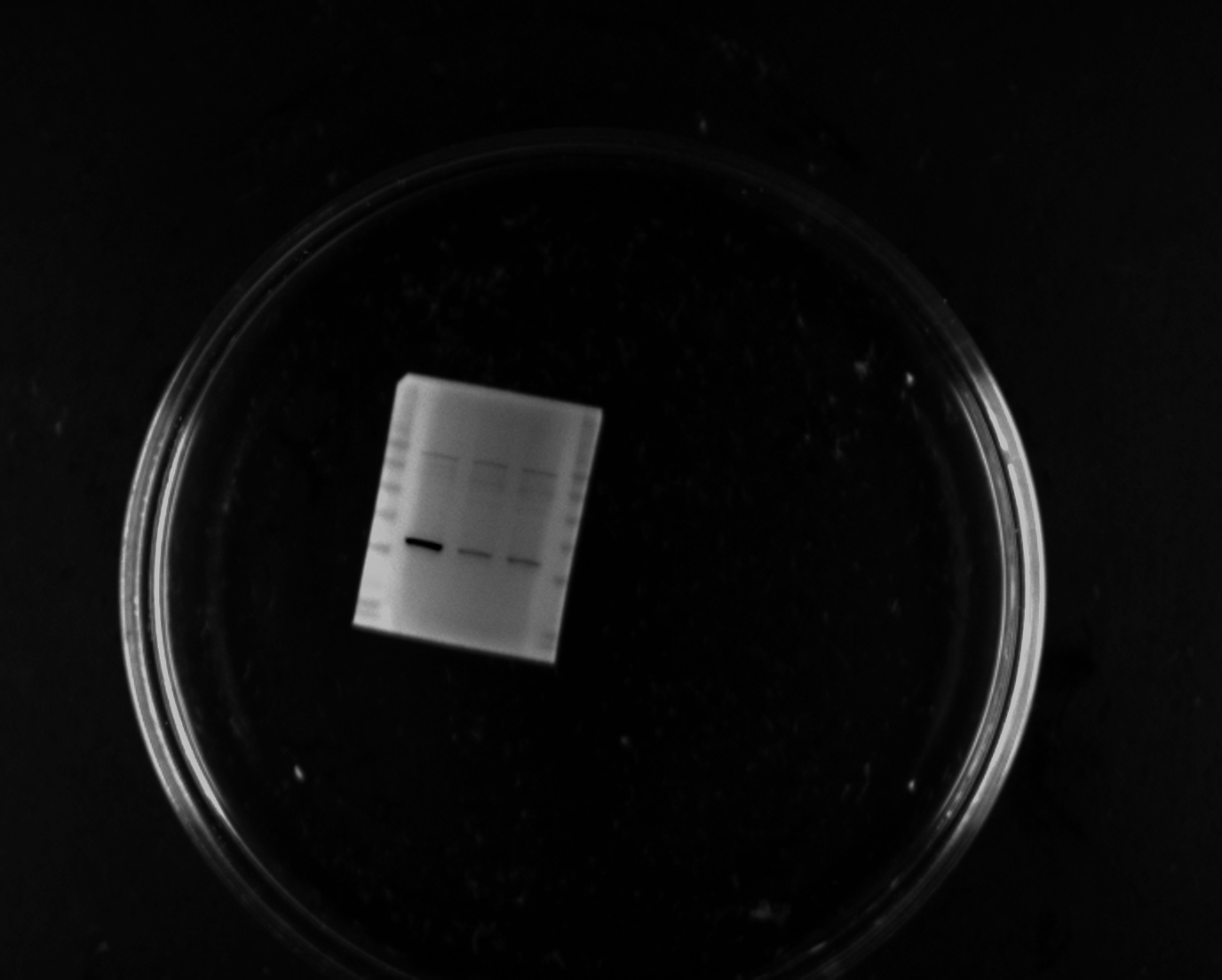

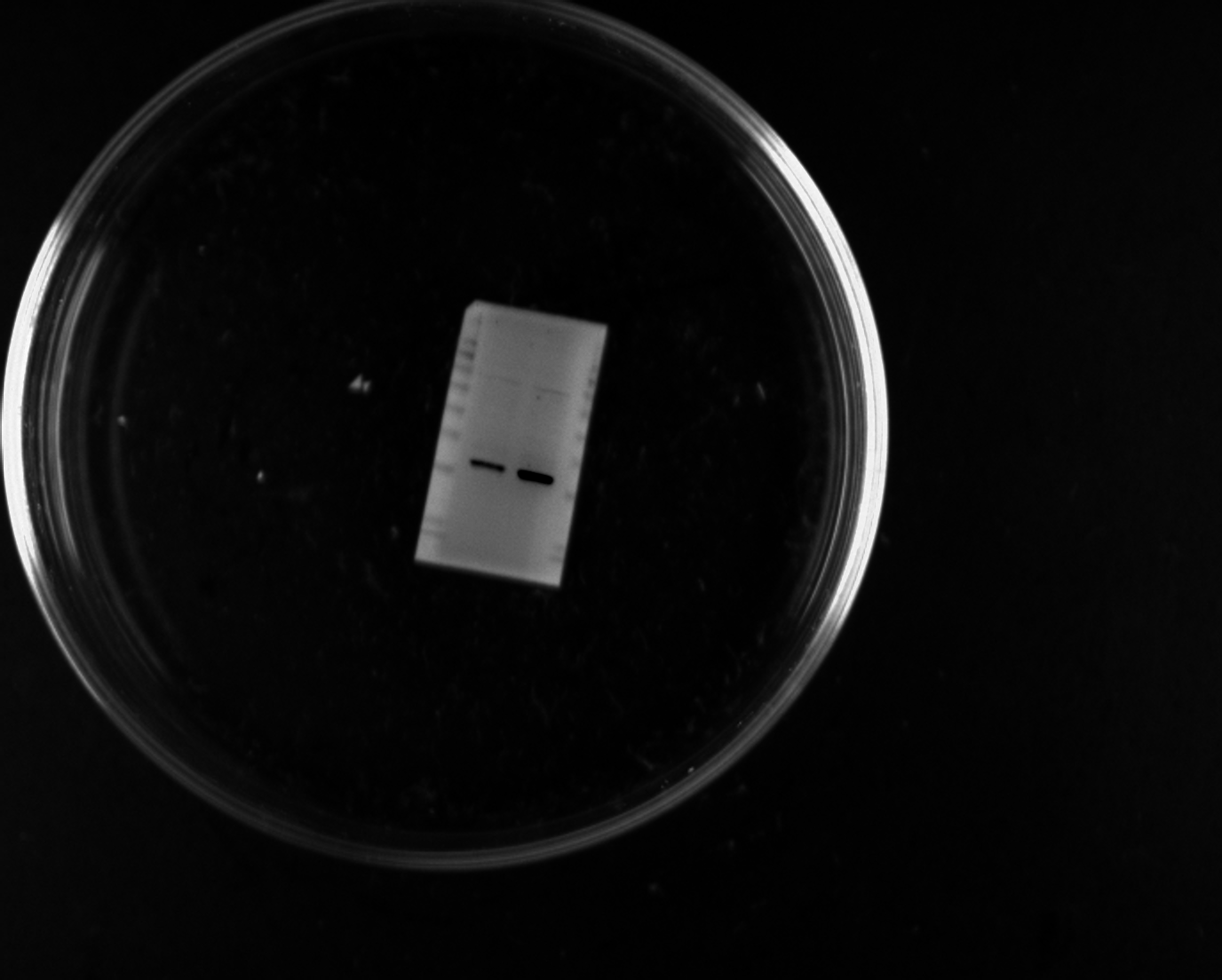


**NCTC1469: β-actin**





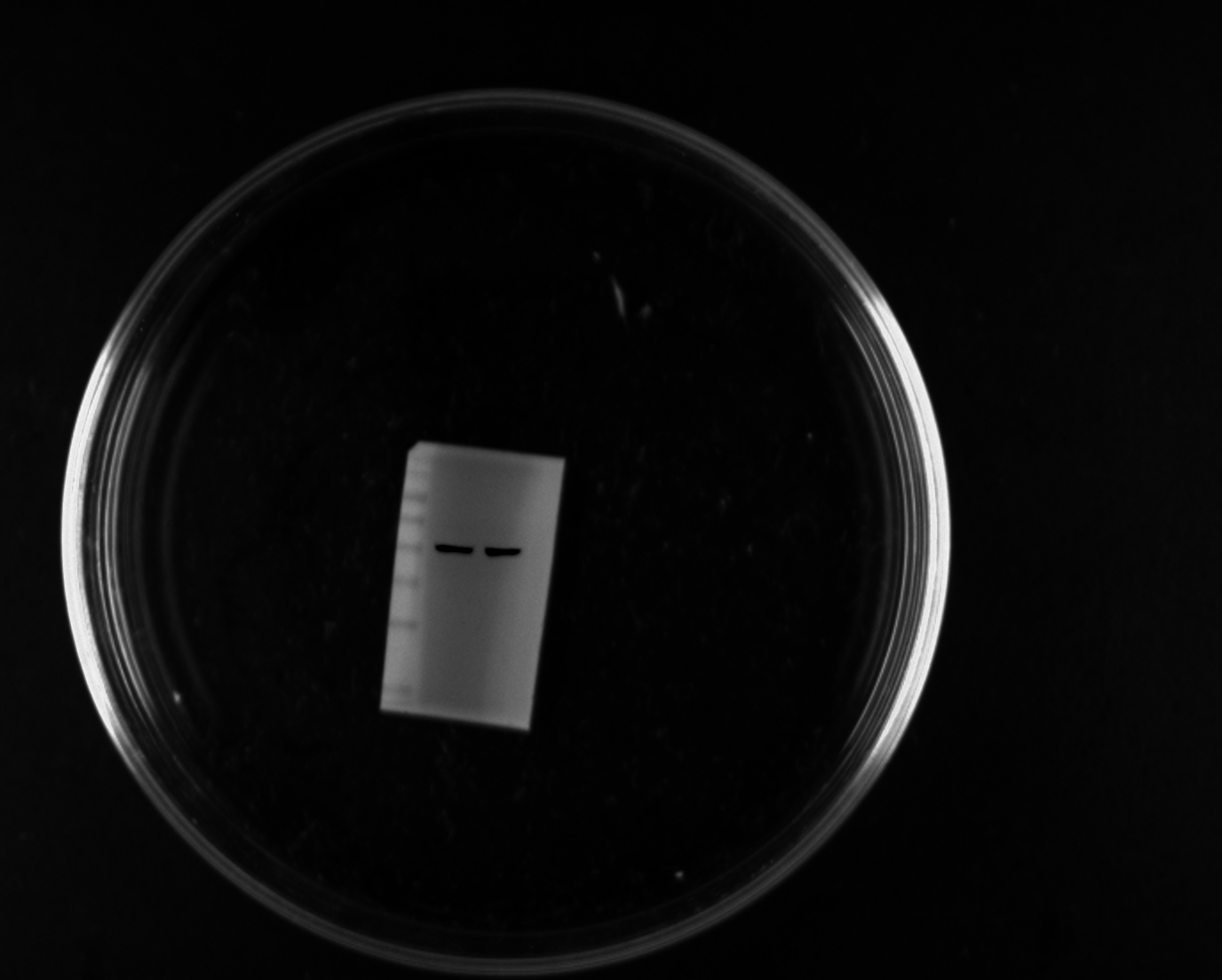


**BNLCL.2: CBX7**


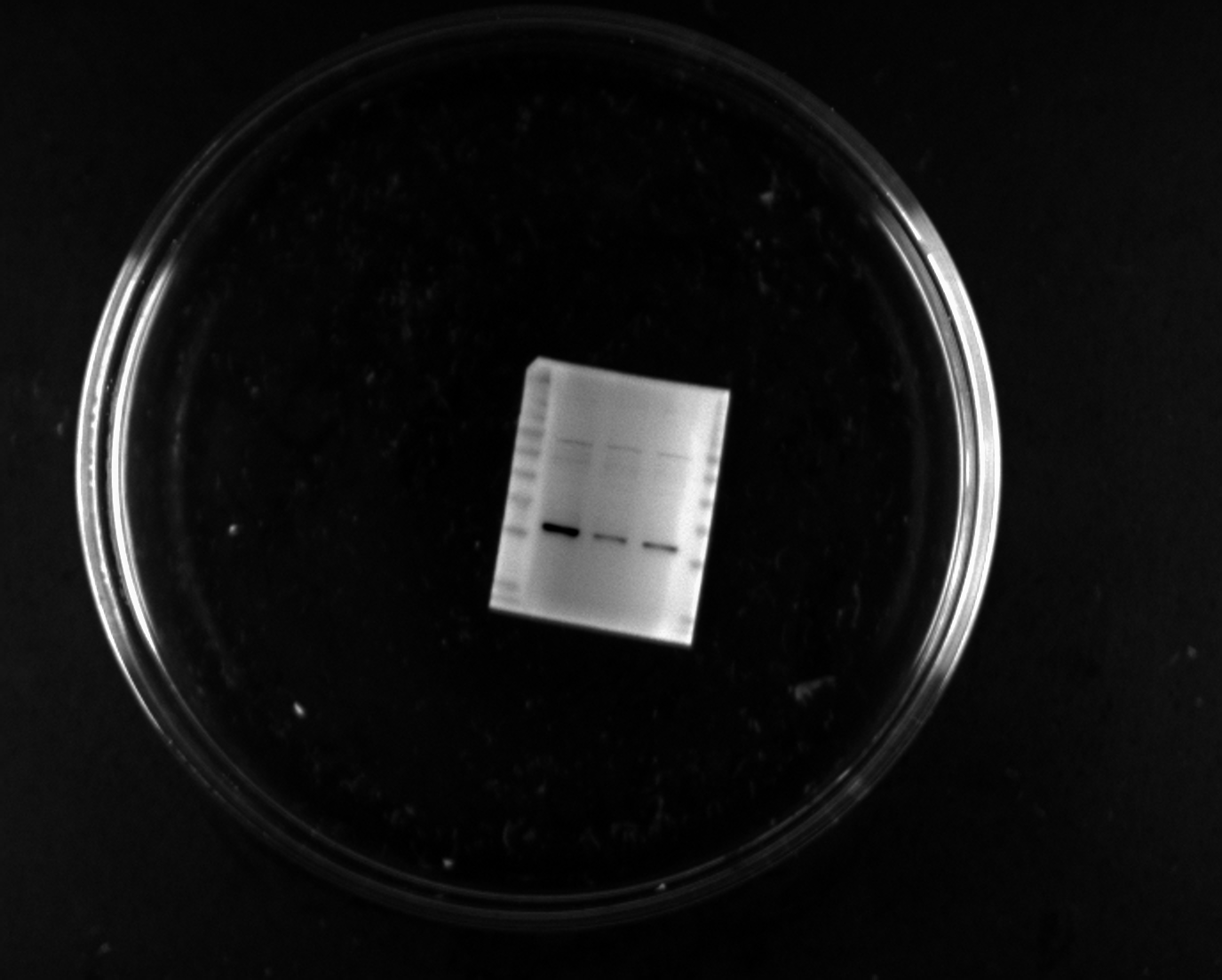

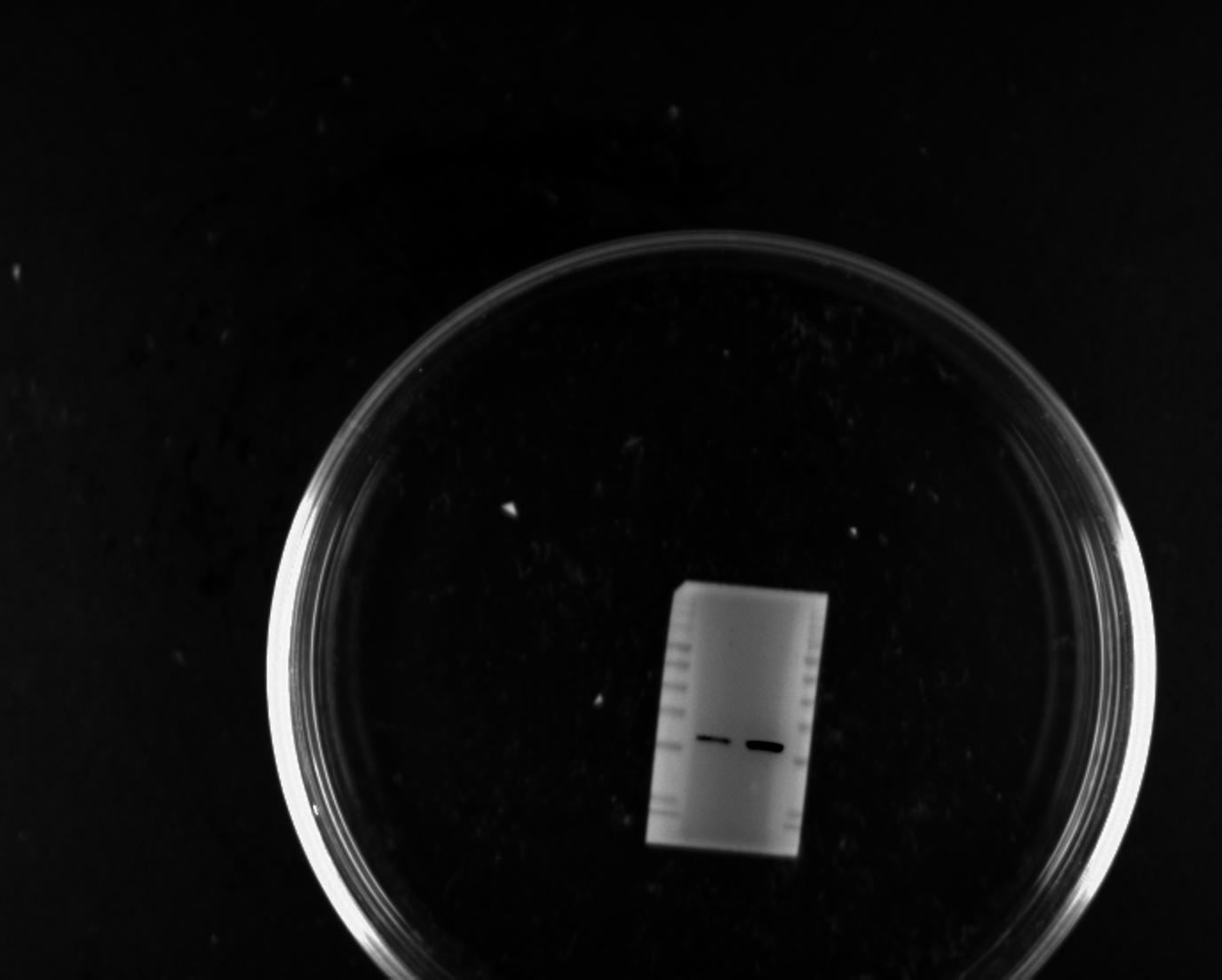


**BNLCL.2: β-actin**





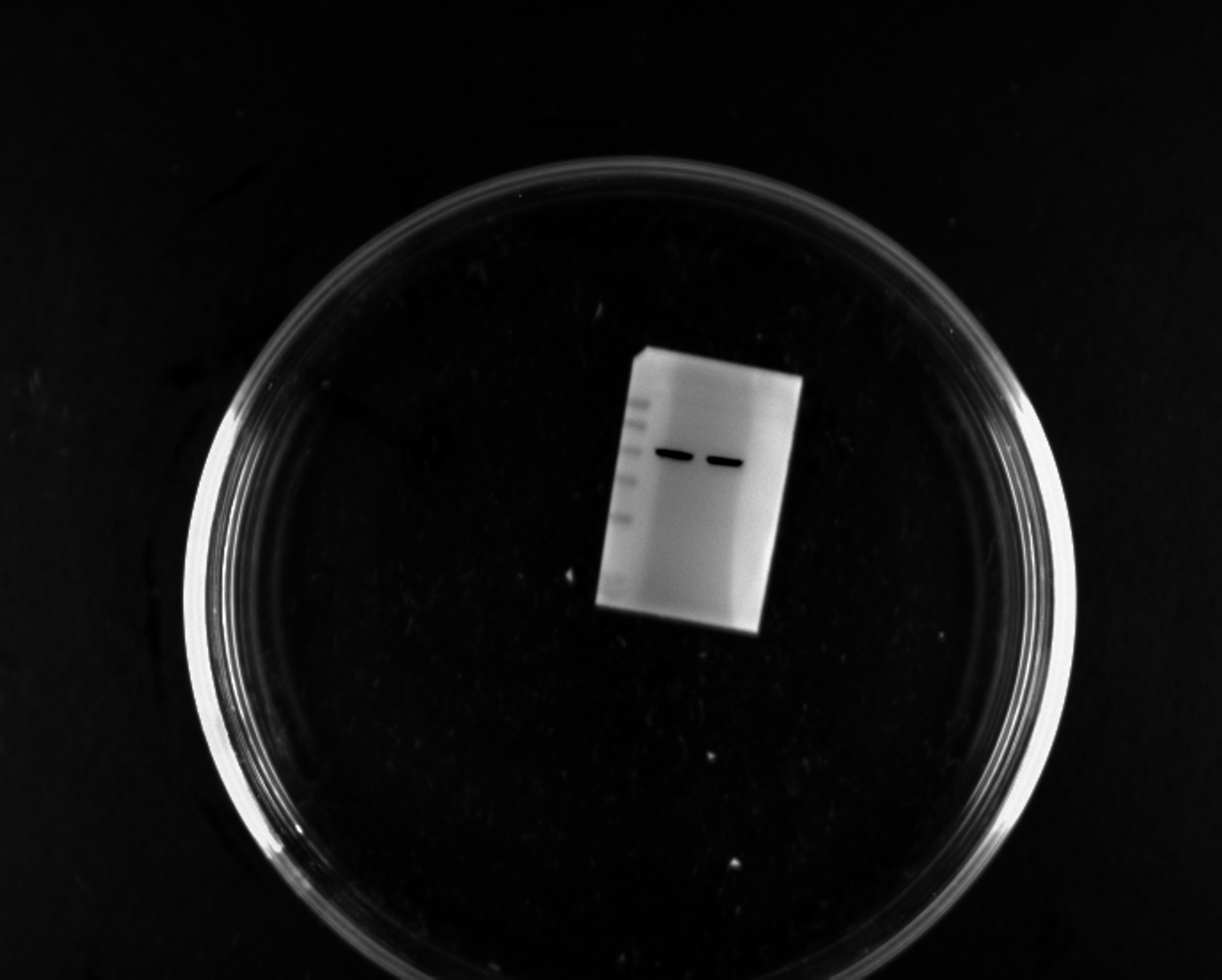


**Figure 1I**

**NCTC1469: CyclinD1**


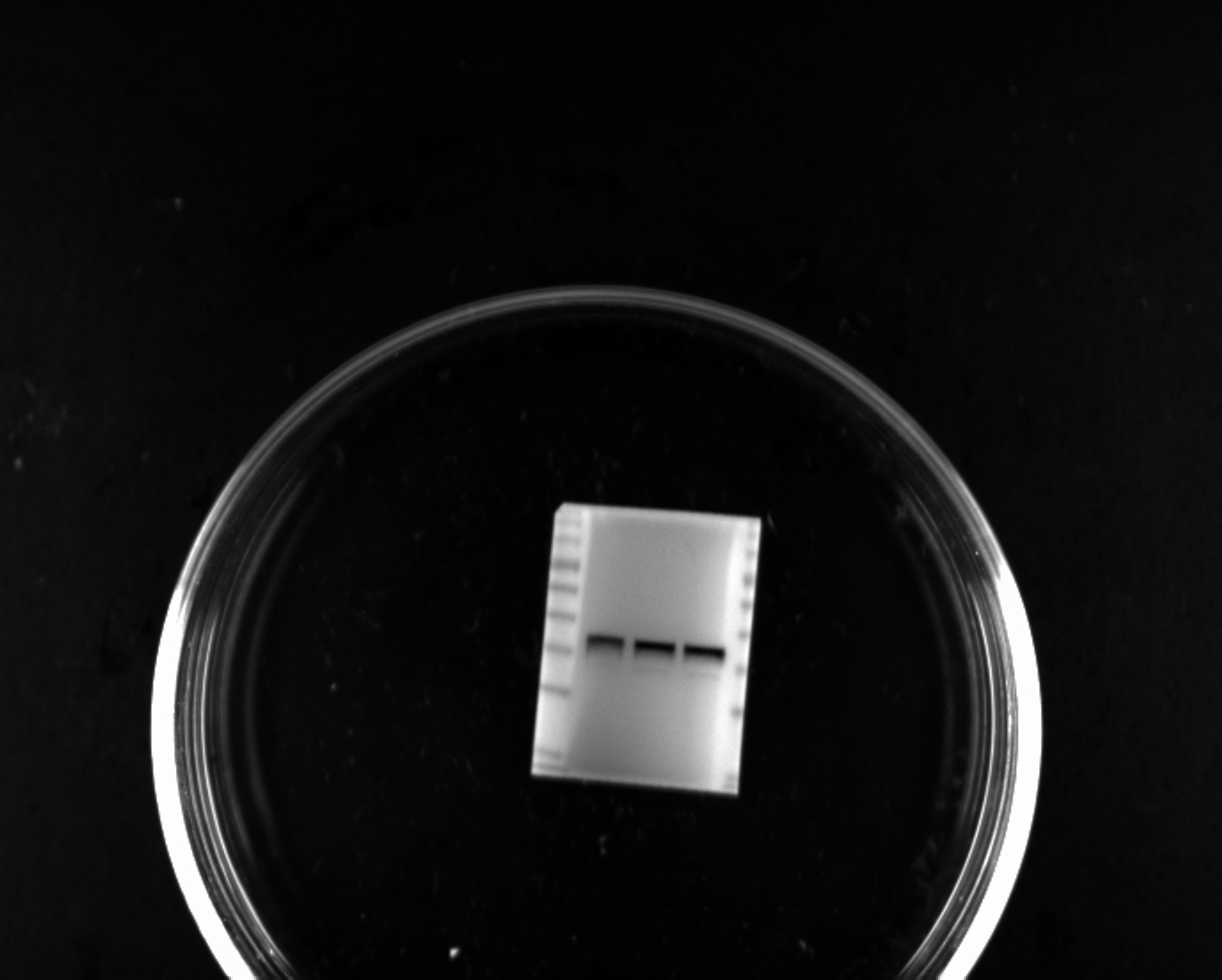

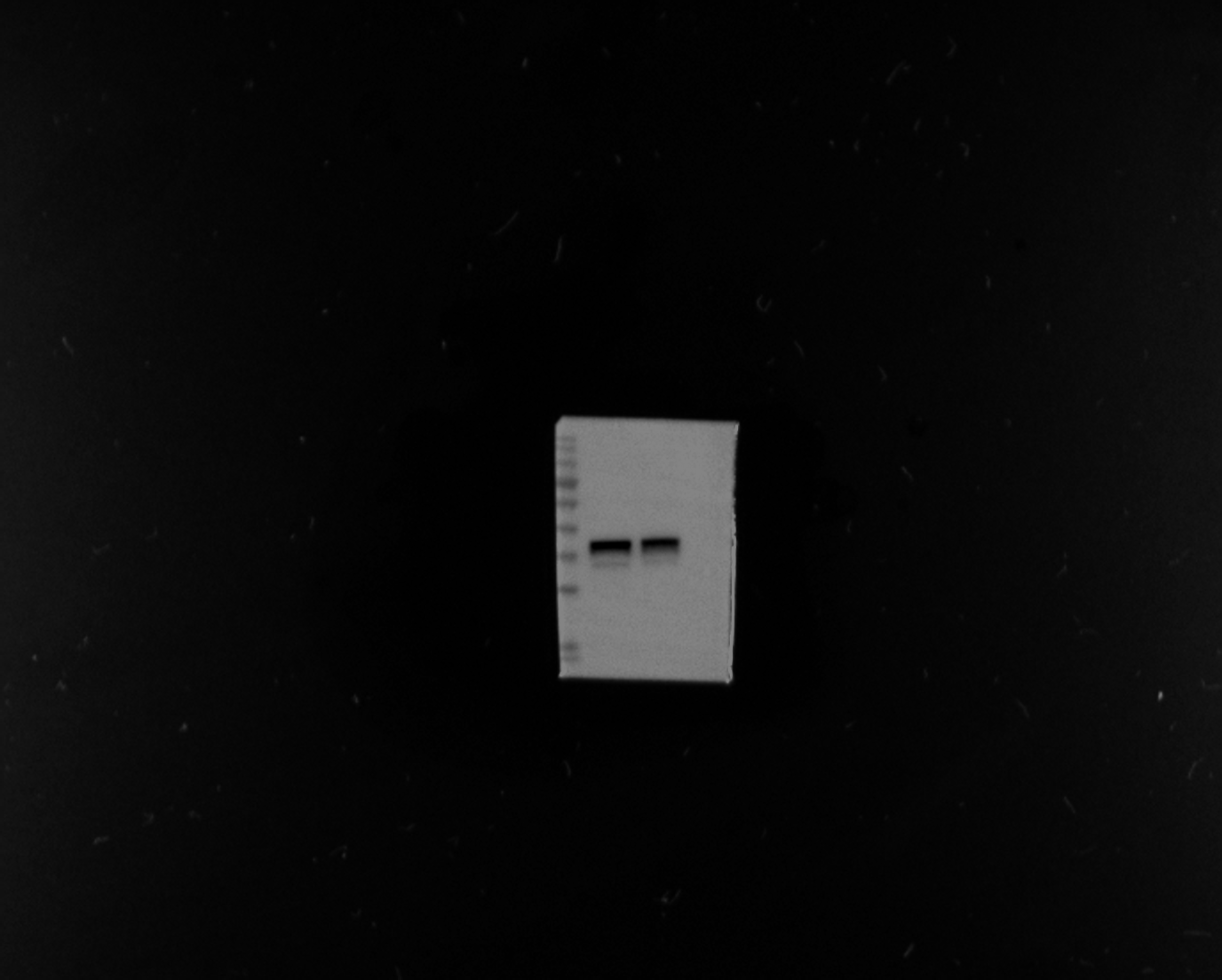


**NCTC1469: CyclinE**


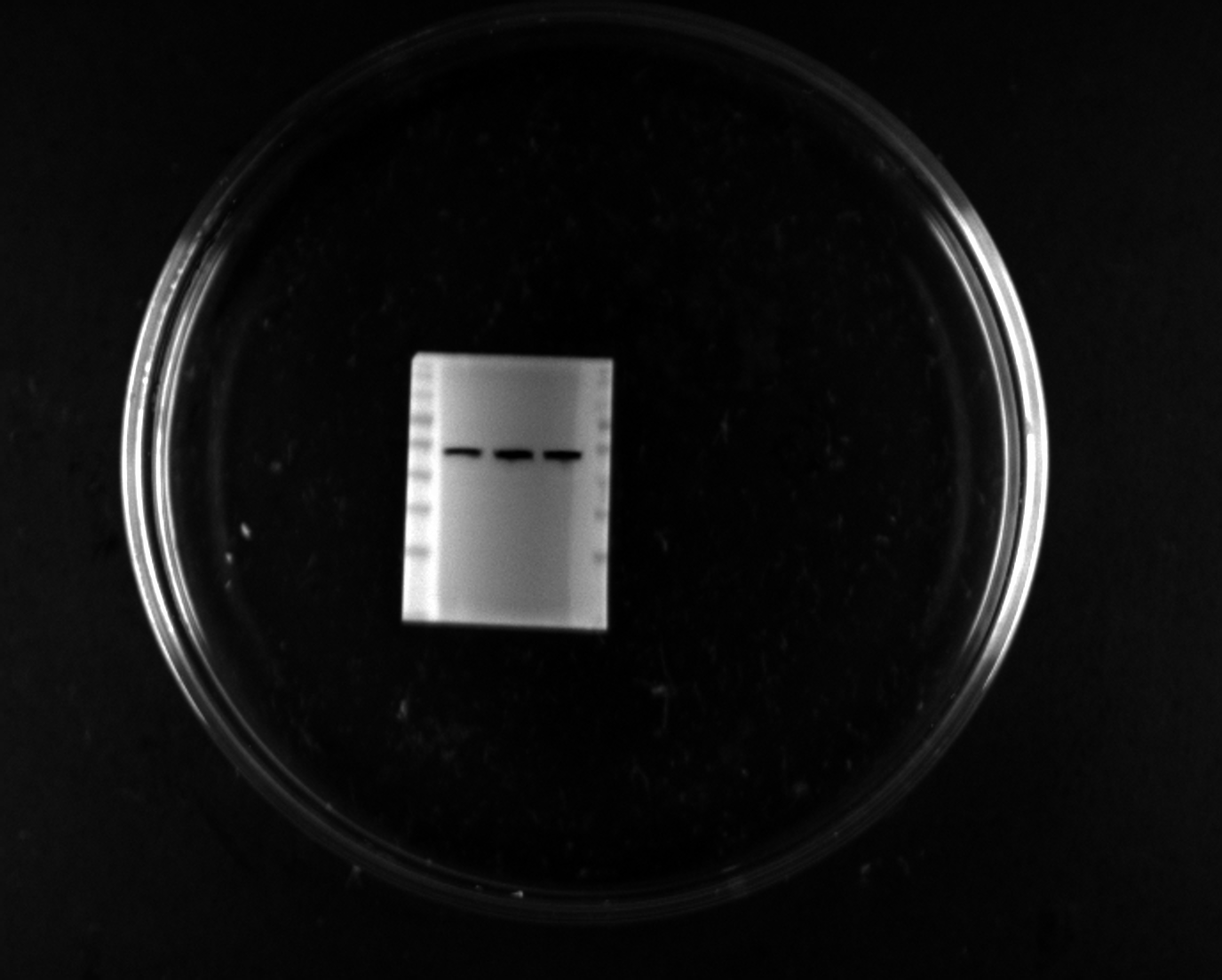

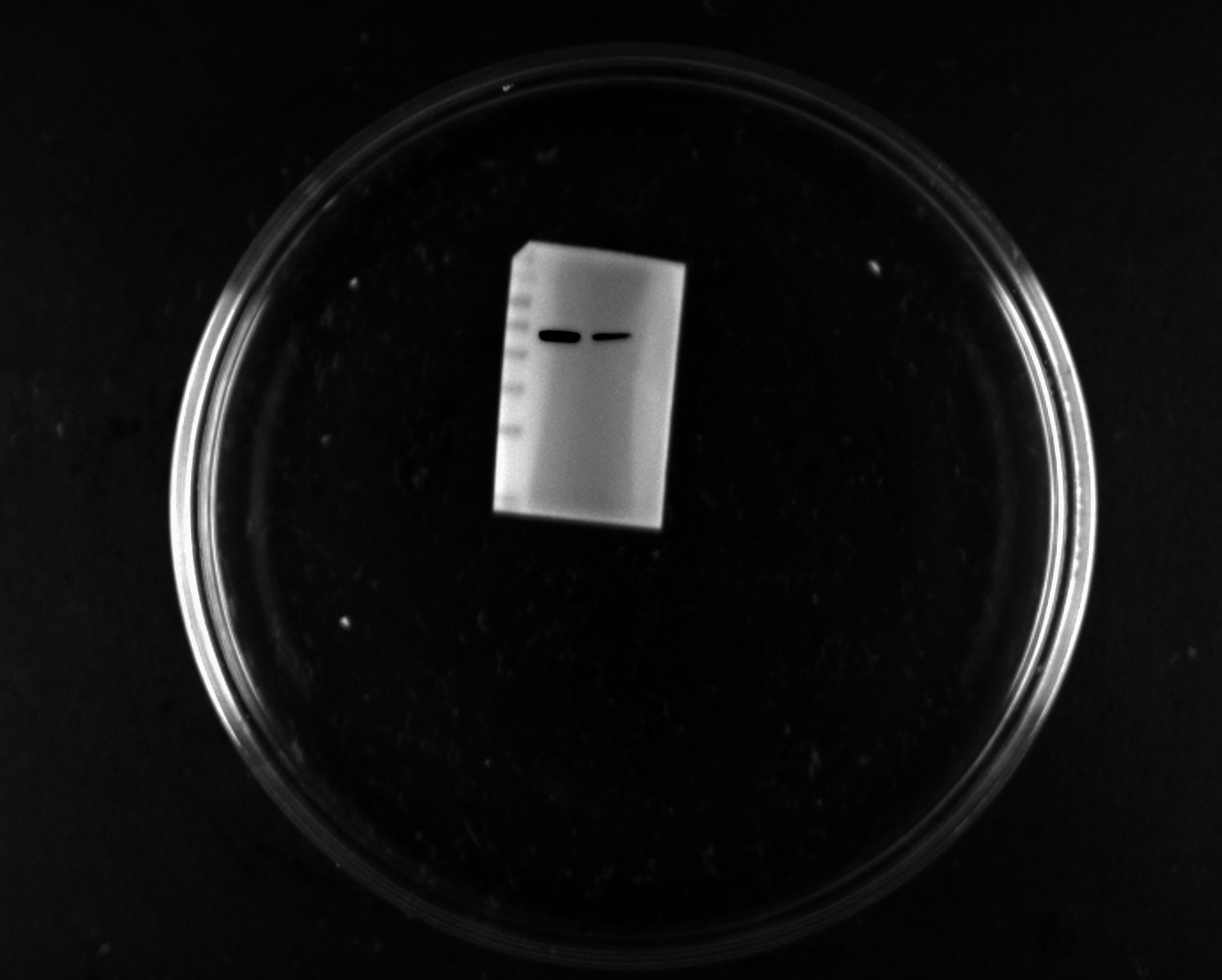


**NCTC1469: β-actin**


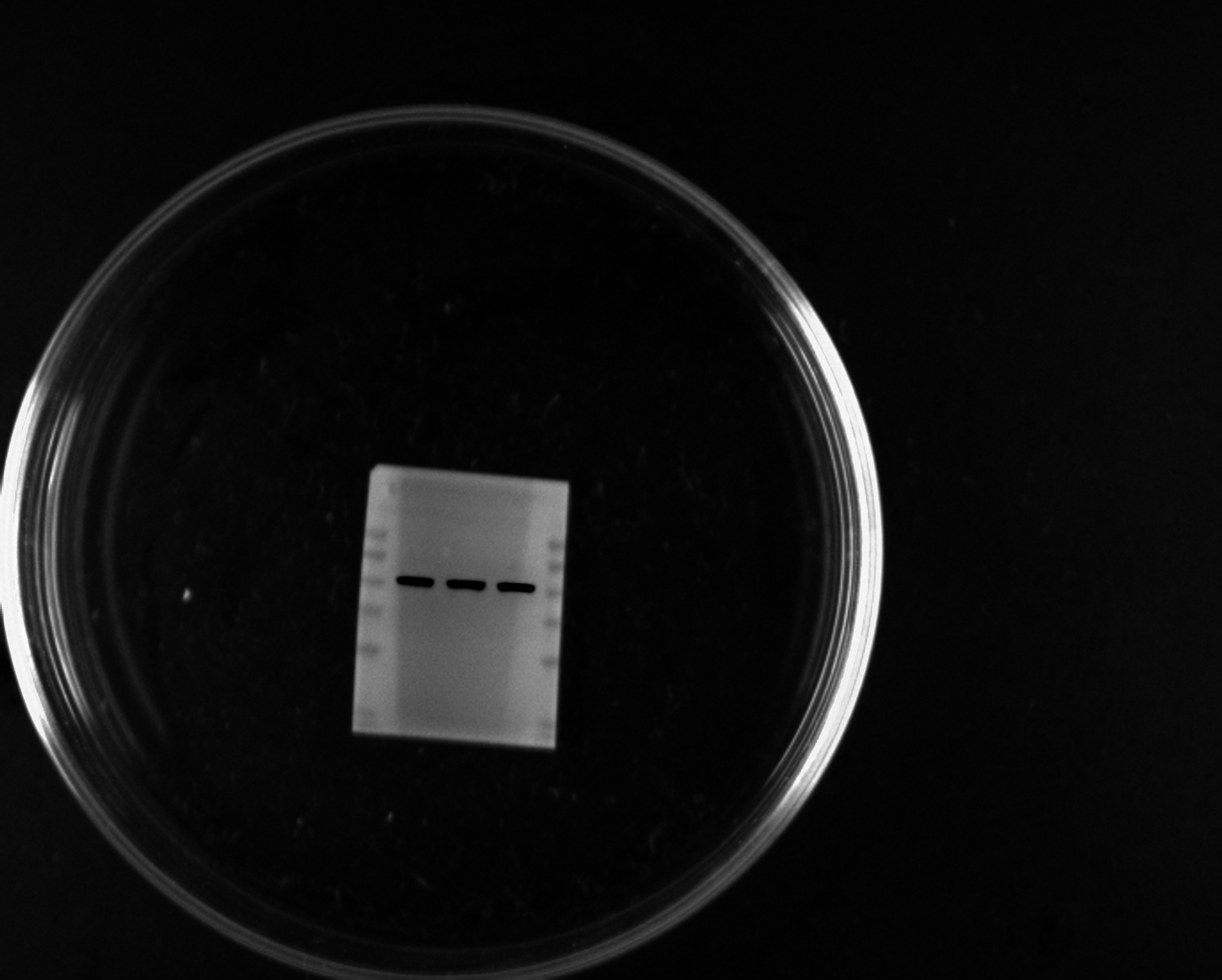

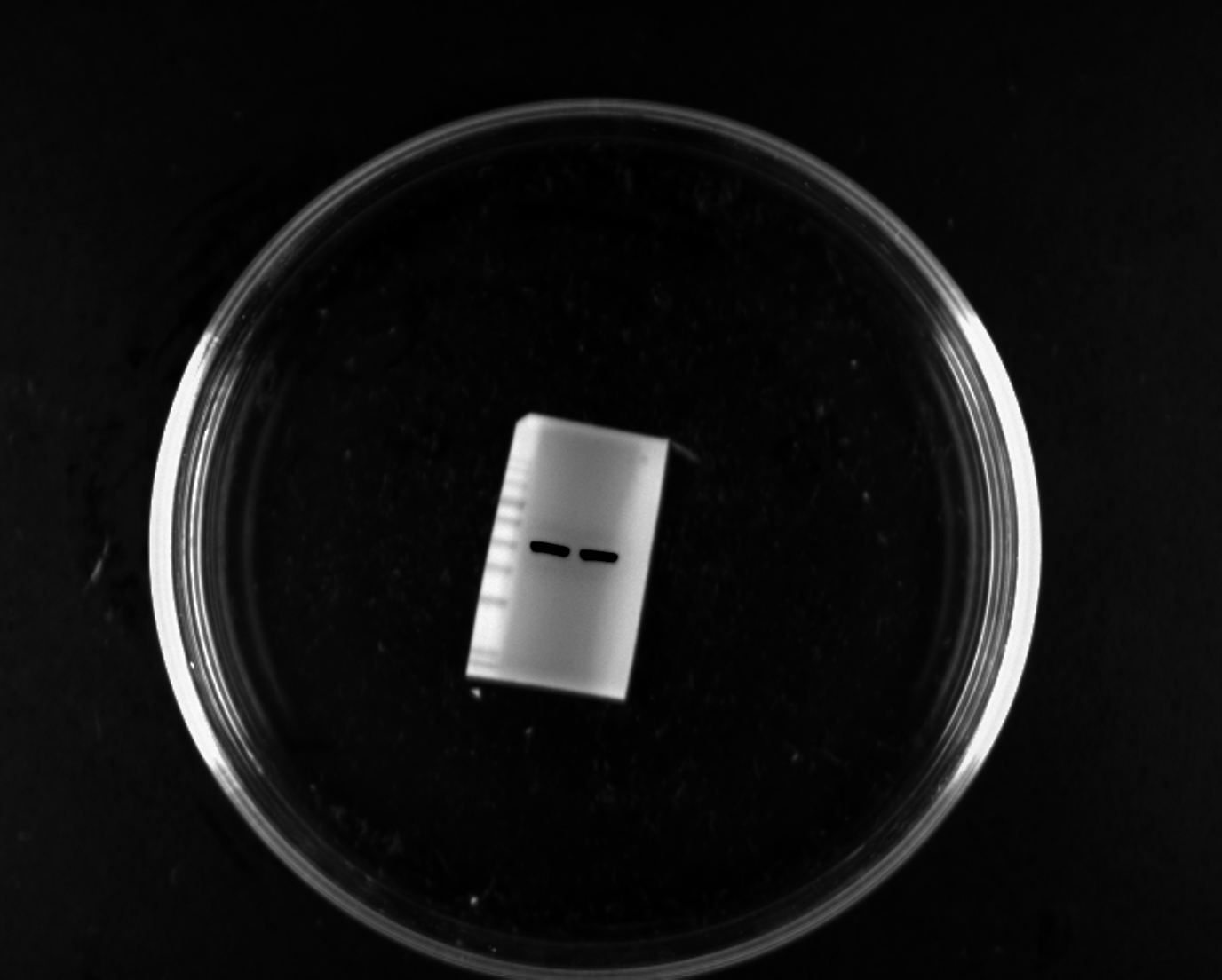


**BNLCL.2: CyclinD1**


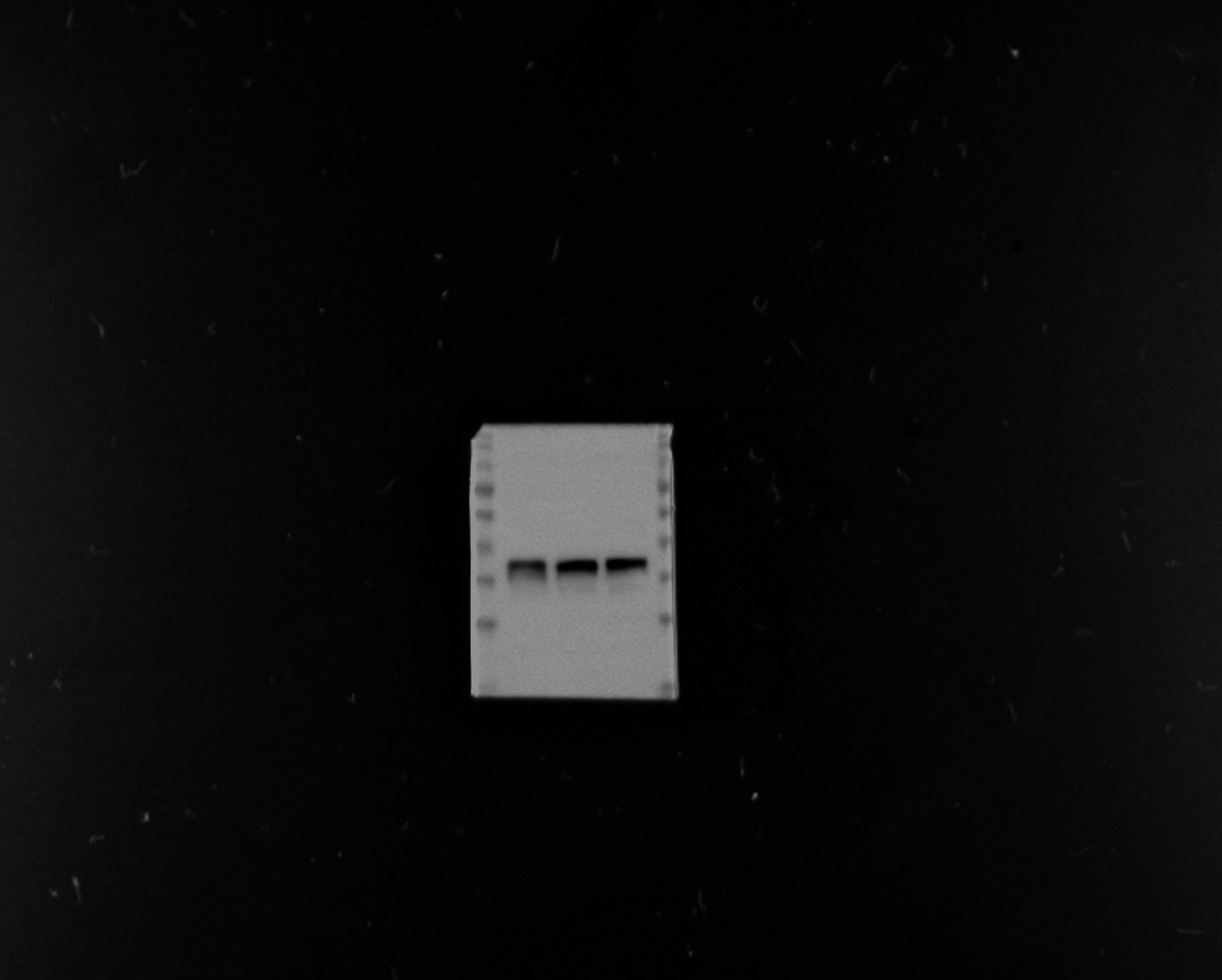

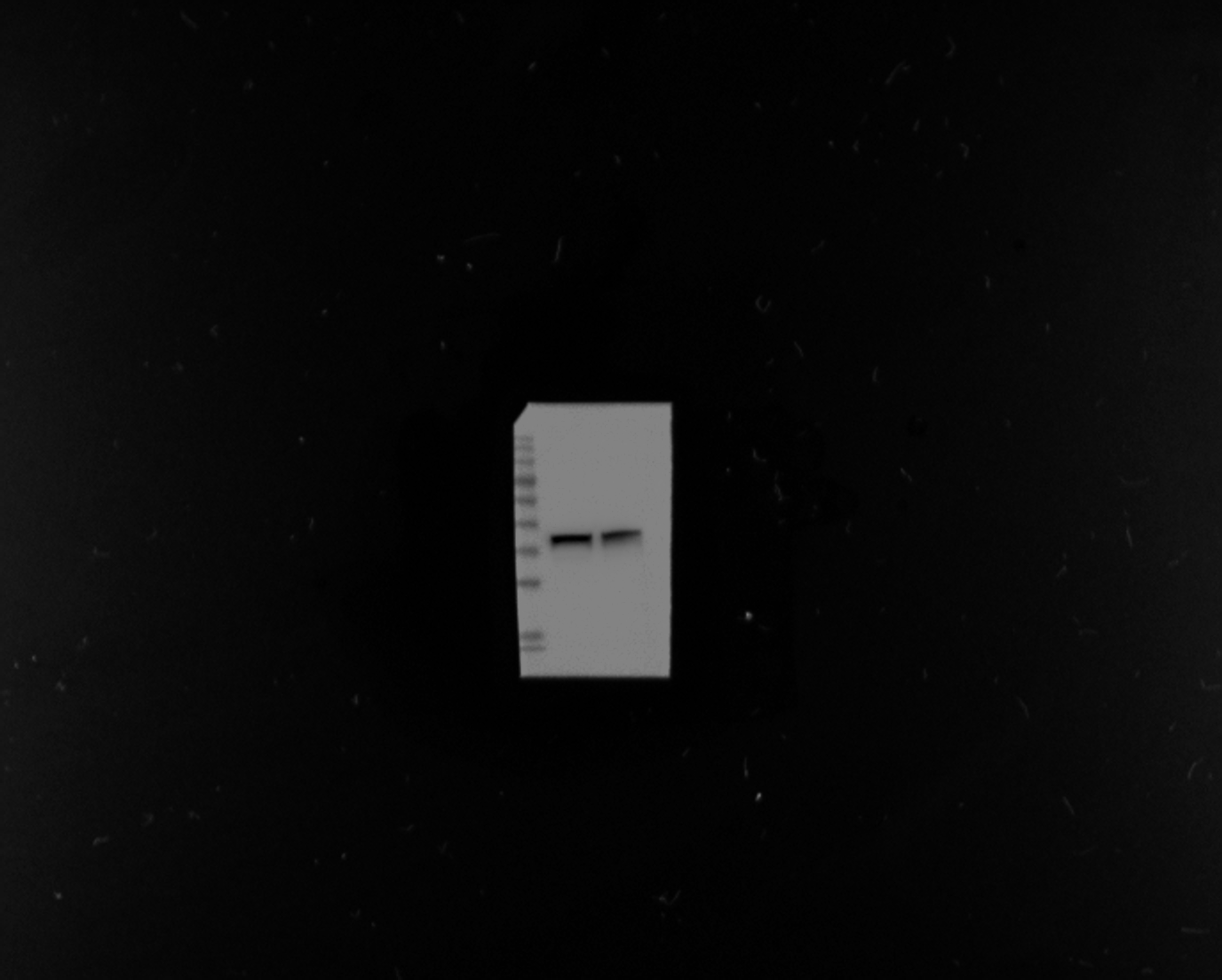


**BNLCL.2: CyclinE**


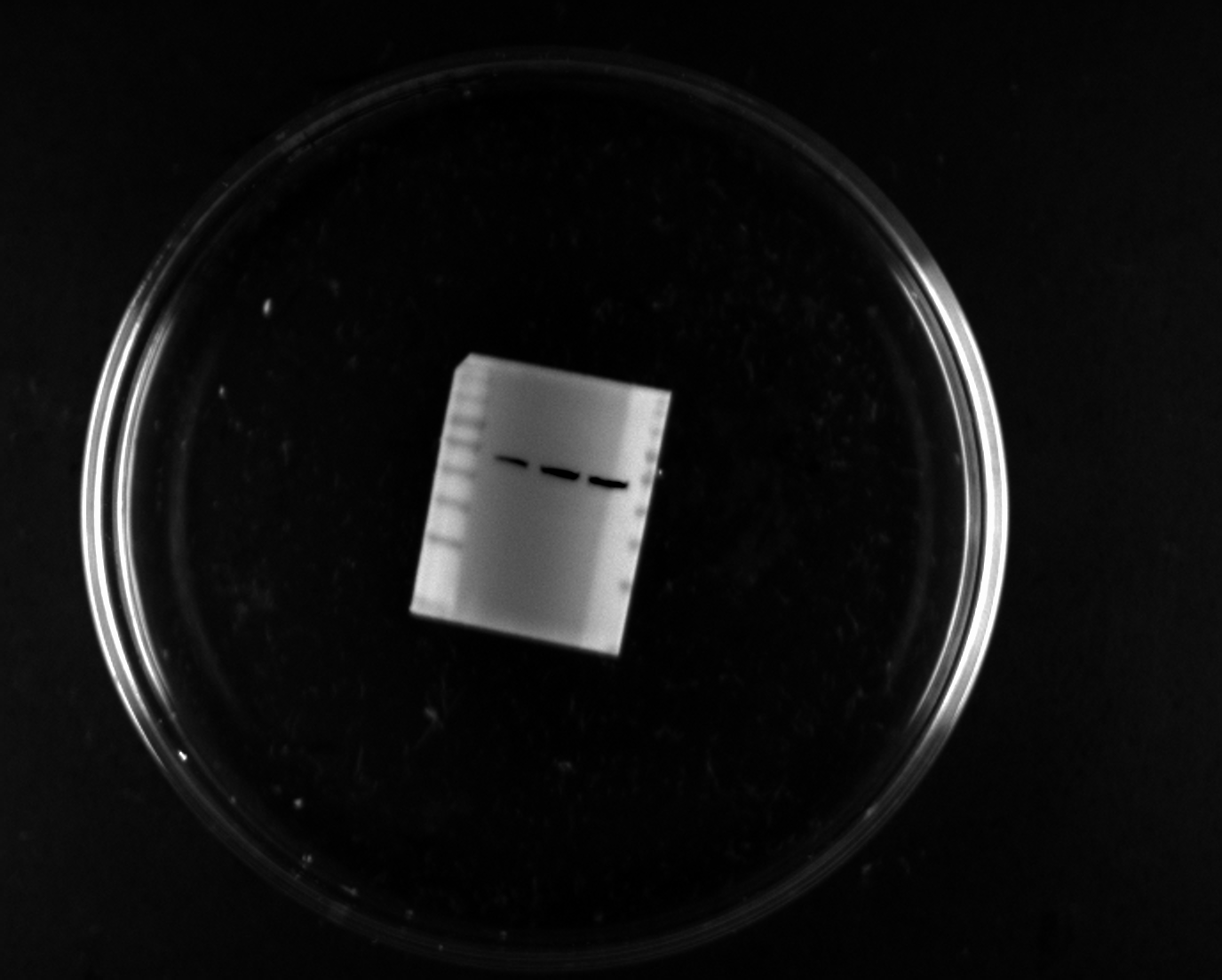

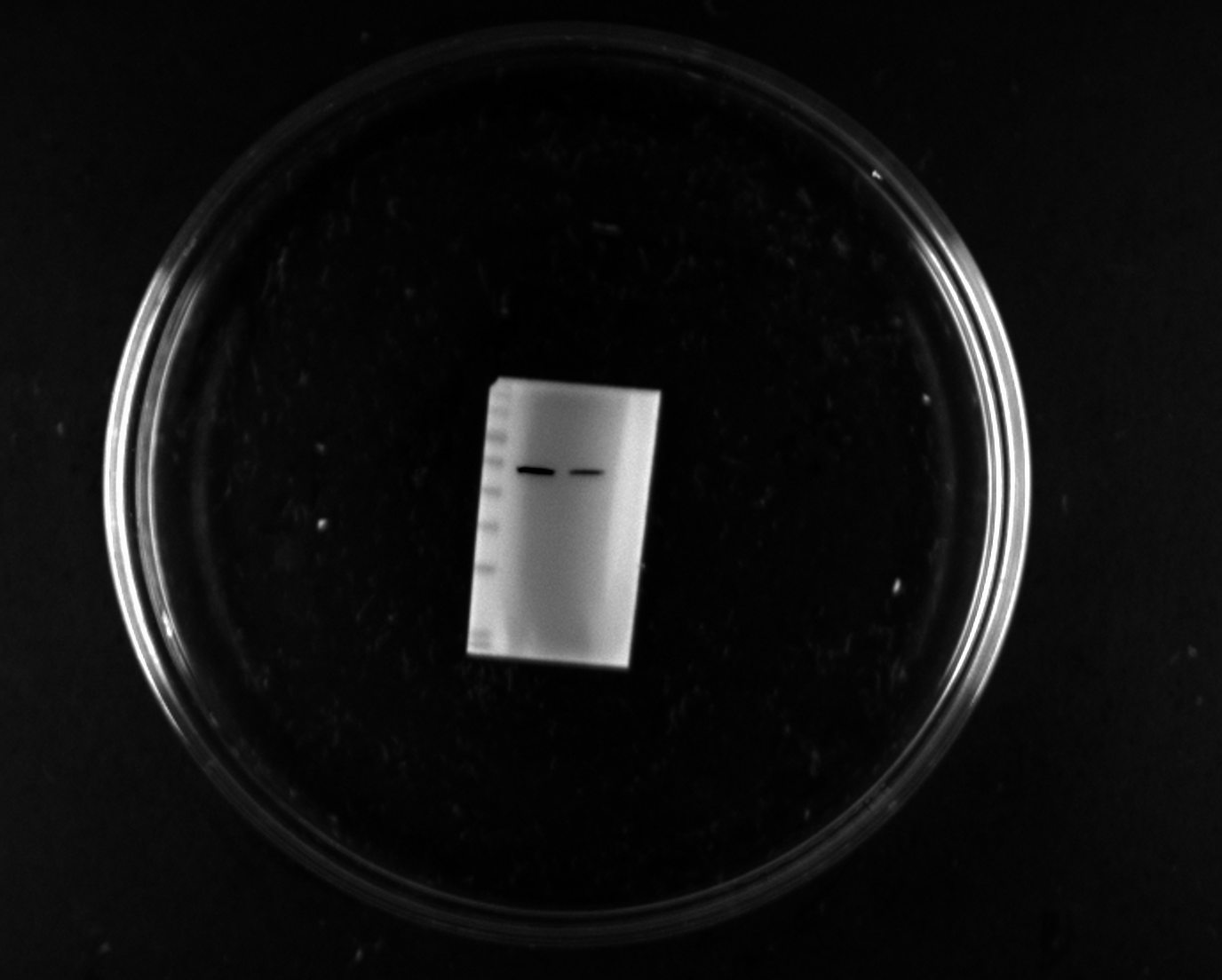


**BNLCL.2: β-actin**


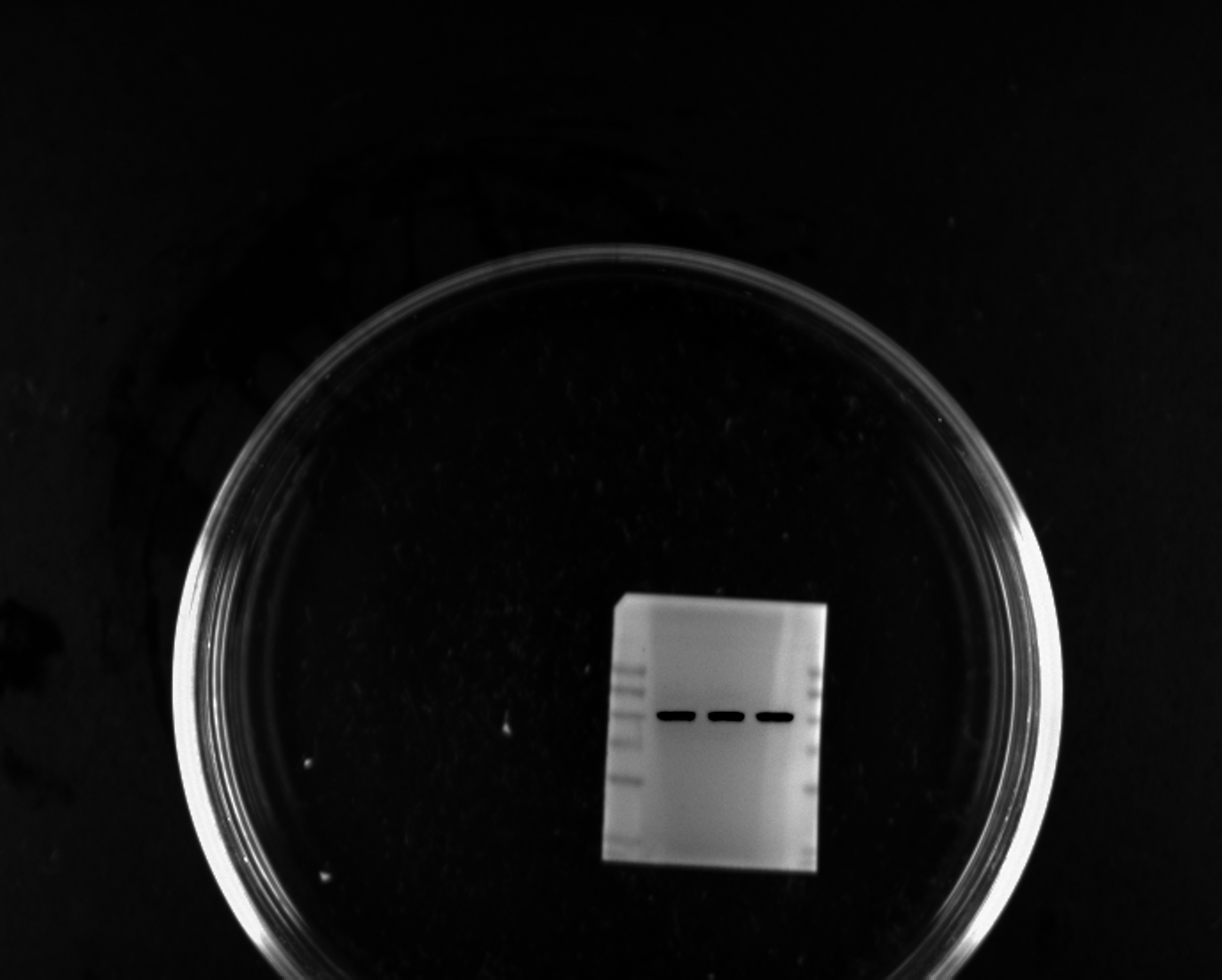

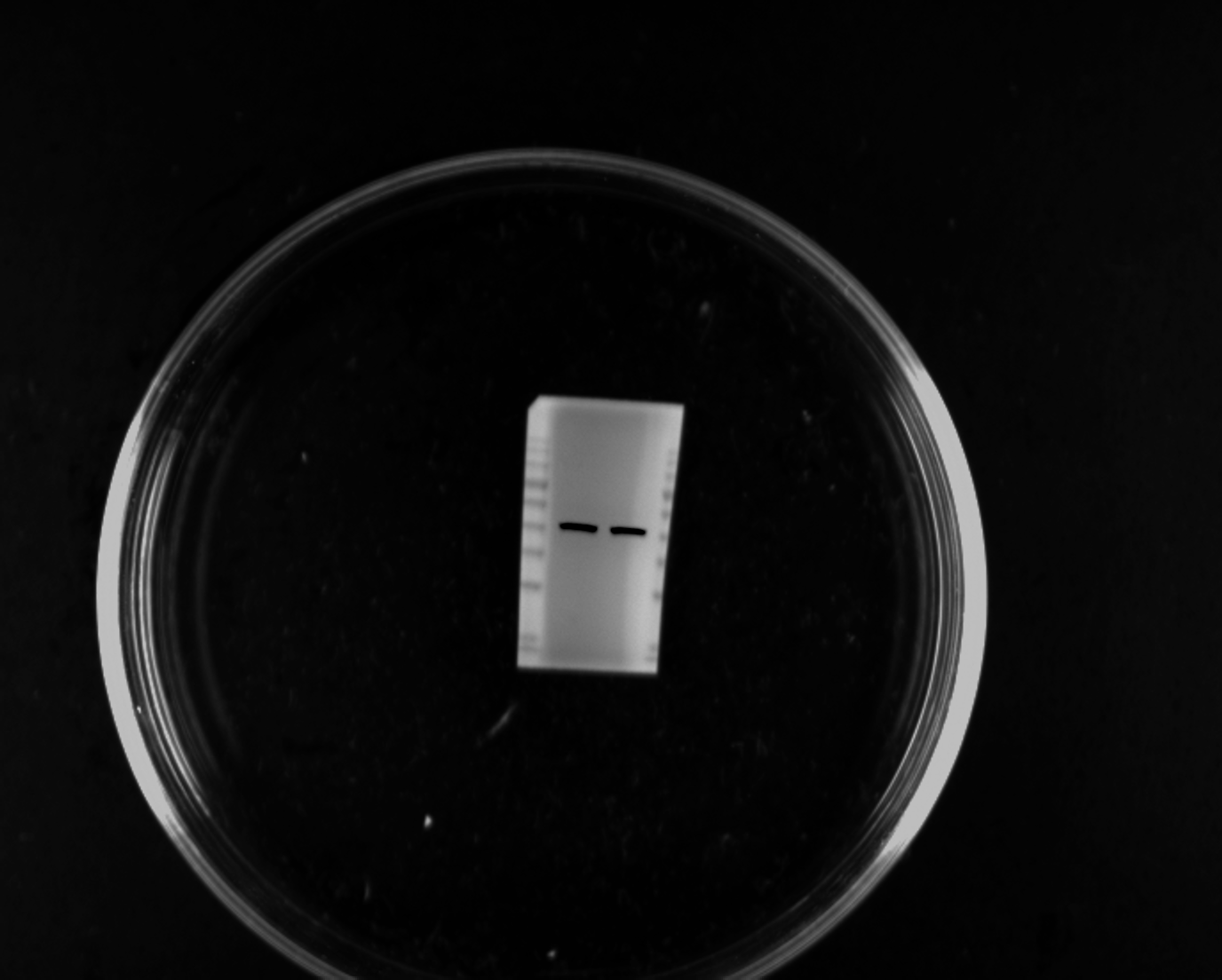


**Figure 2B**

**NCTC1469:**


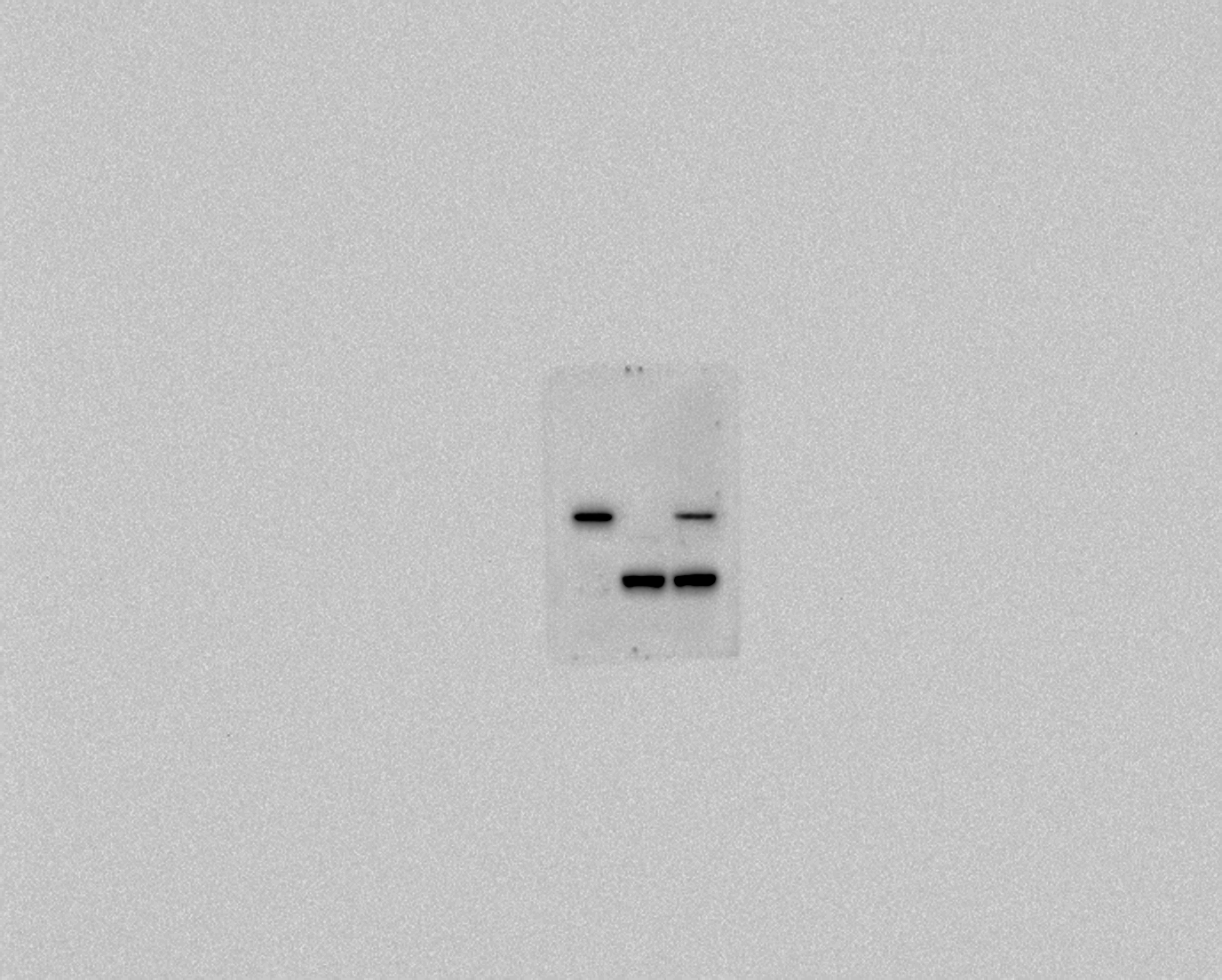

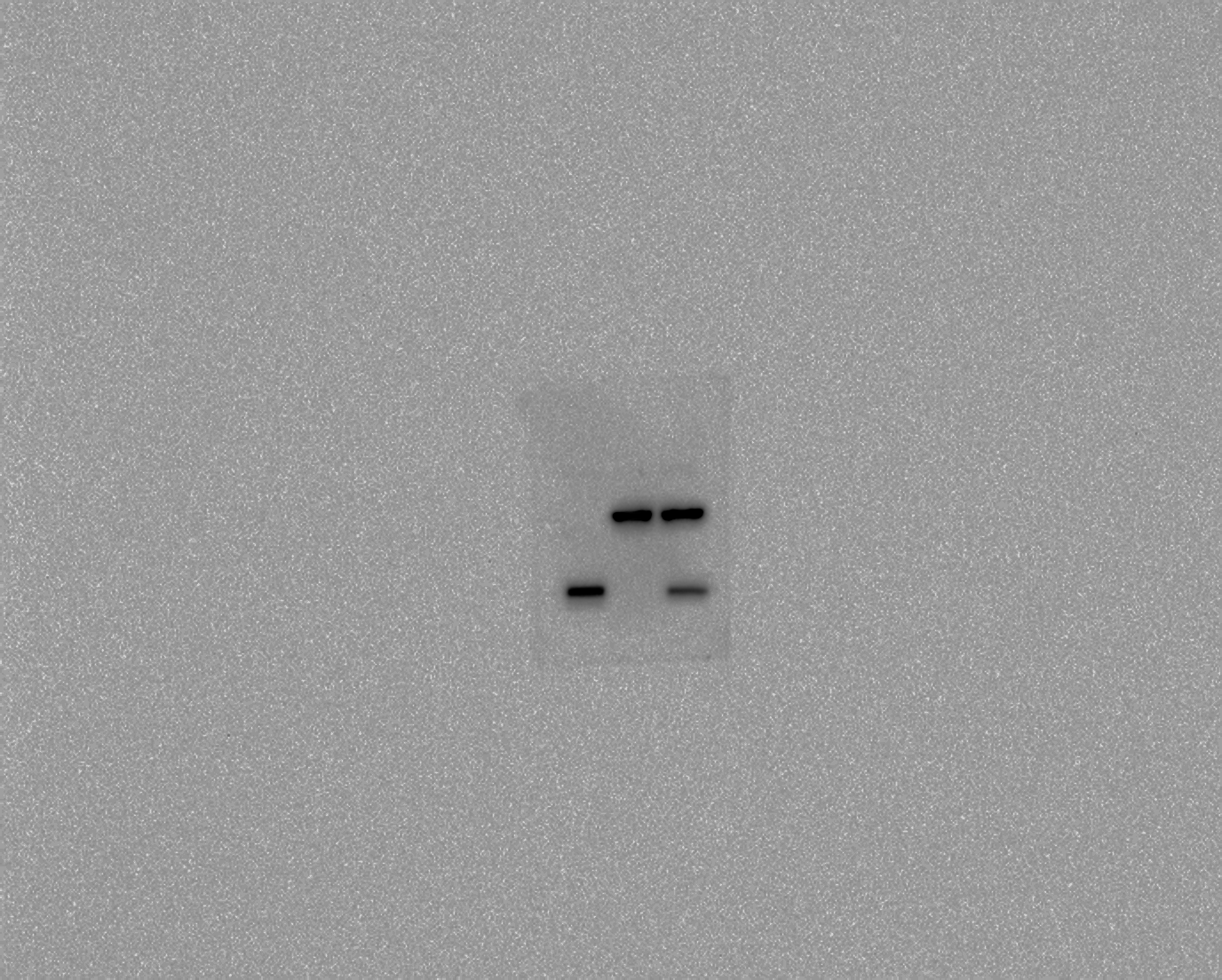


**BNLCL.2:**


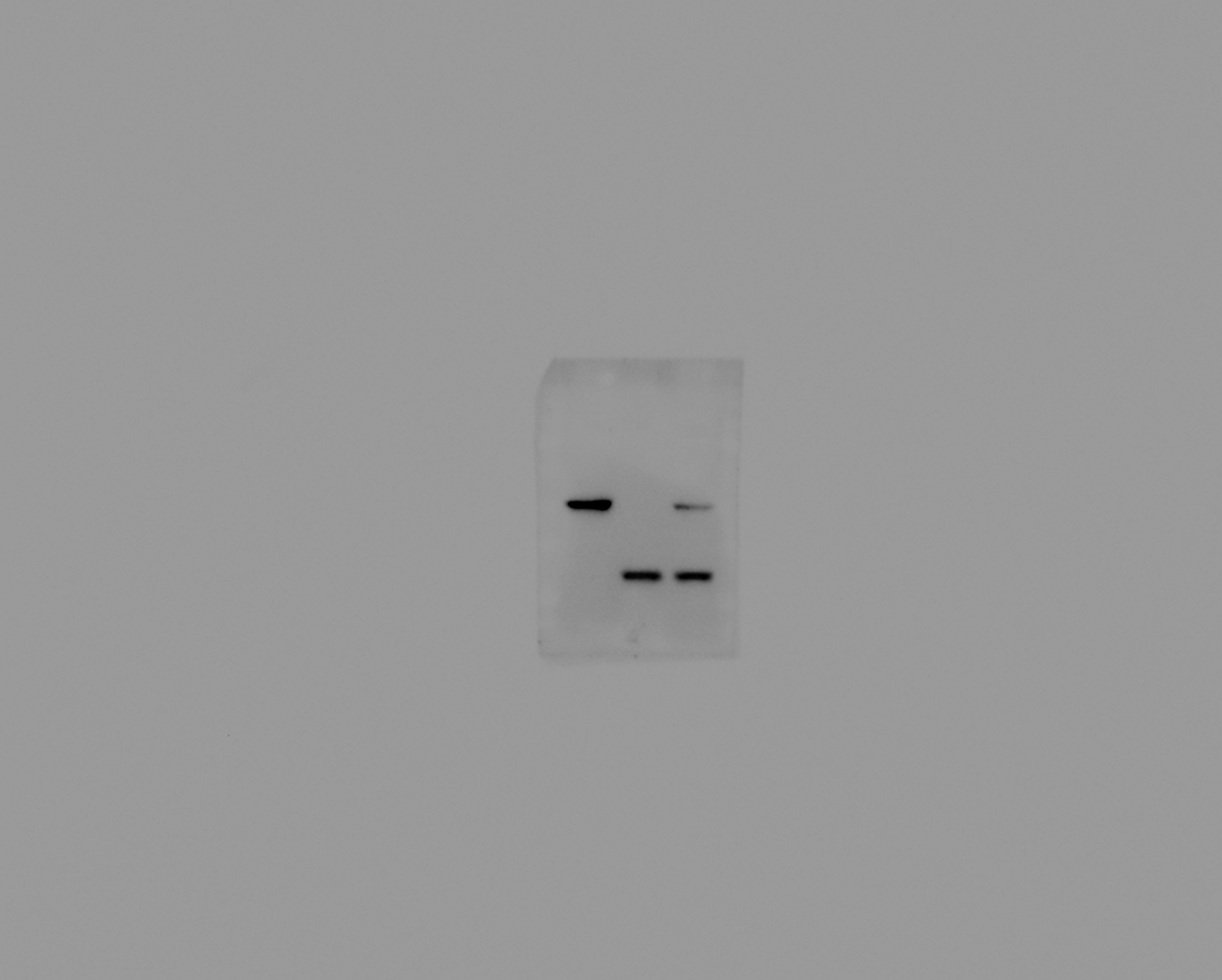

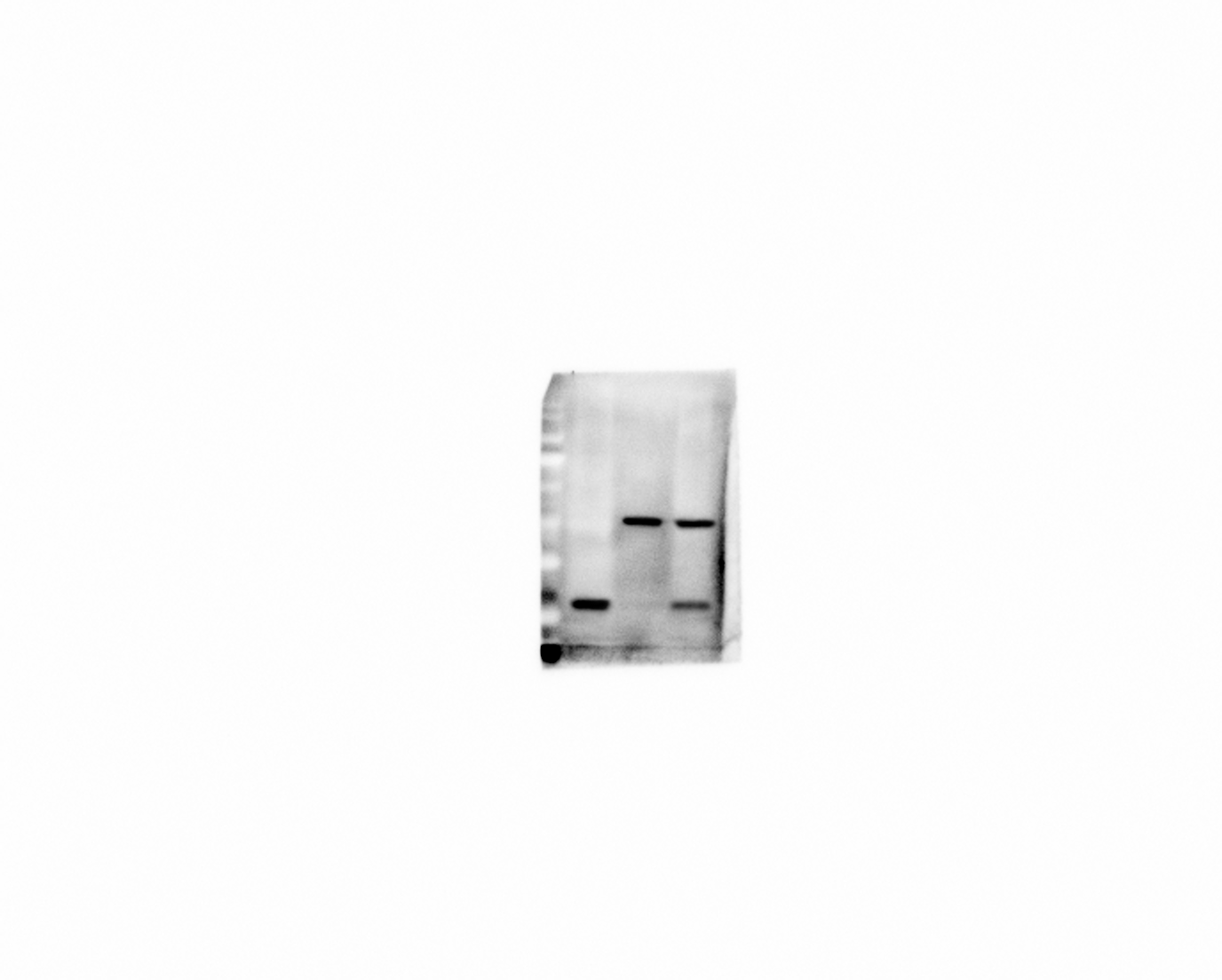


**Figure 2E**

**NCTC1469: BMI1**


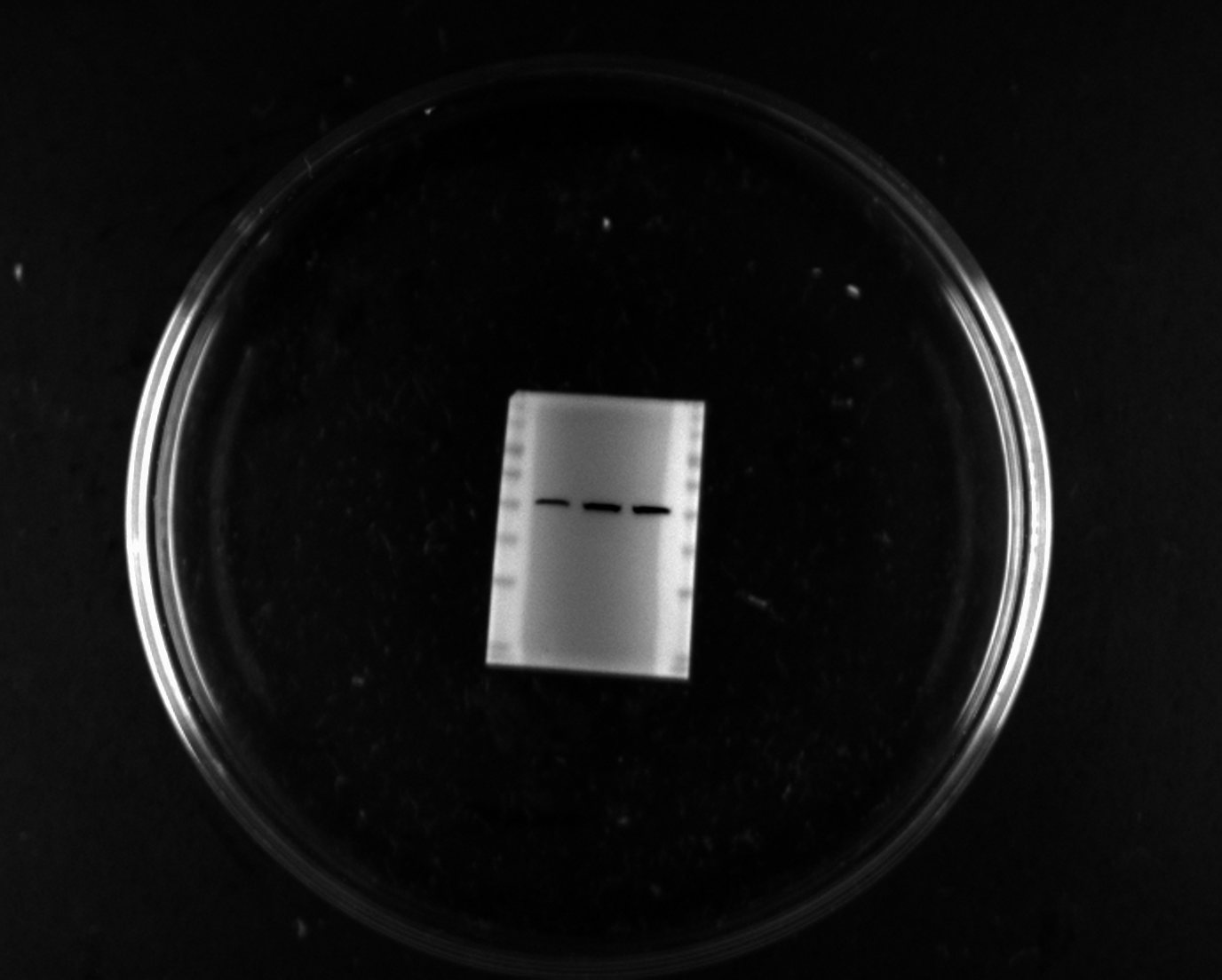

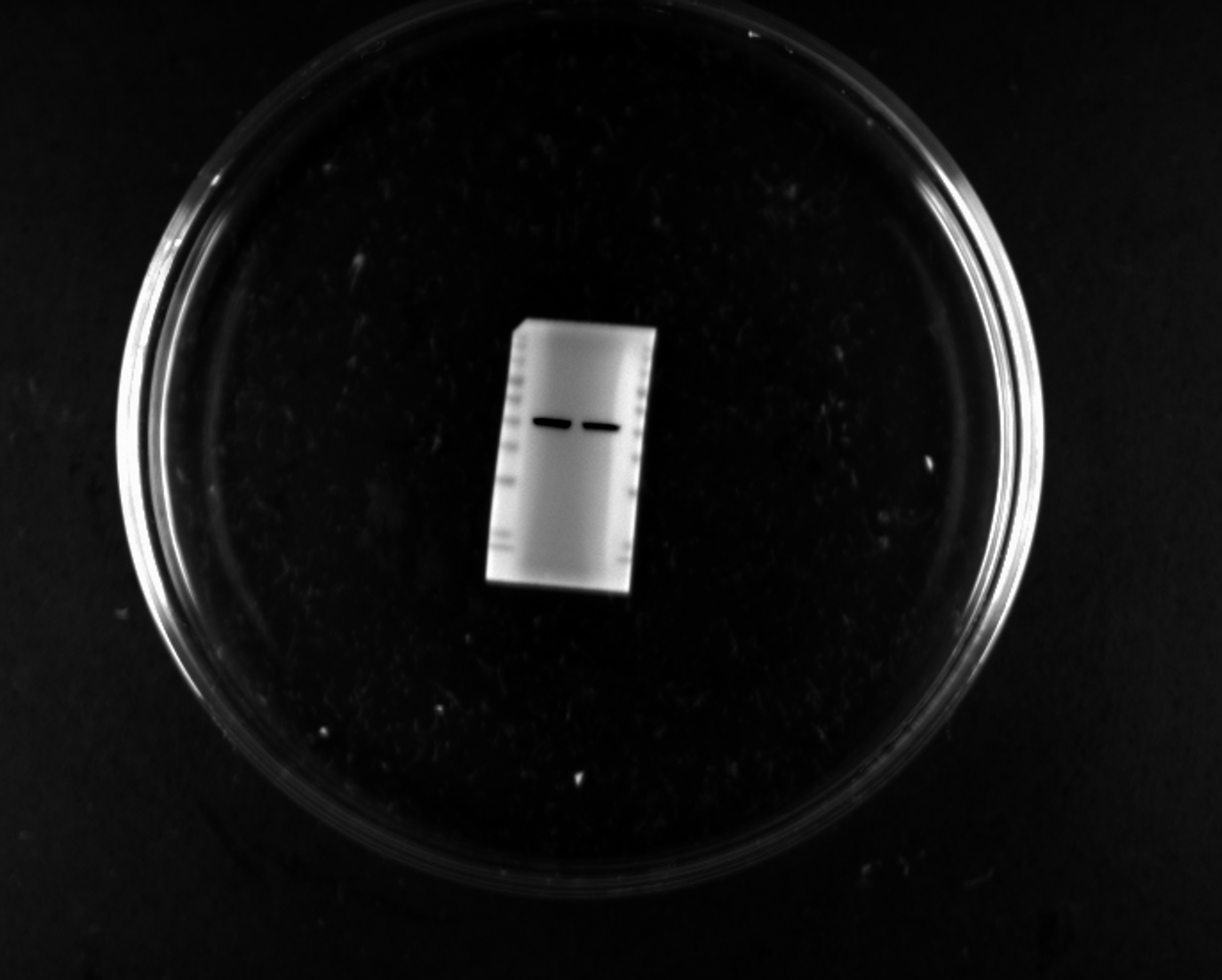


**NCTC1469: β-actin**


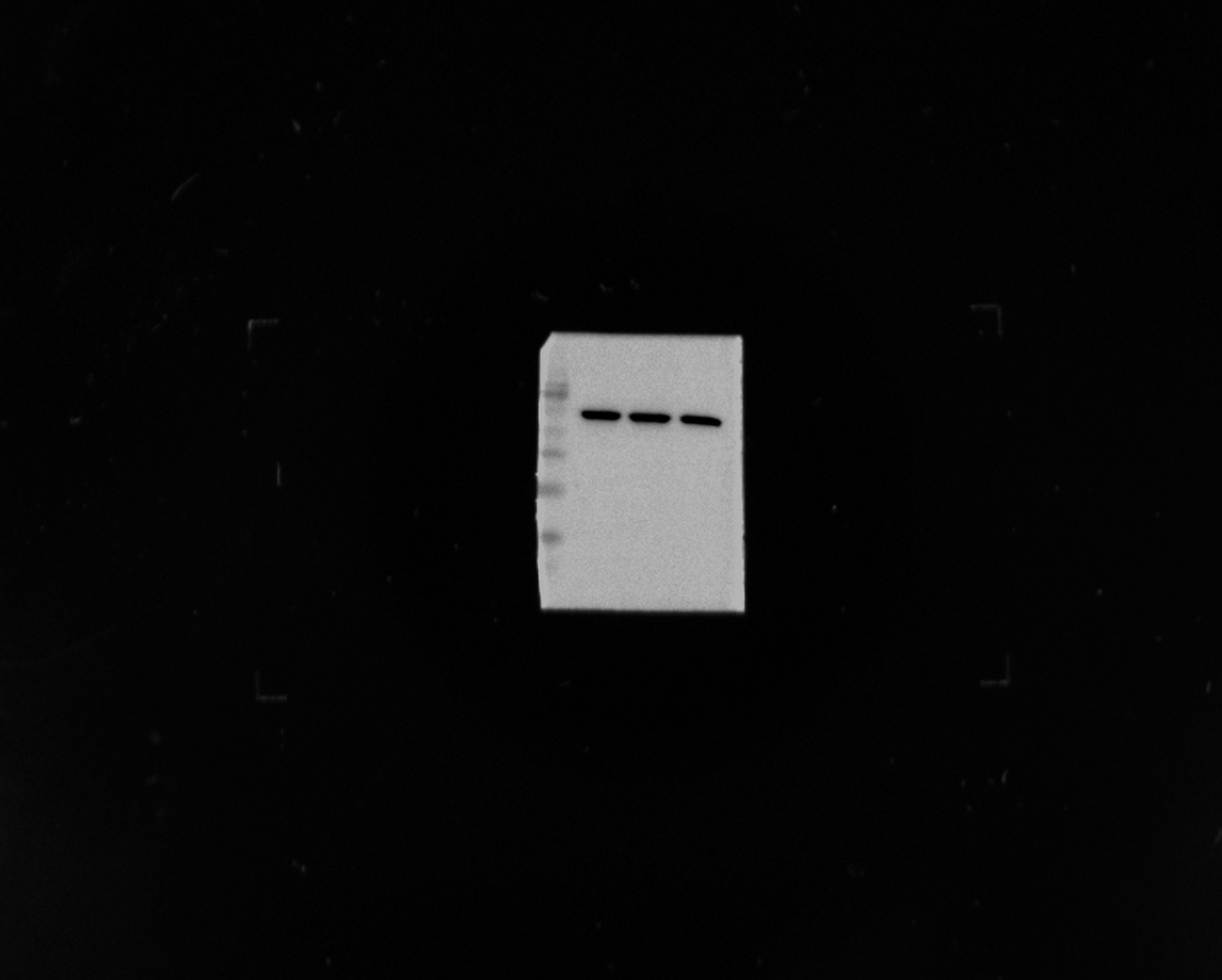

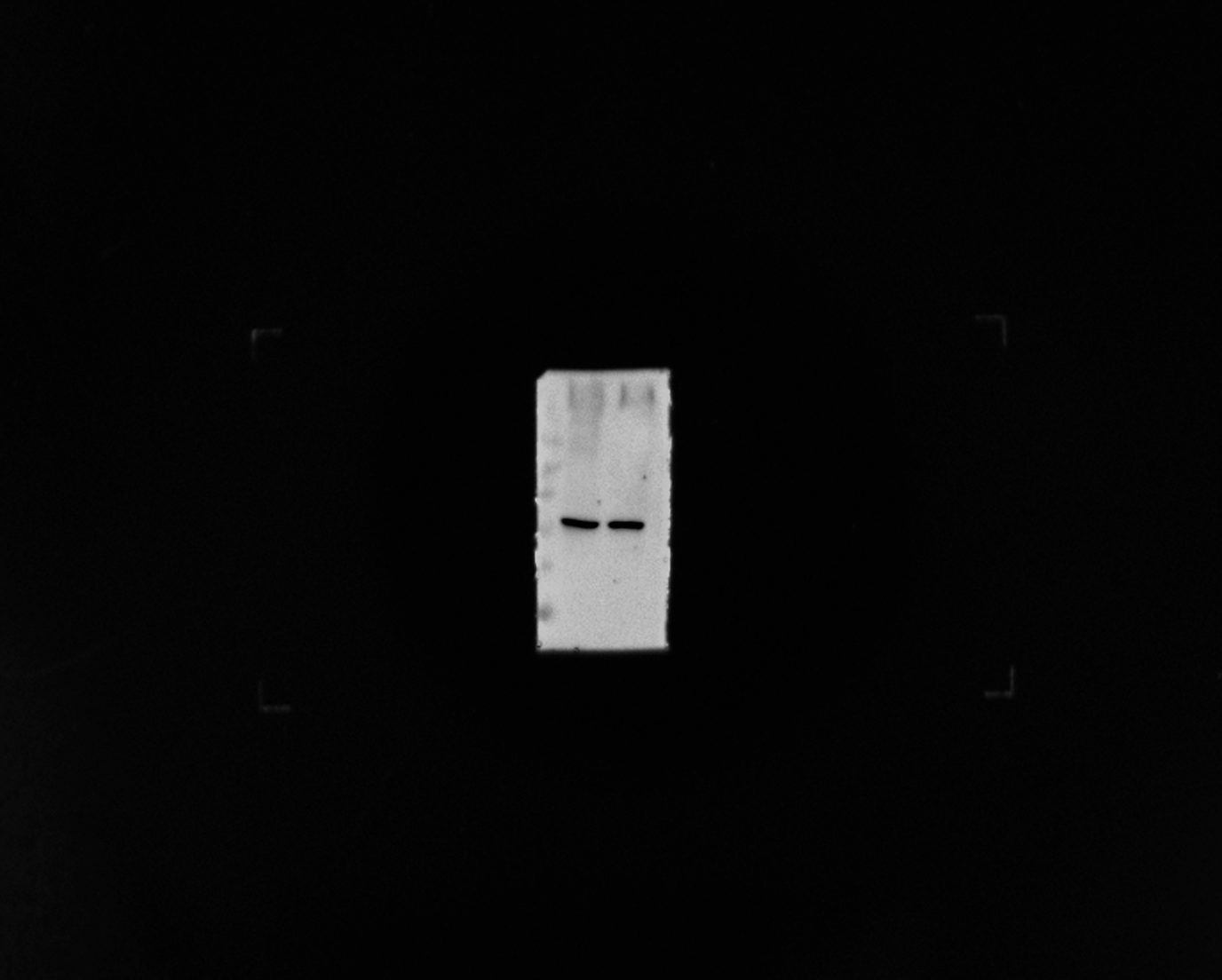


**BNLCL.2: BMI1**


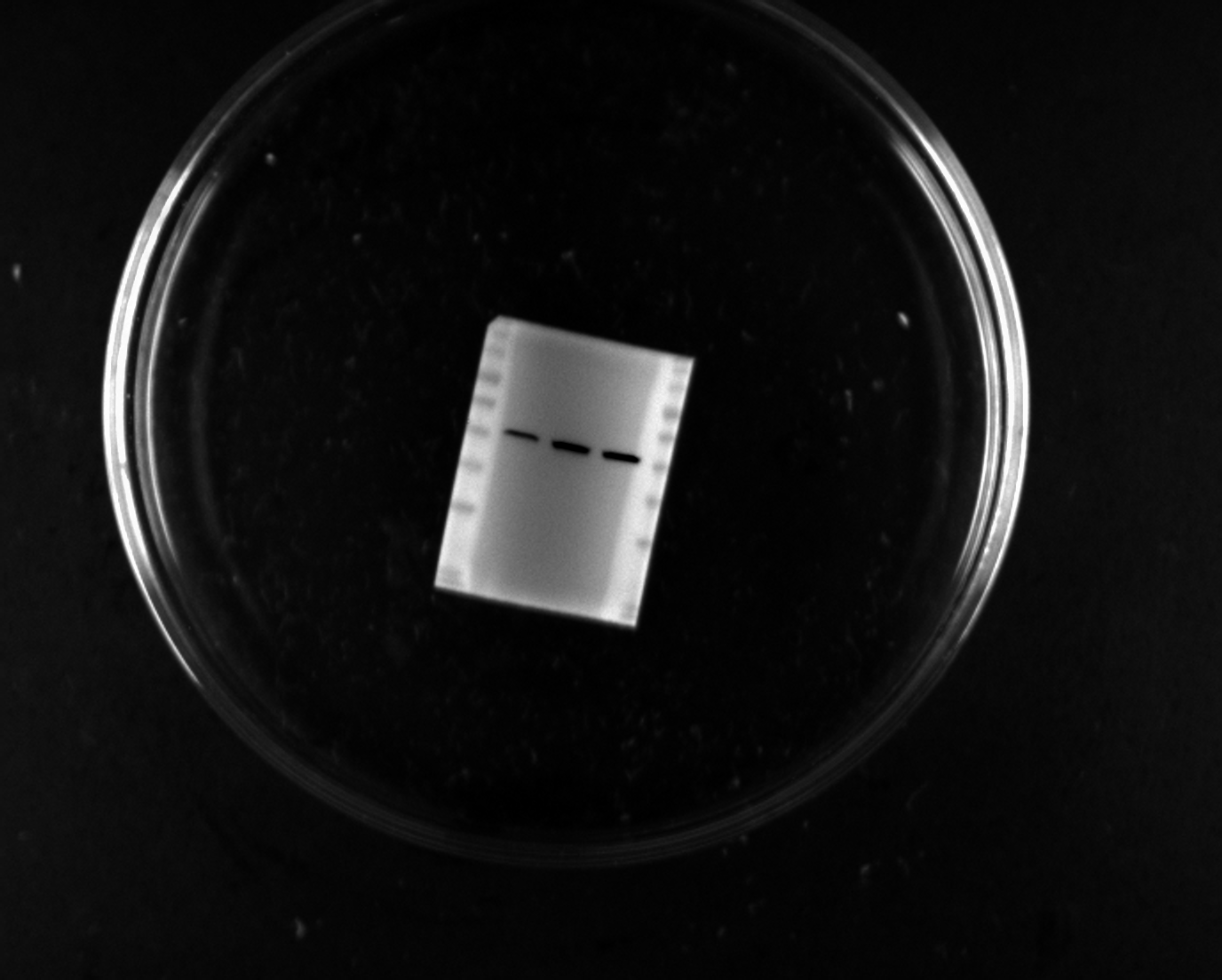

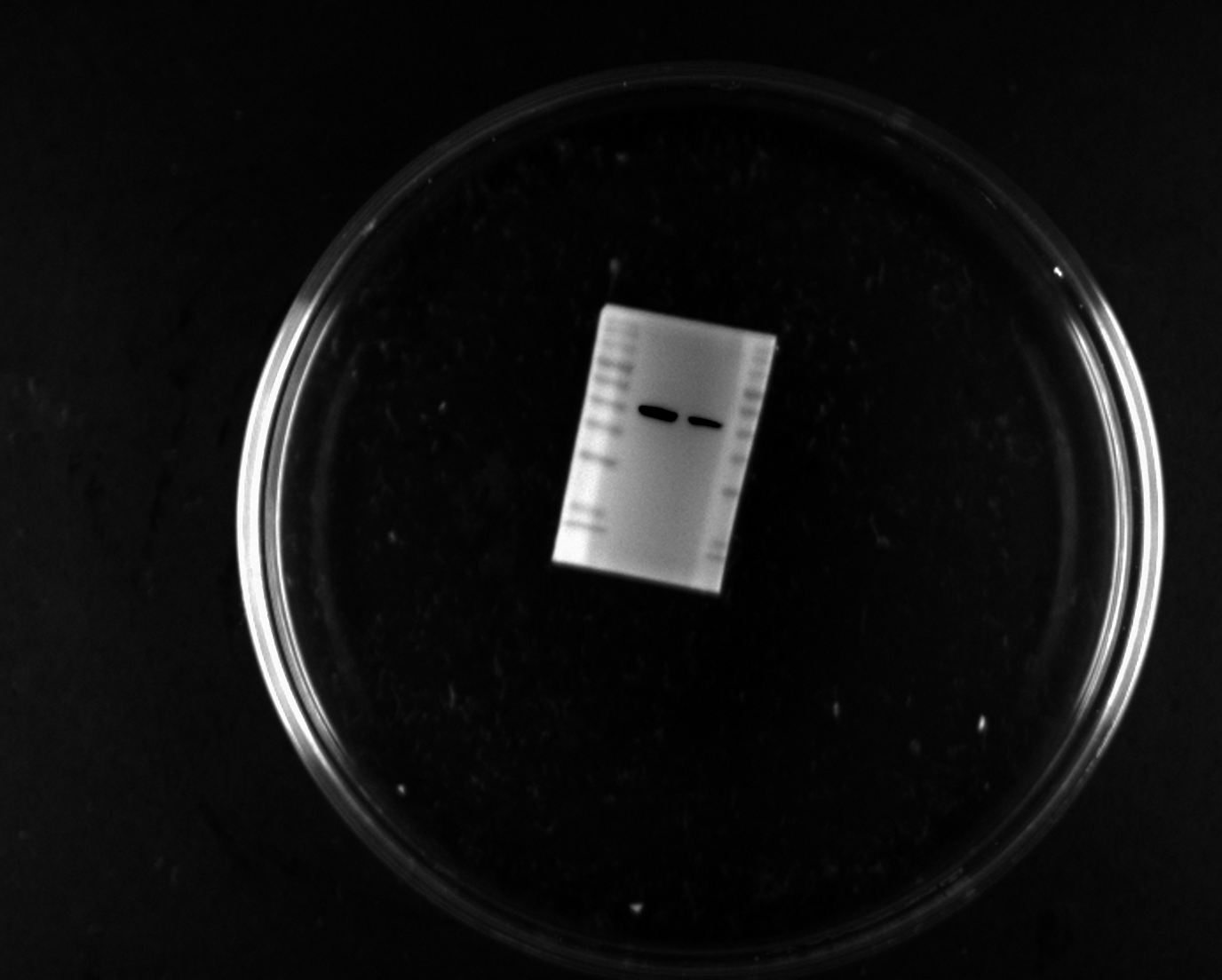


**BNLCL.2: β-actin**


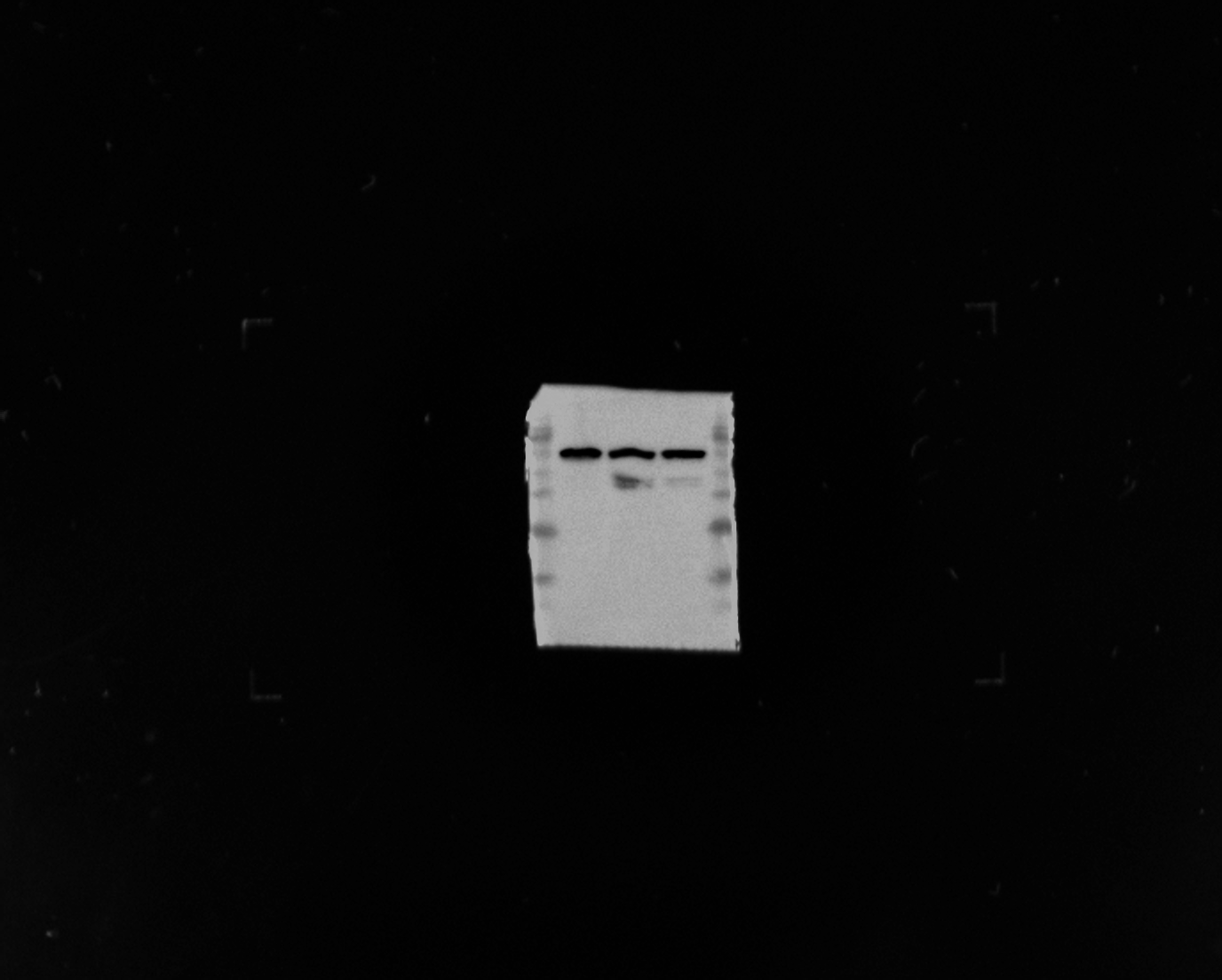

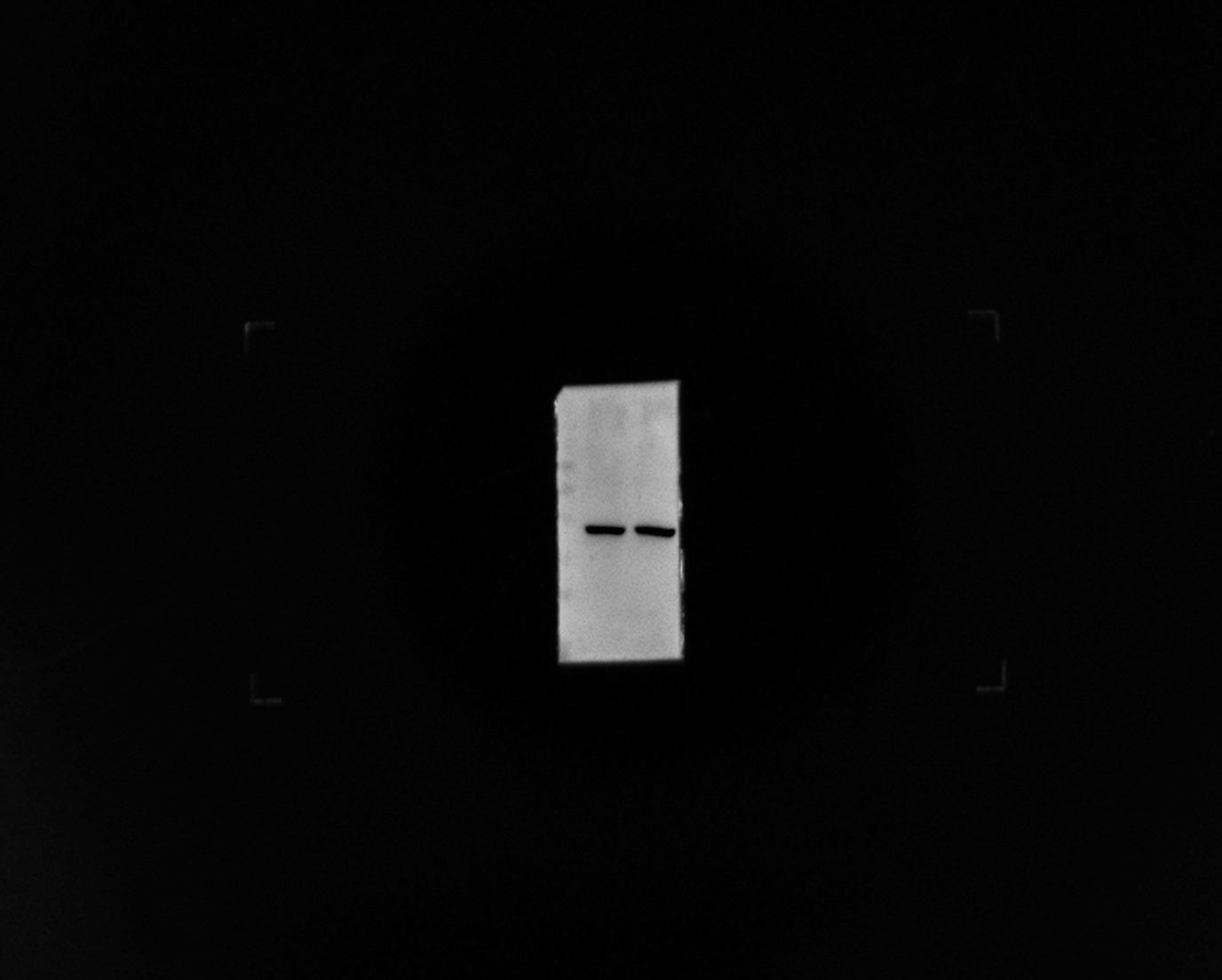


**Figure 2H**

**NCTC1469: BMI1**


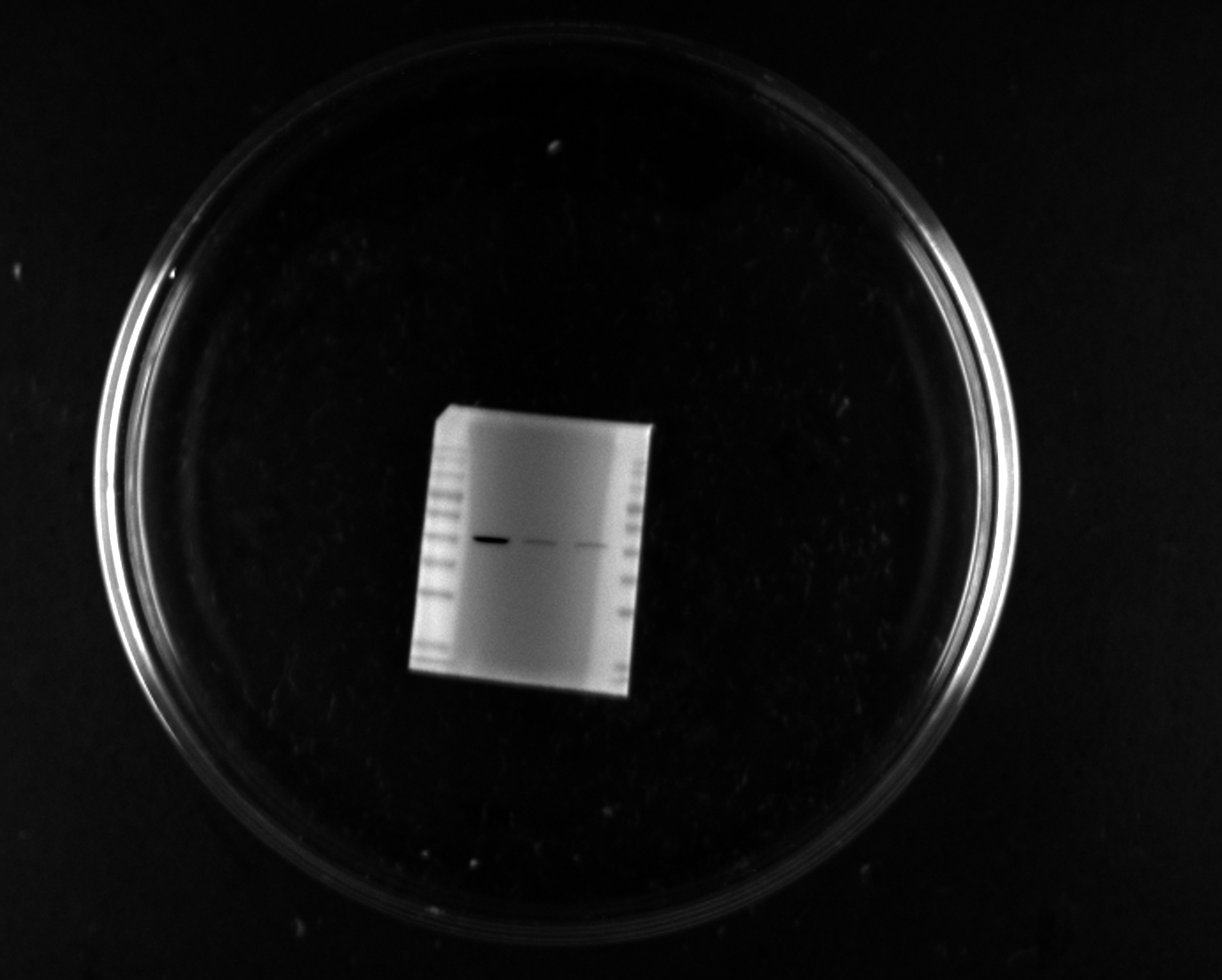

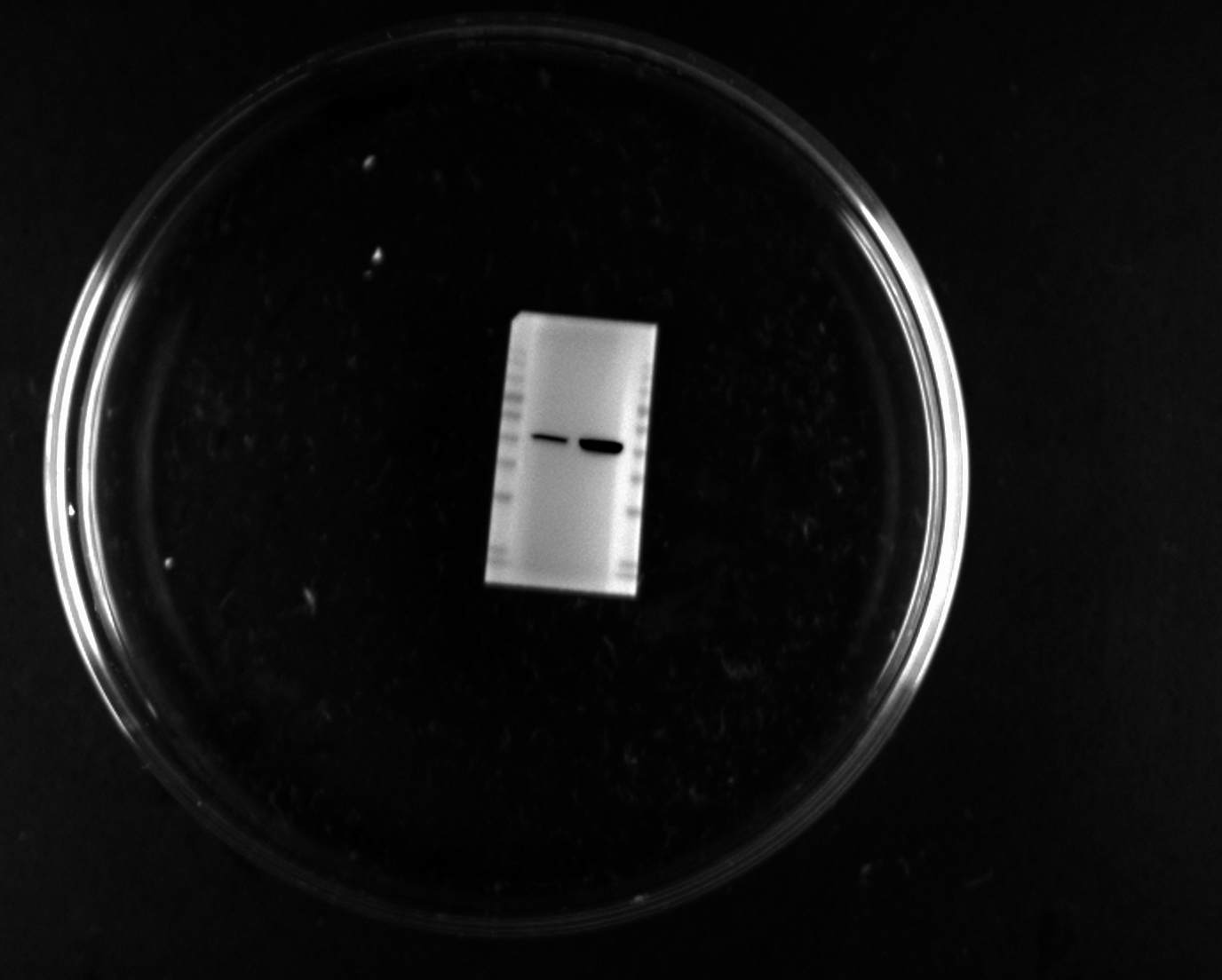


**NCTC1469: β-actin**


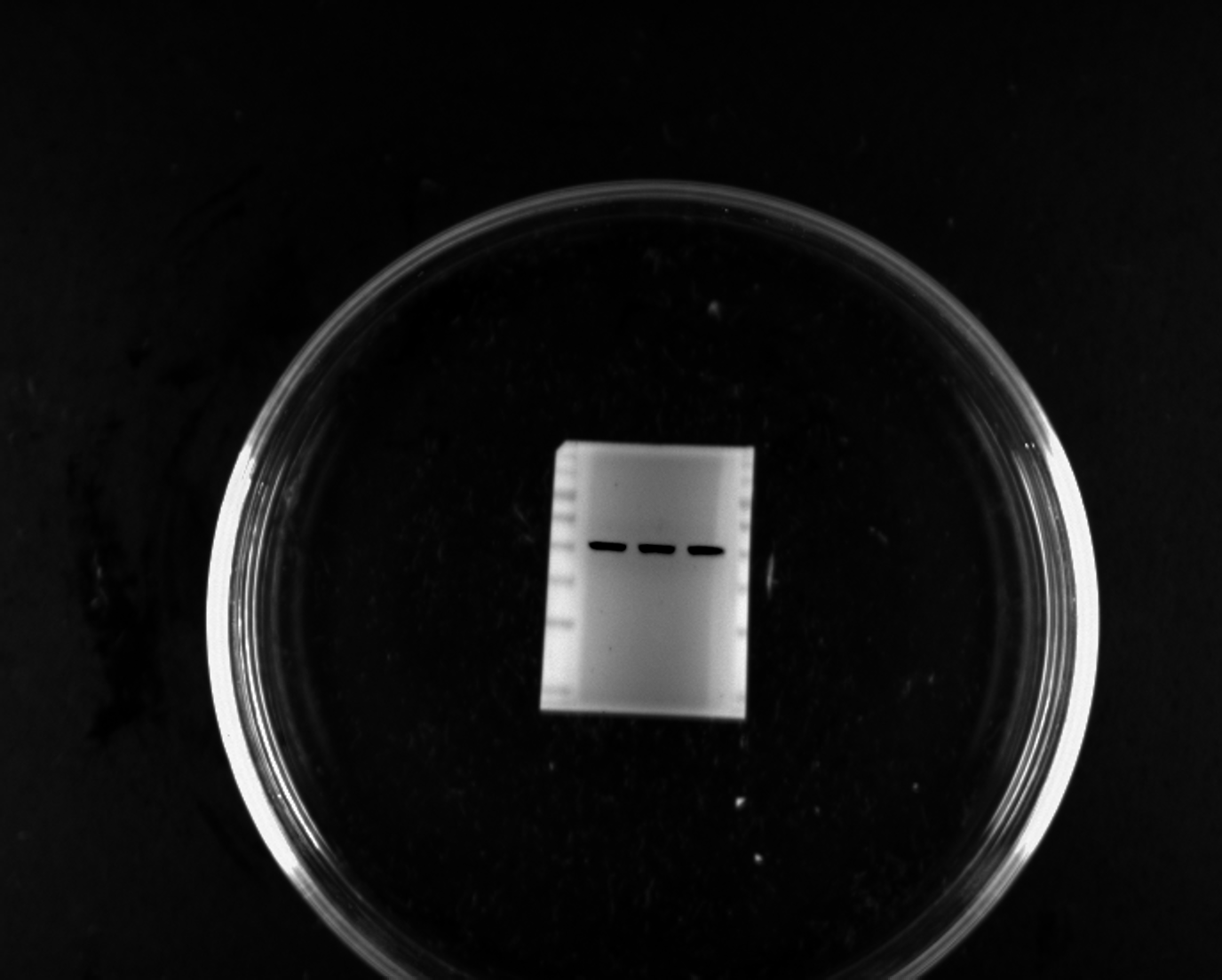

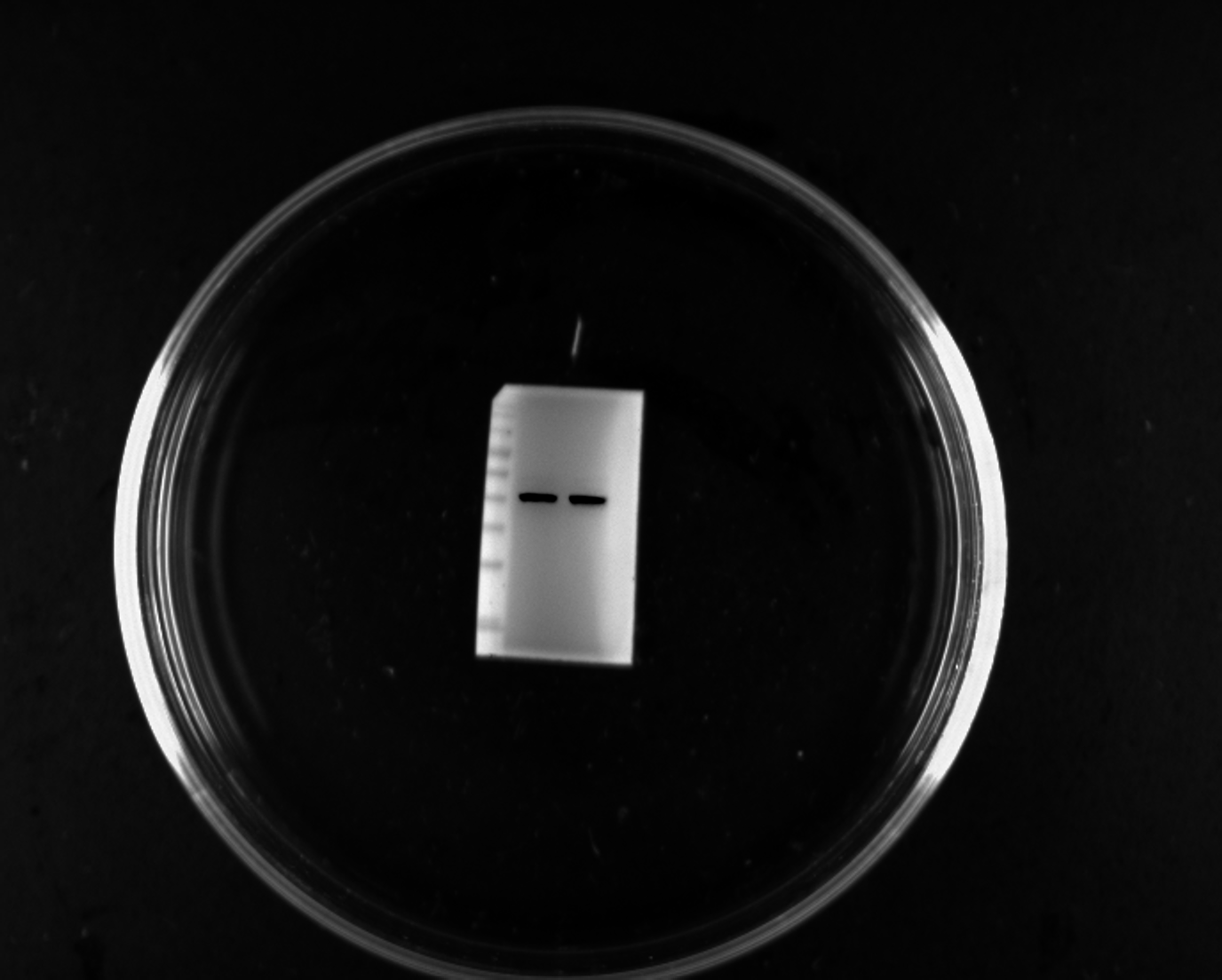


**BNLCL.2: BMI1**


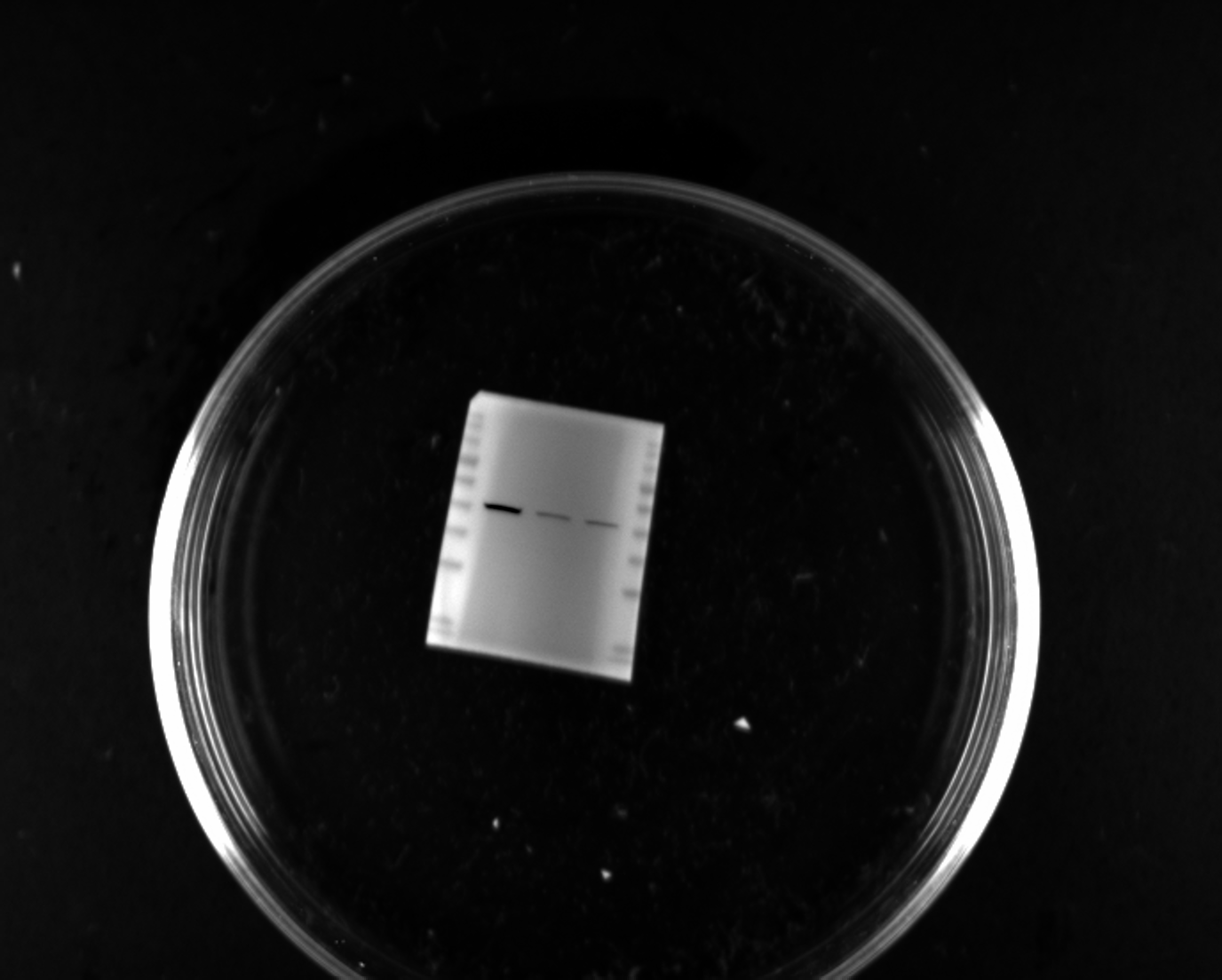

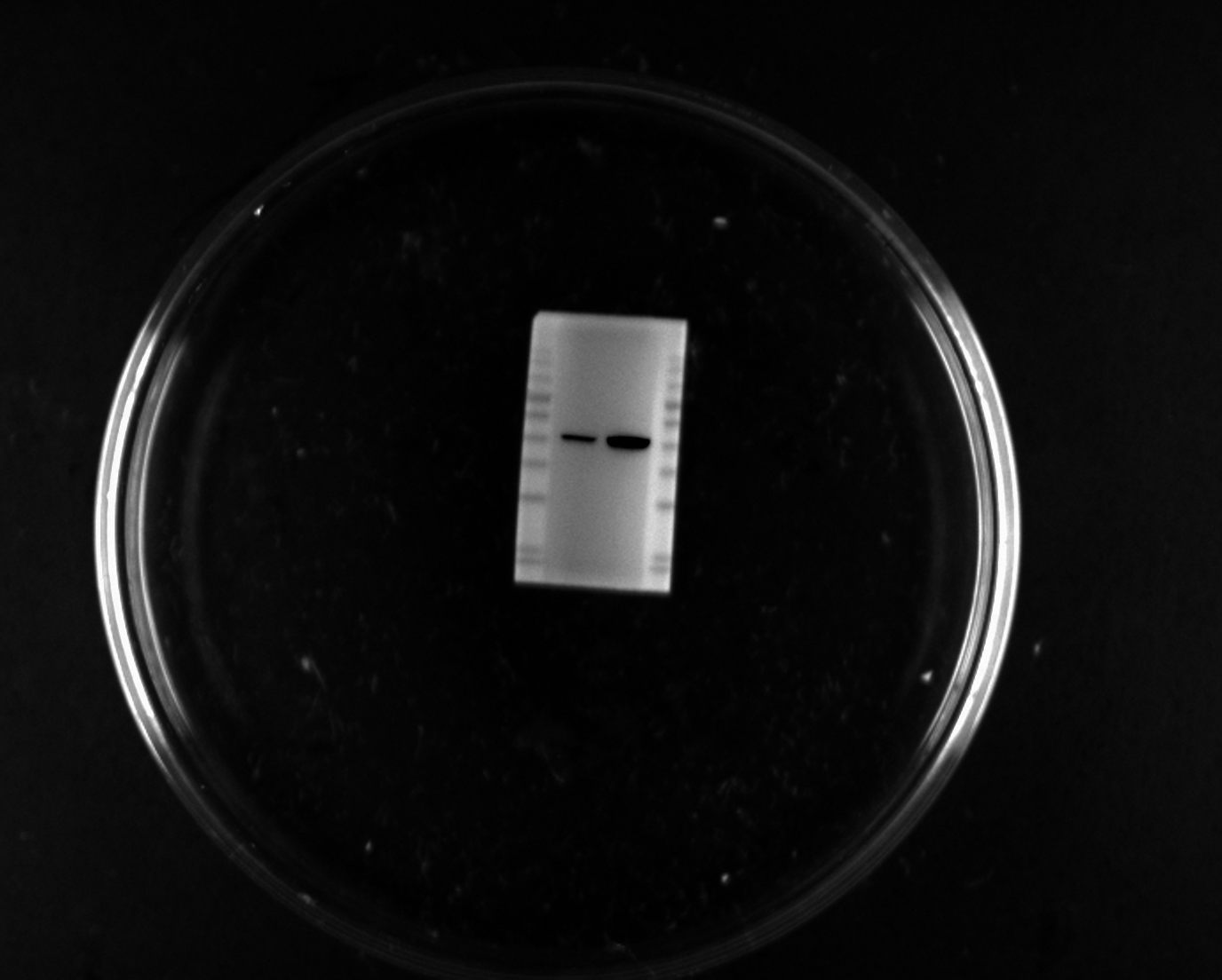


**BNLCL.2: β-actin**


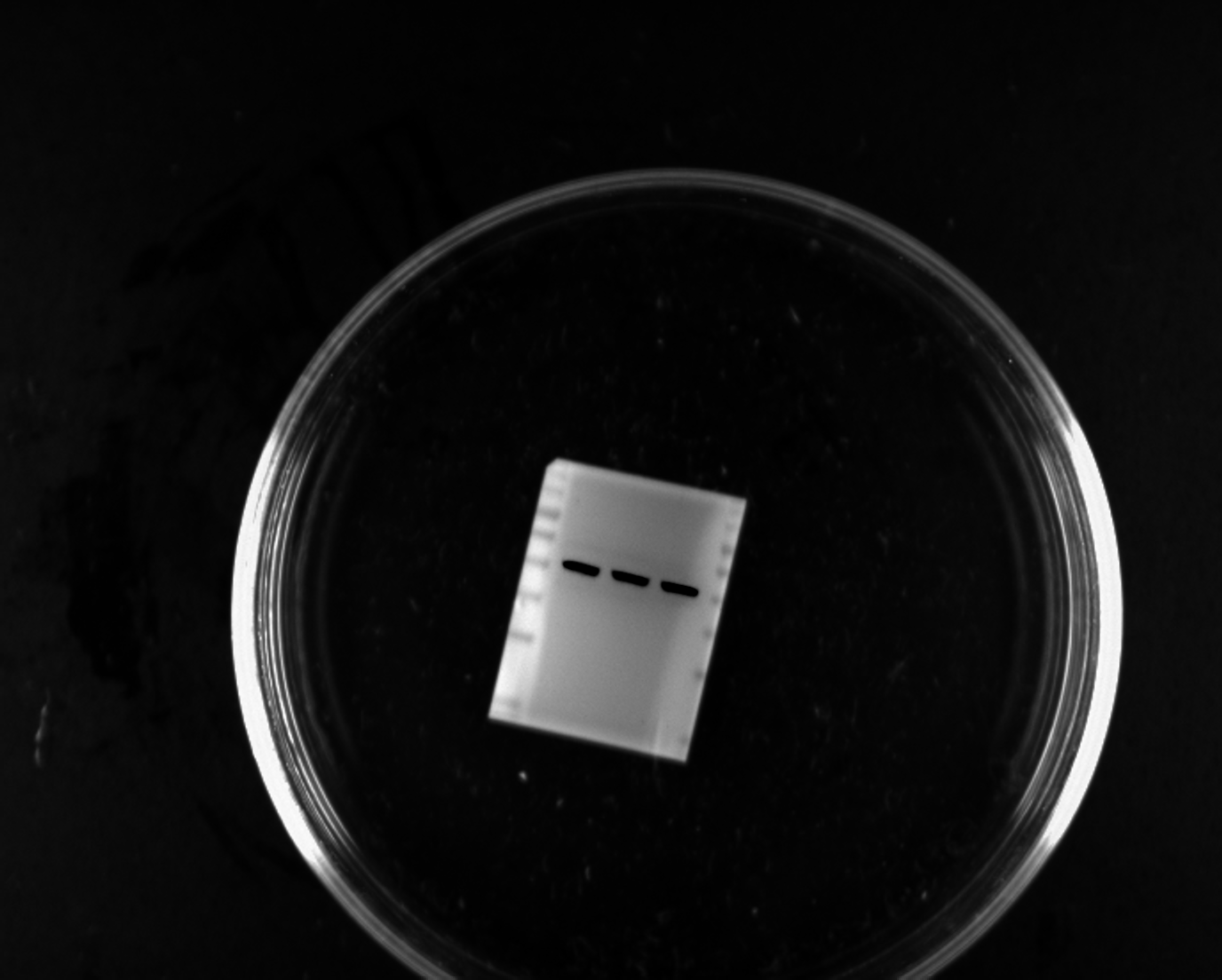

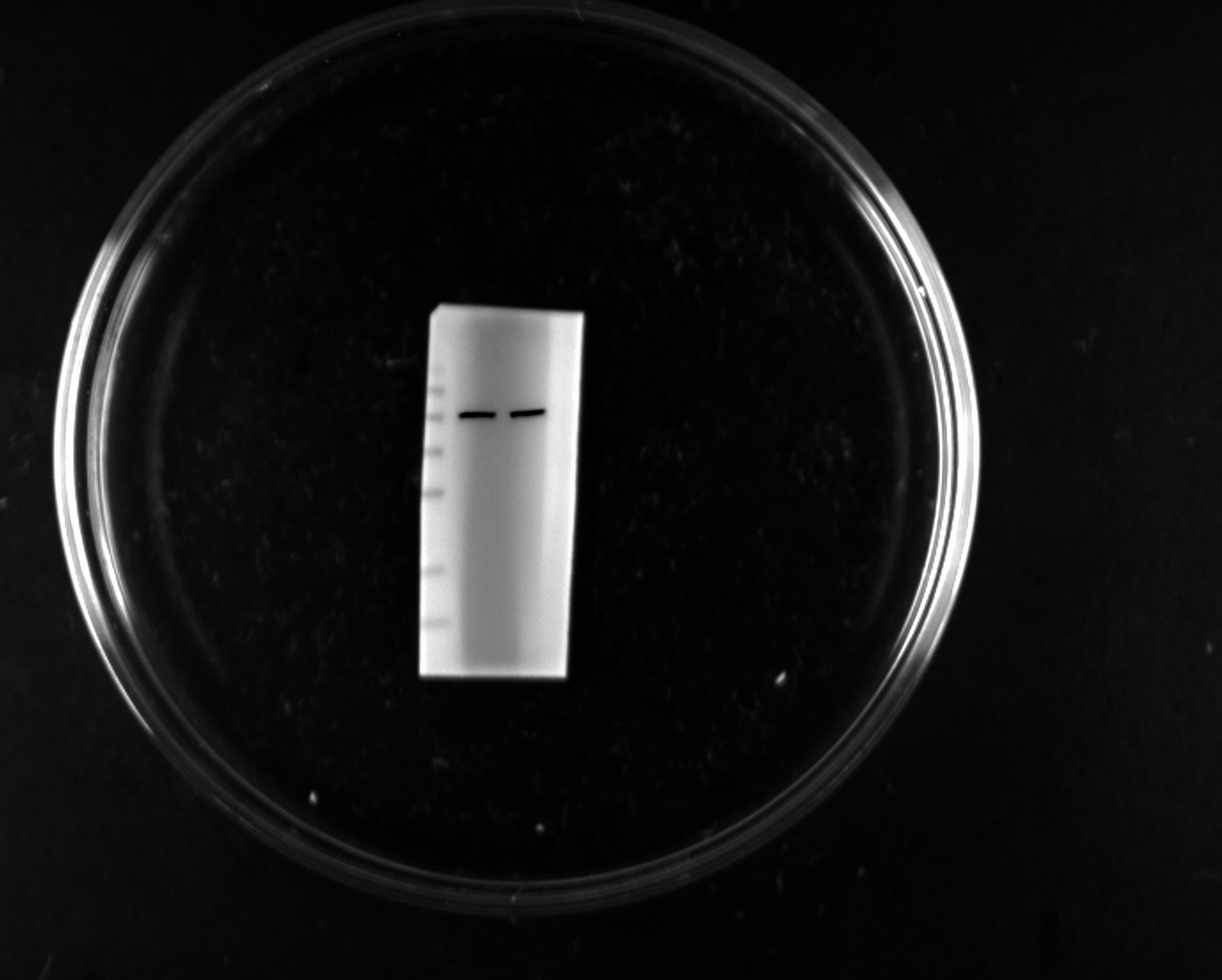


**Figure 2K**

**NCTC1469: CBX7**


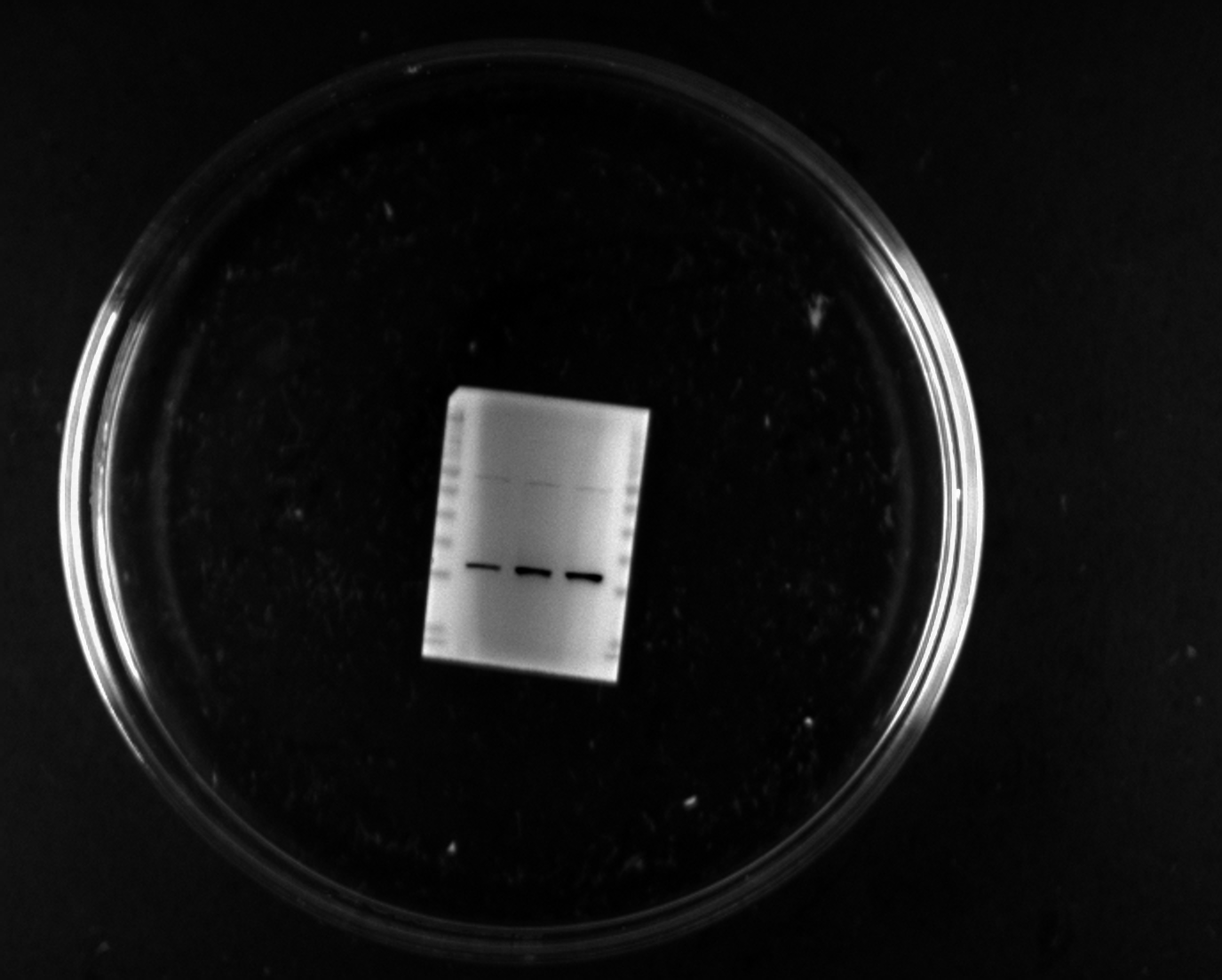

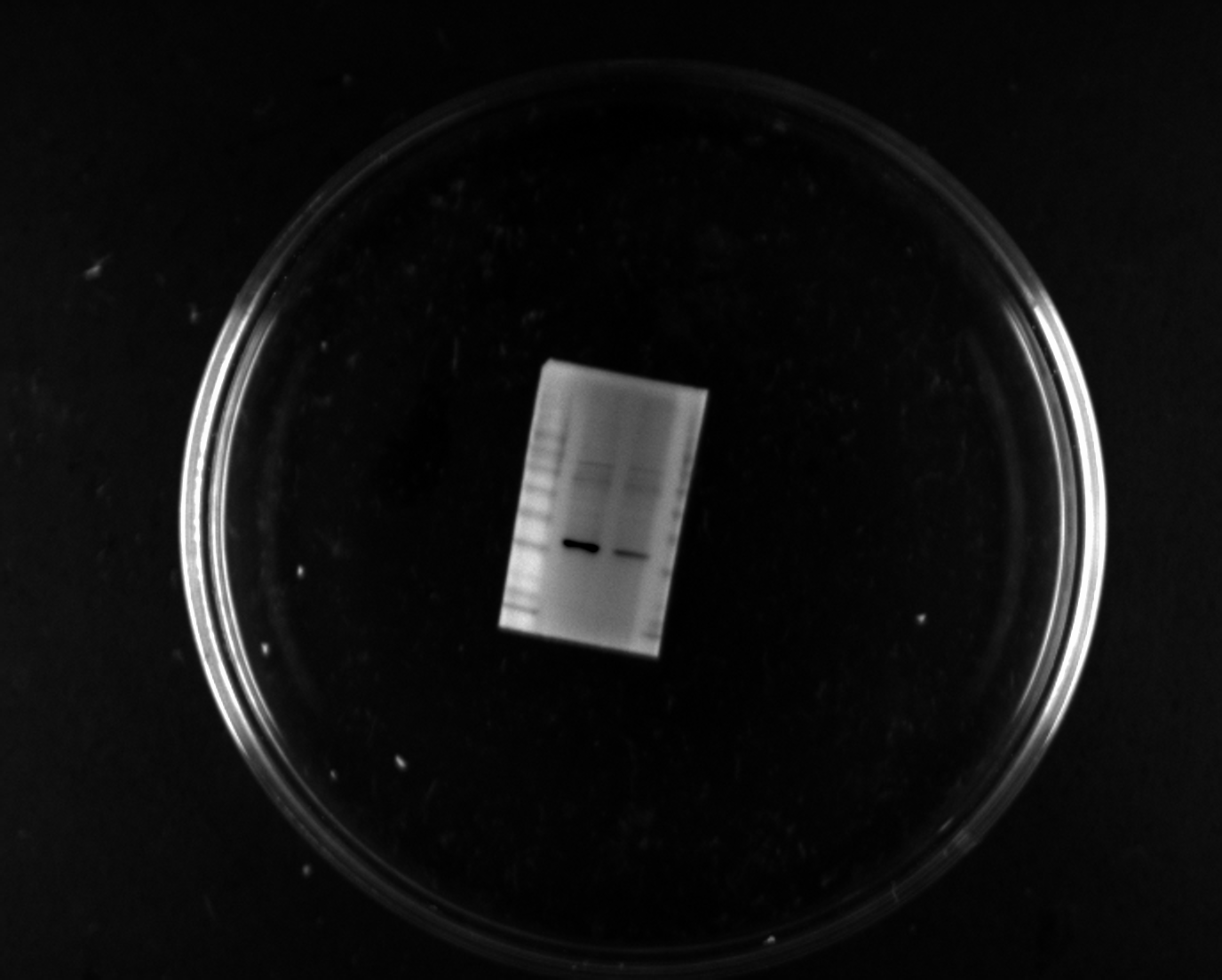


**NCTC1469: β-actin**


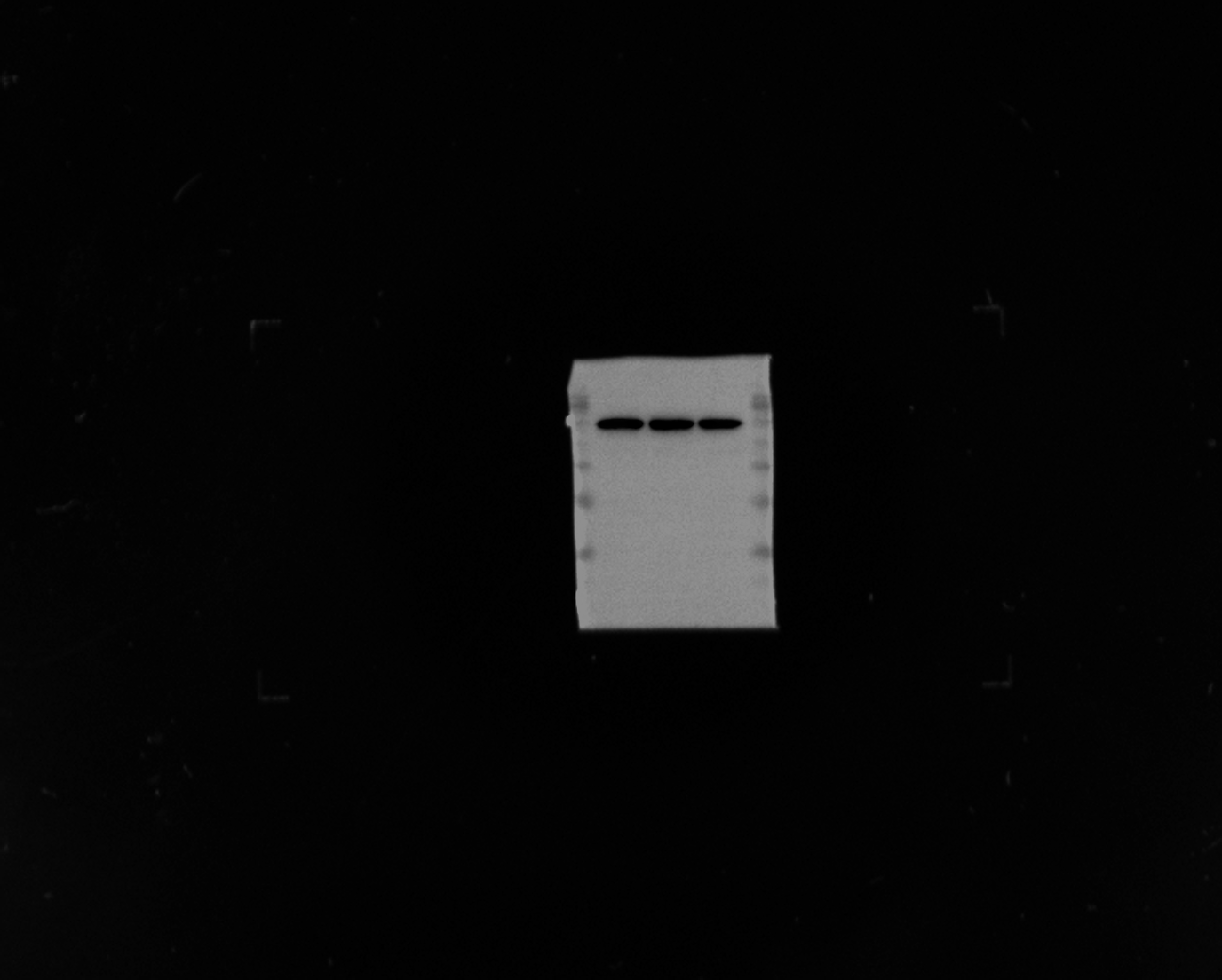

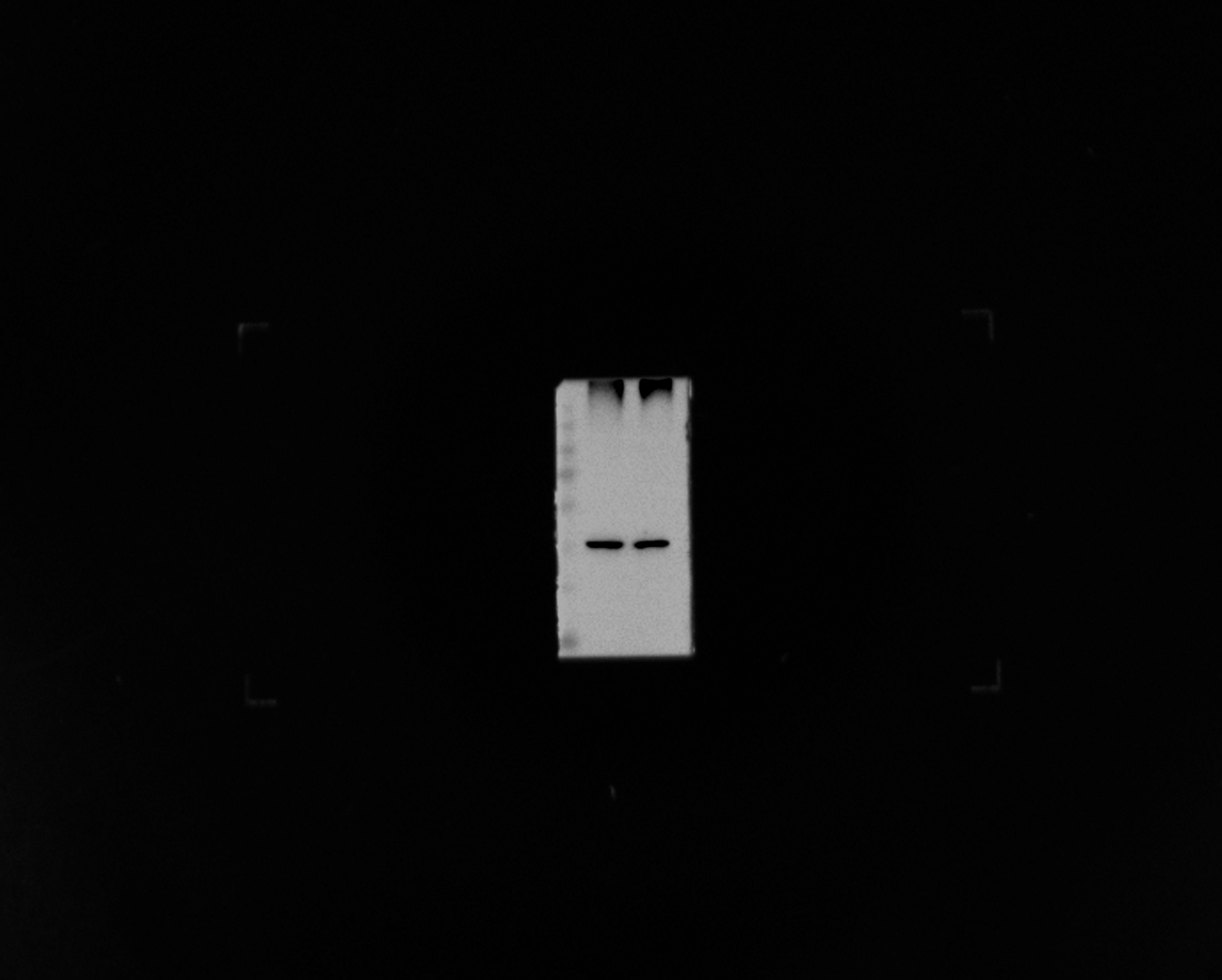


**BNLCL.2: CBX7**


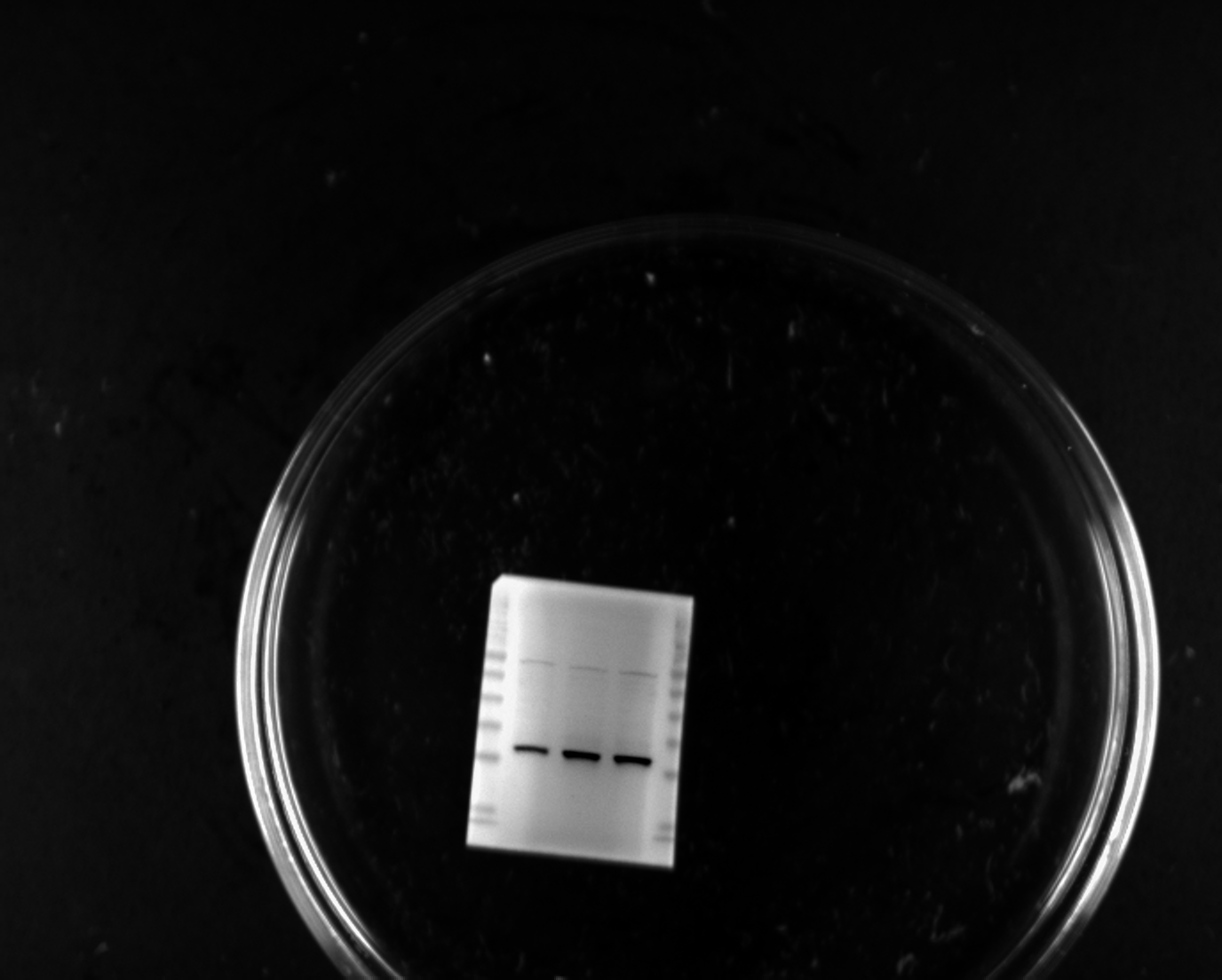

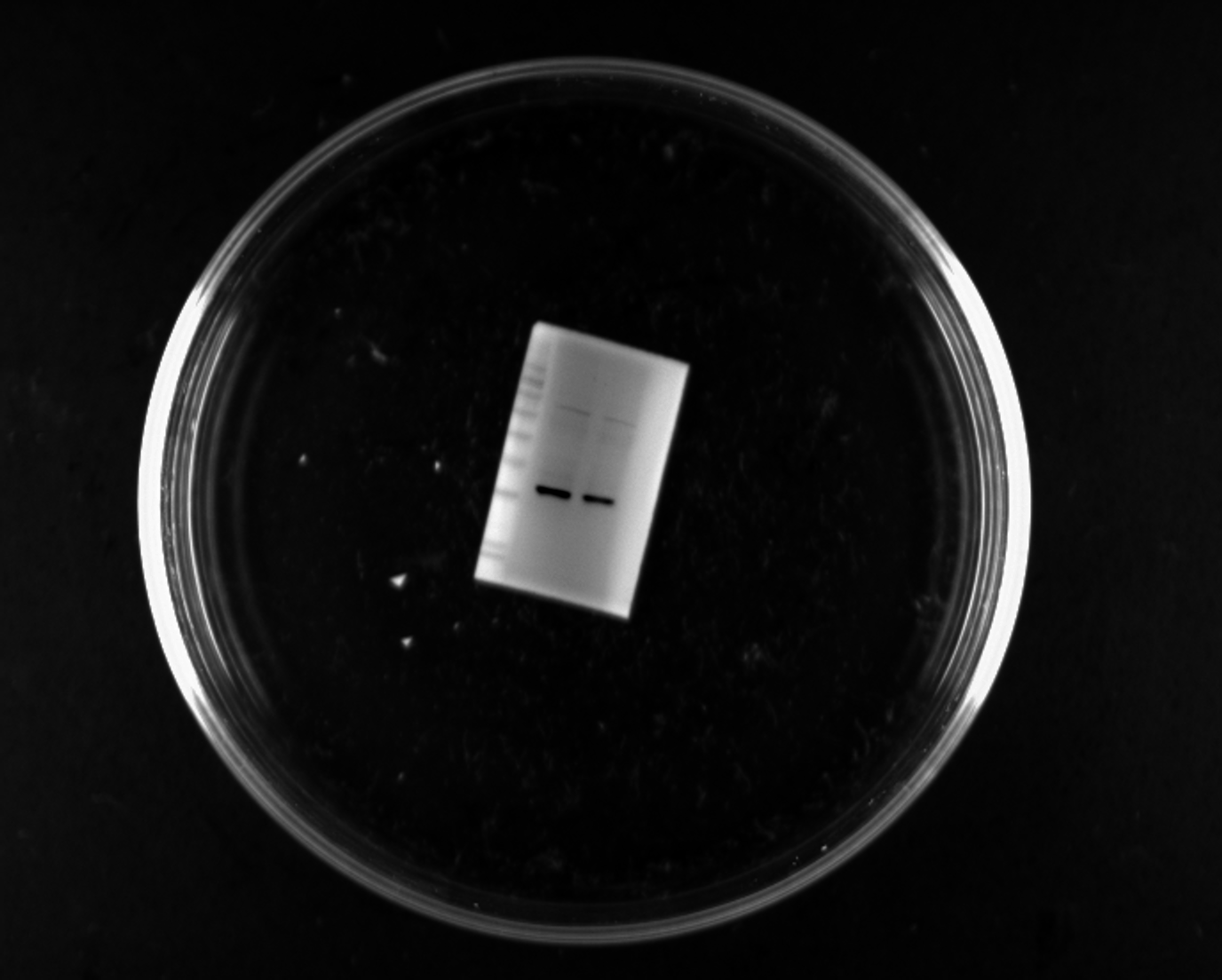


**BNLCL.2: β-actin**


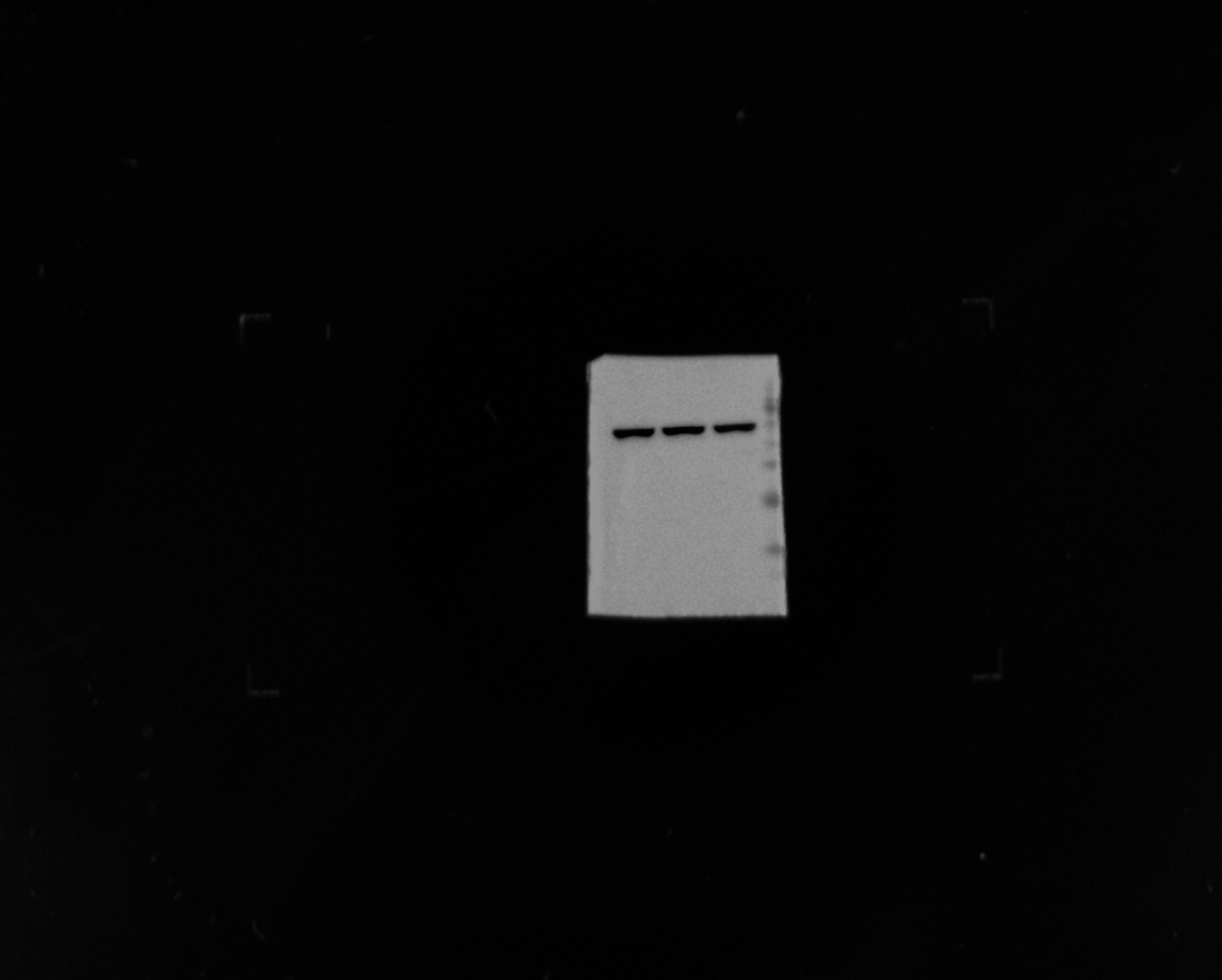

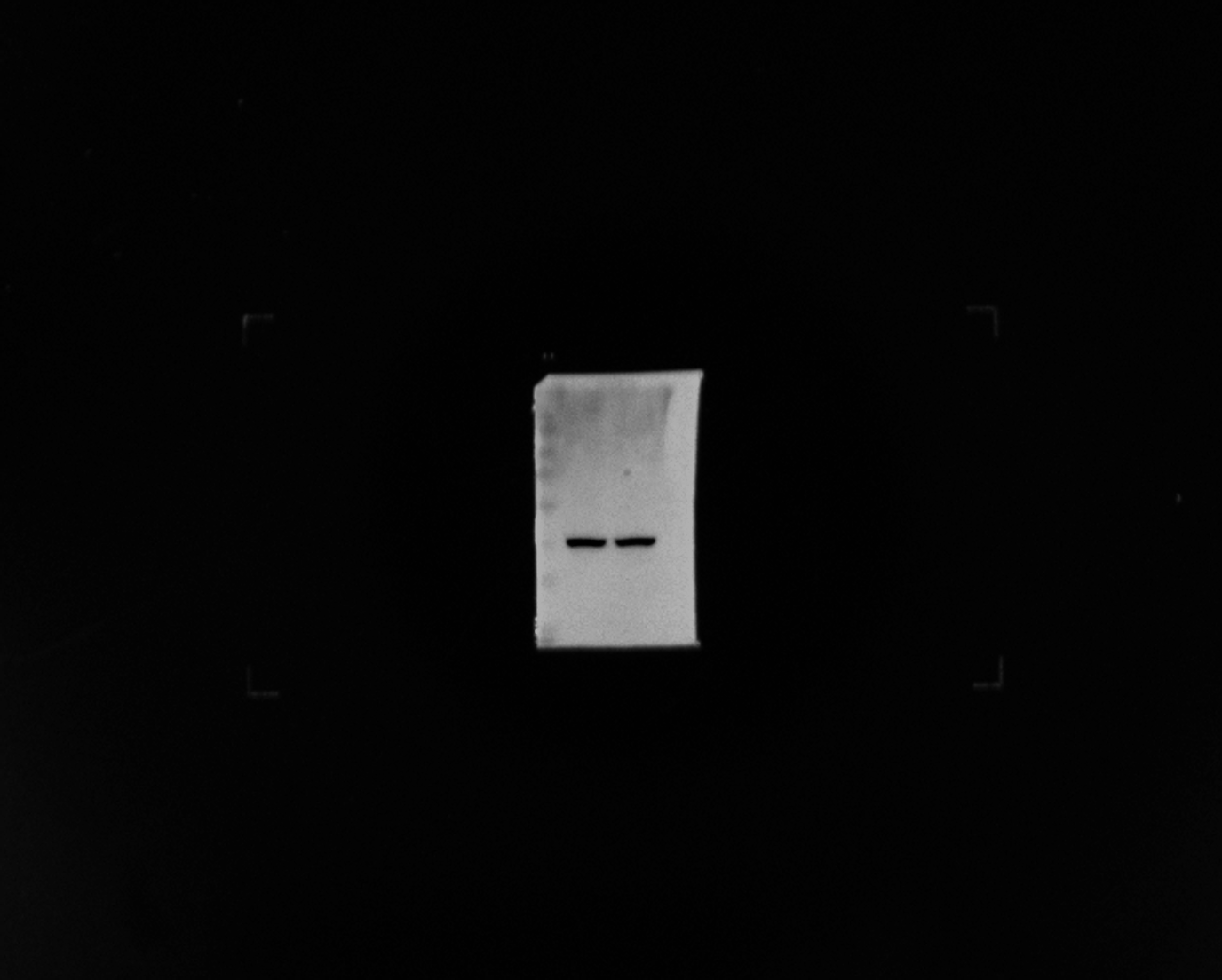


**Figure 3F**

**NCTC1469: CyclinD1**


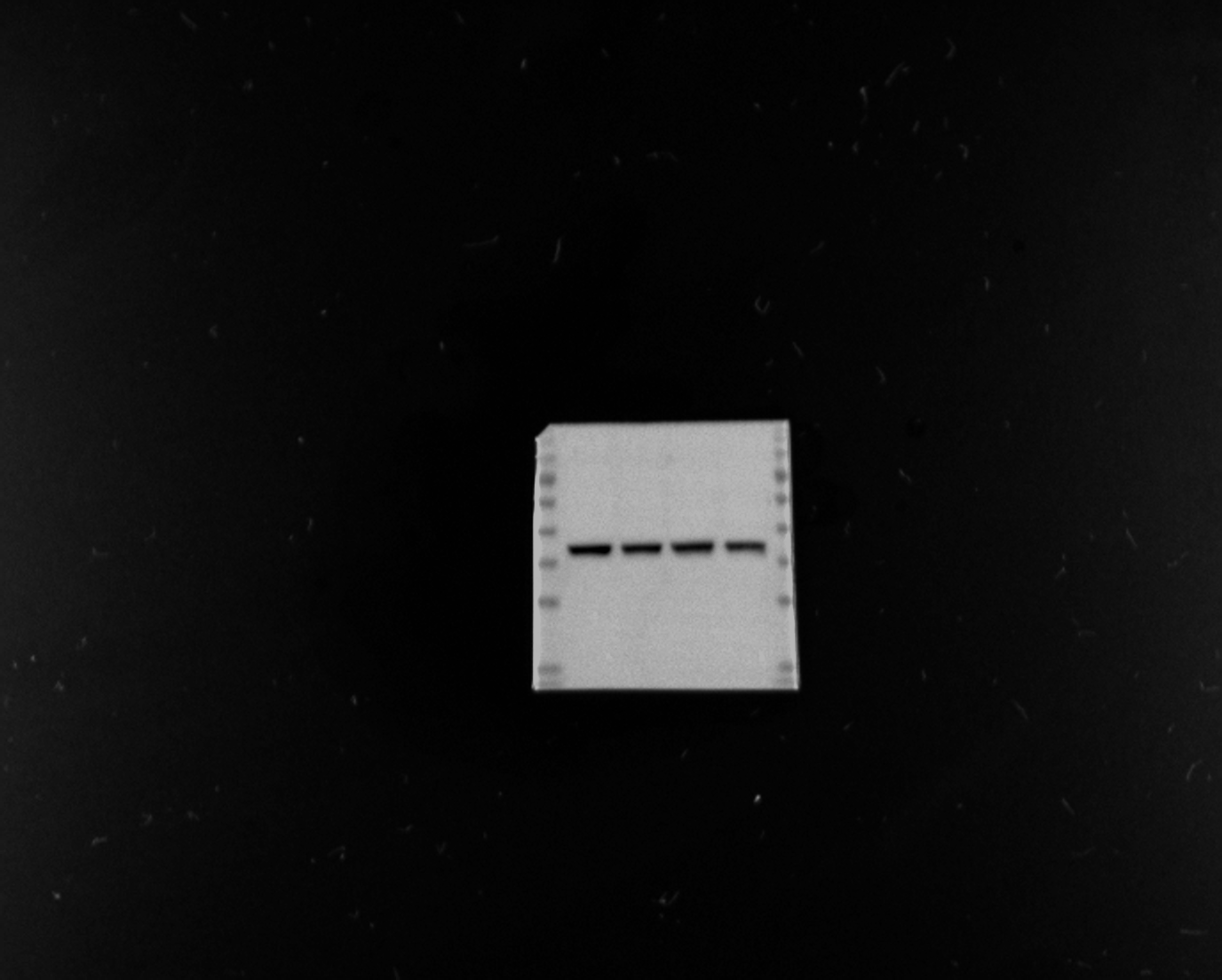


**NCTC1469: CyclinE**


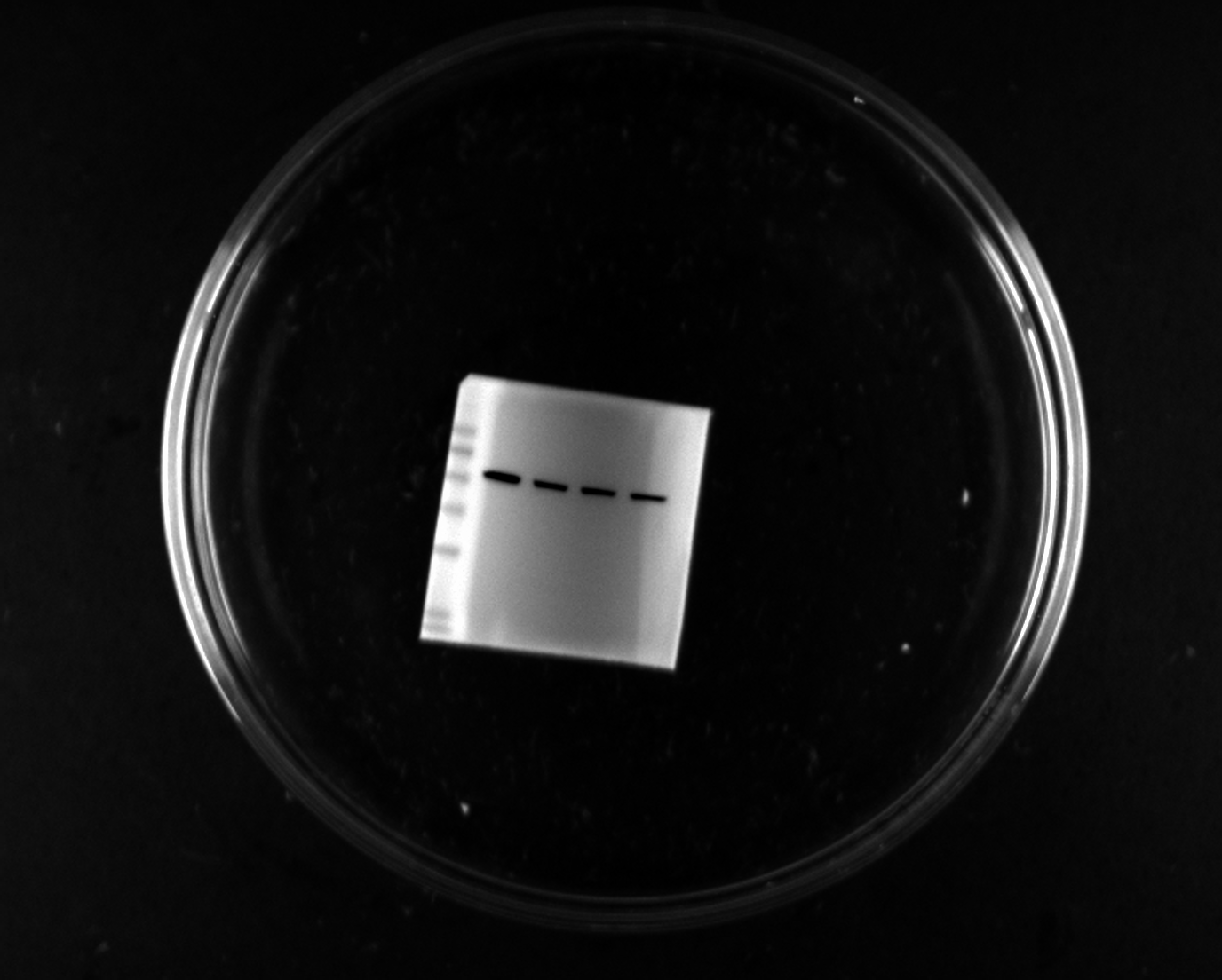


**NCTC1469: β-actin**


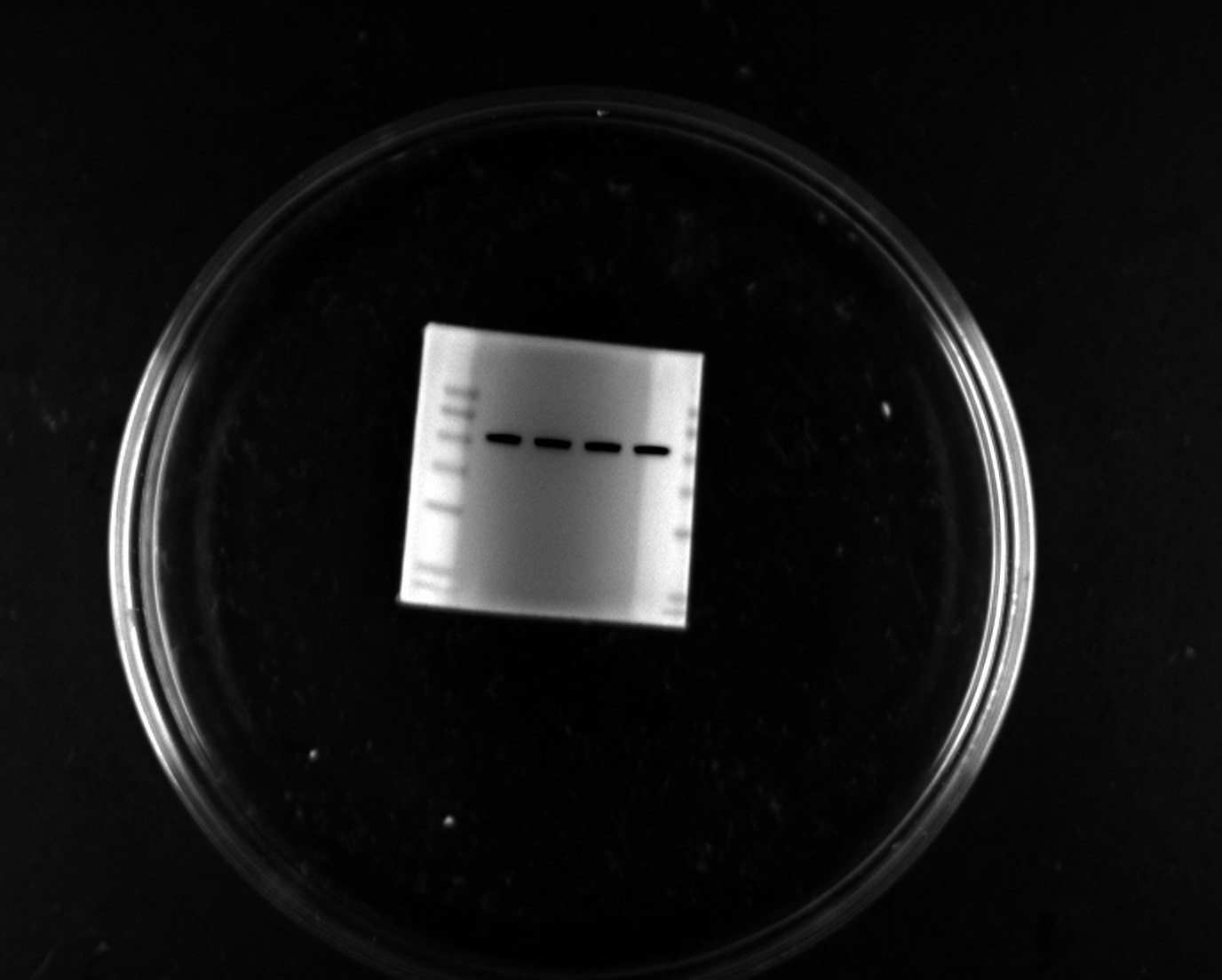


**BNLCL.2: CyclinD1**


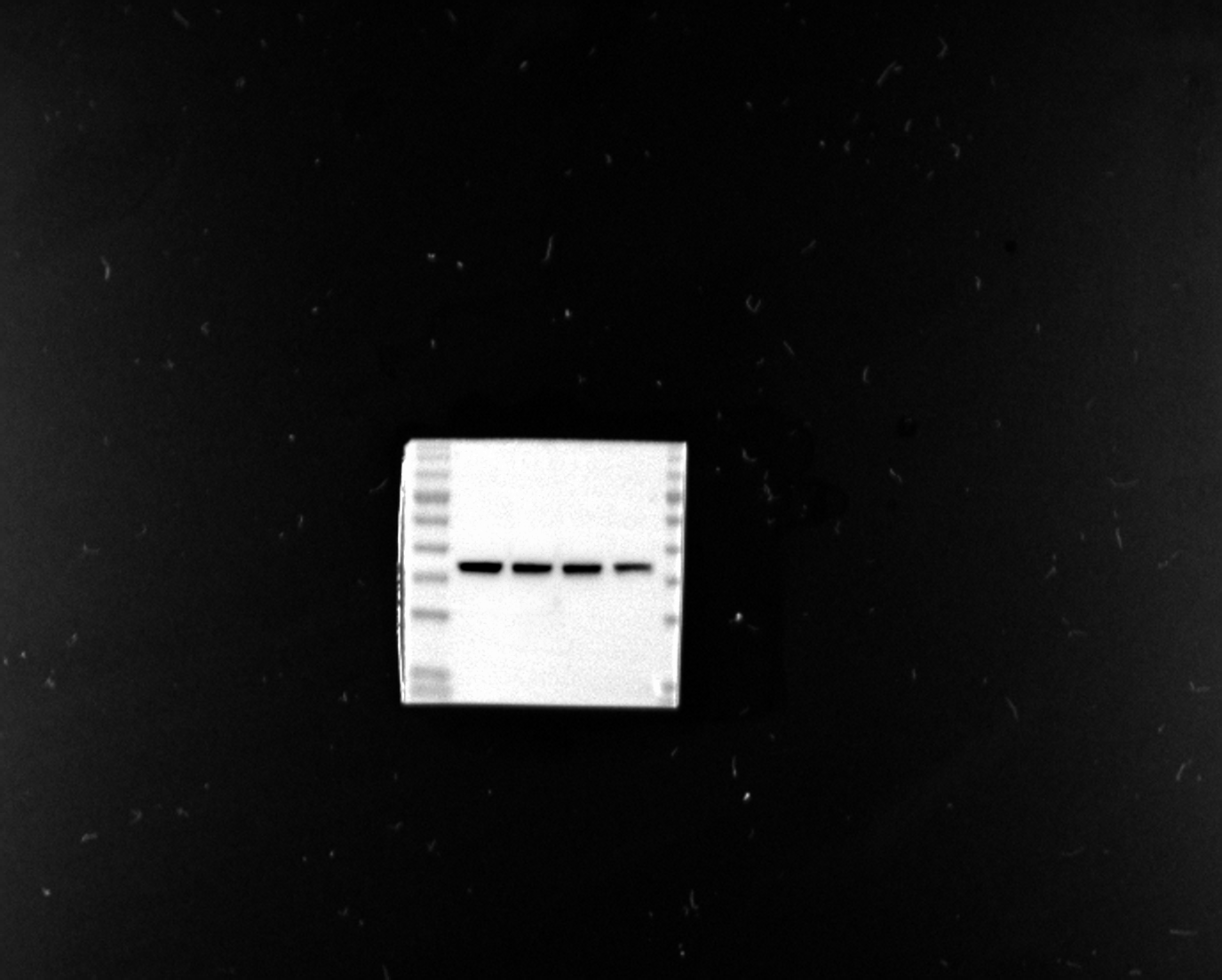


**BNLCL.2: CyclinE**


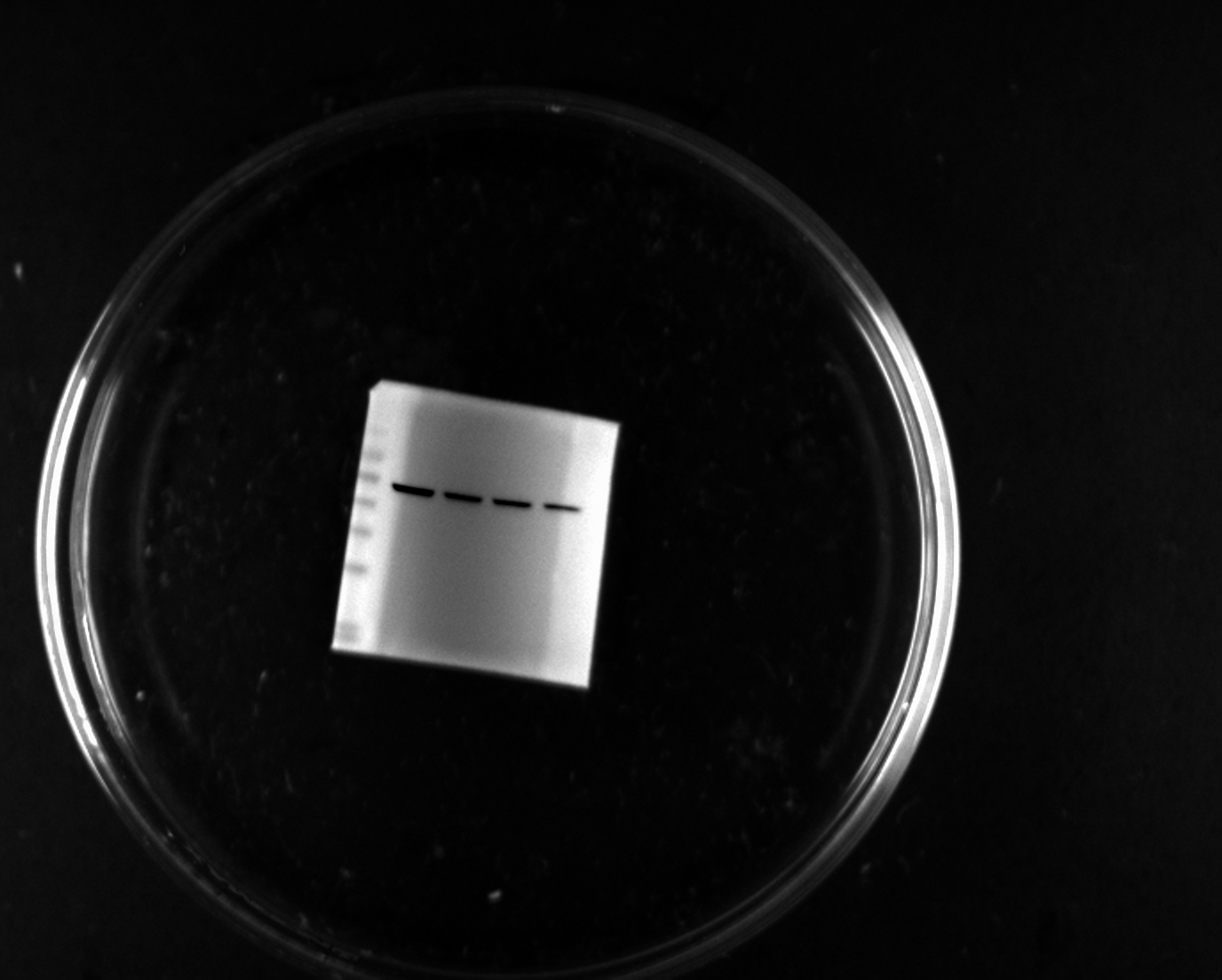


**BNLCL.2: β-actin**


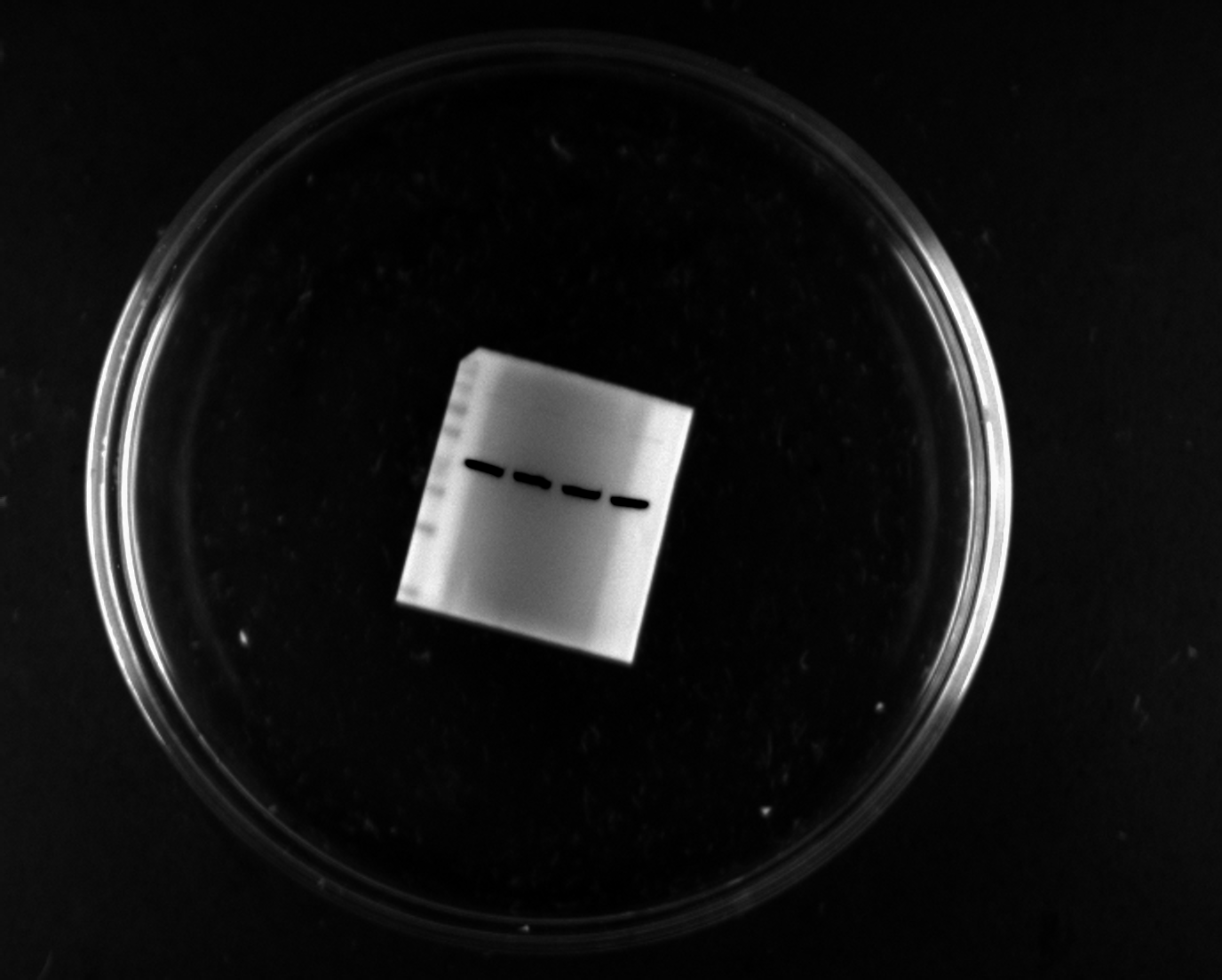


**Figure 4C**

**NCTC1469: Nuclear Nrf2**


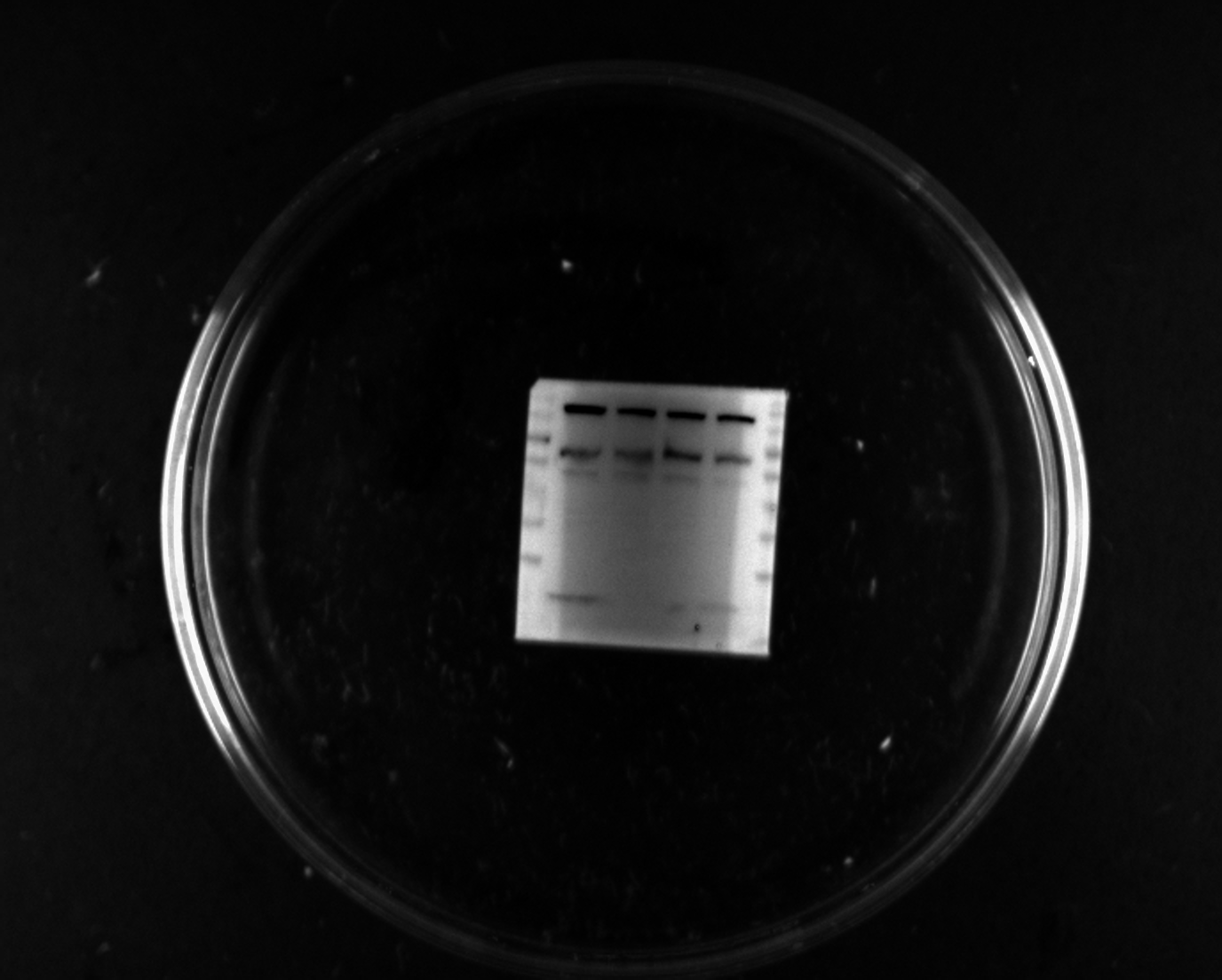


**NCTC1469: LaminB**


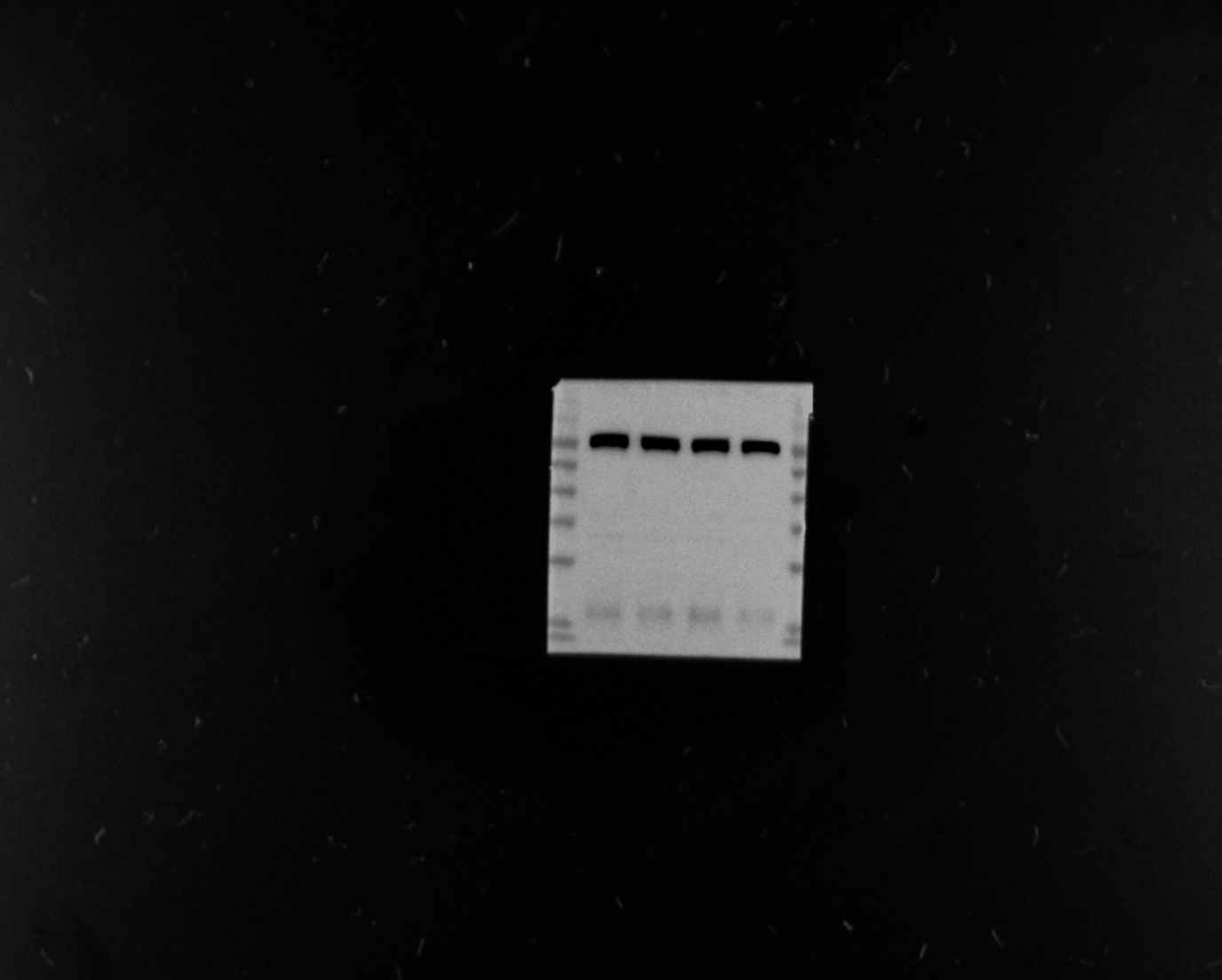


**NCTC1469: Nrf2**


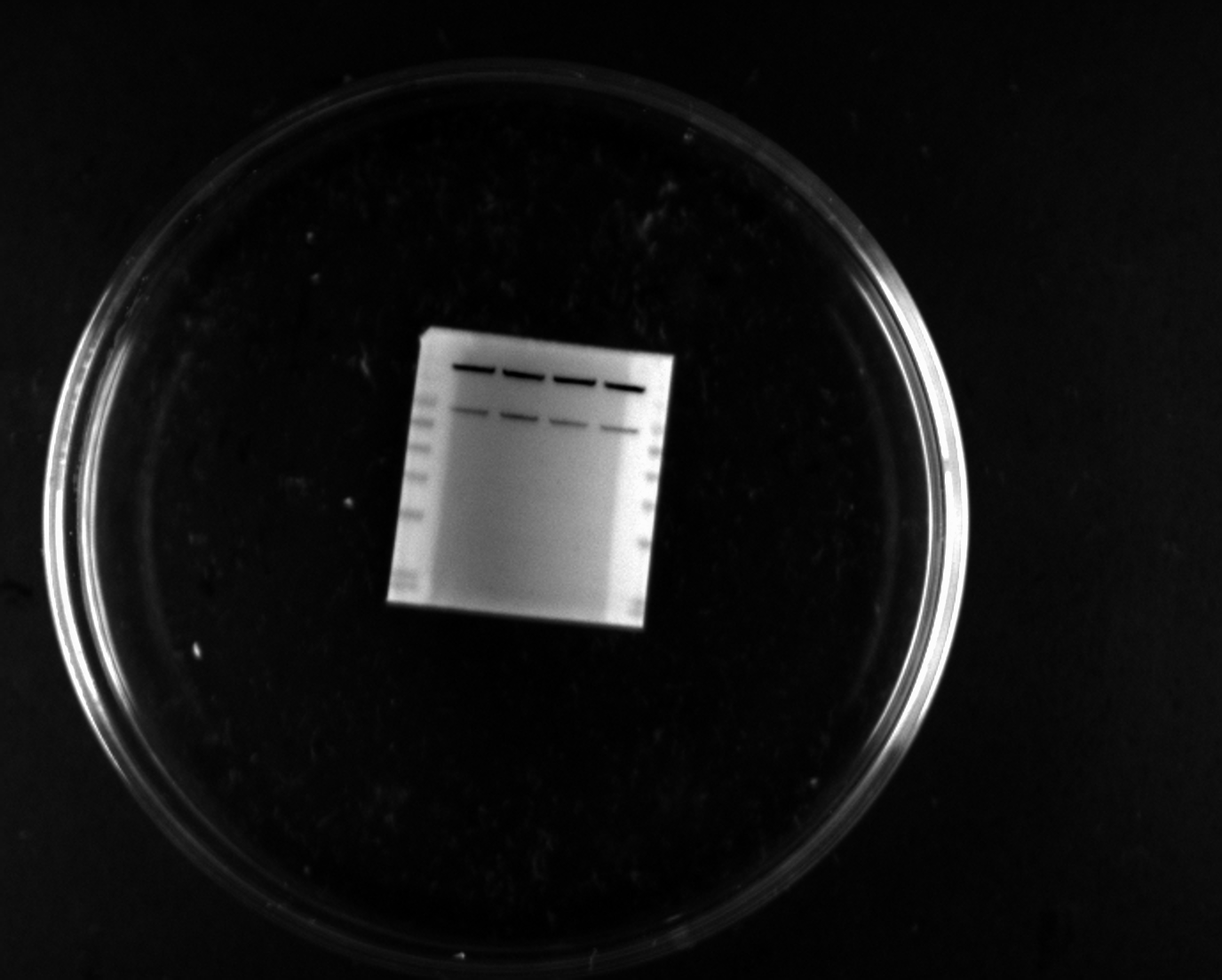


**NCTC1469: HO-1**


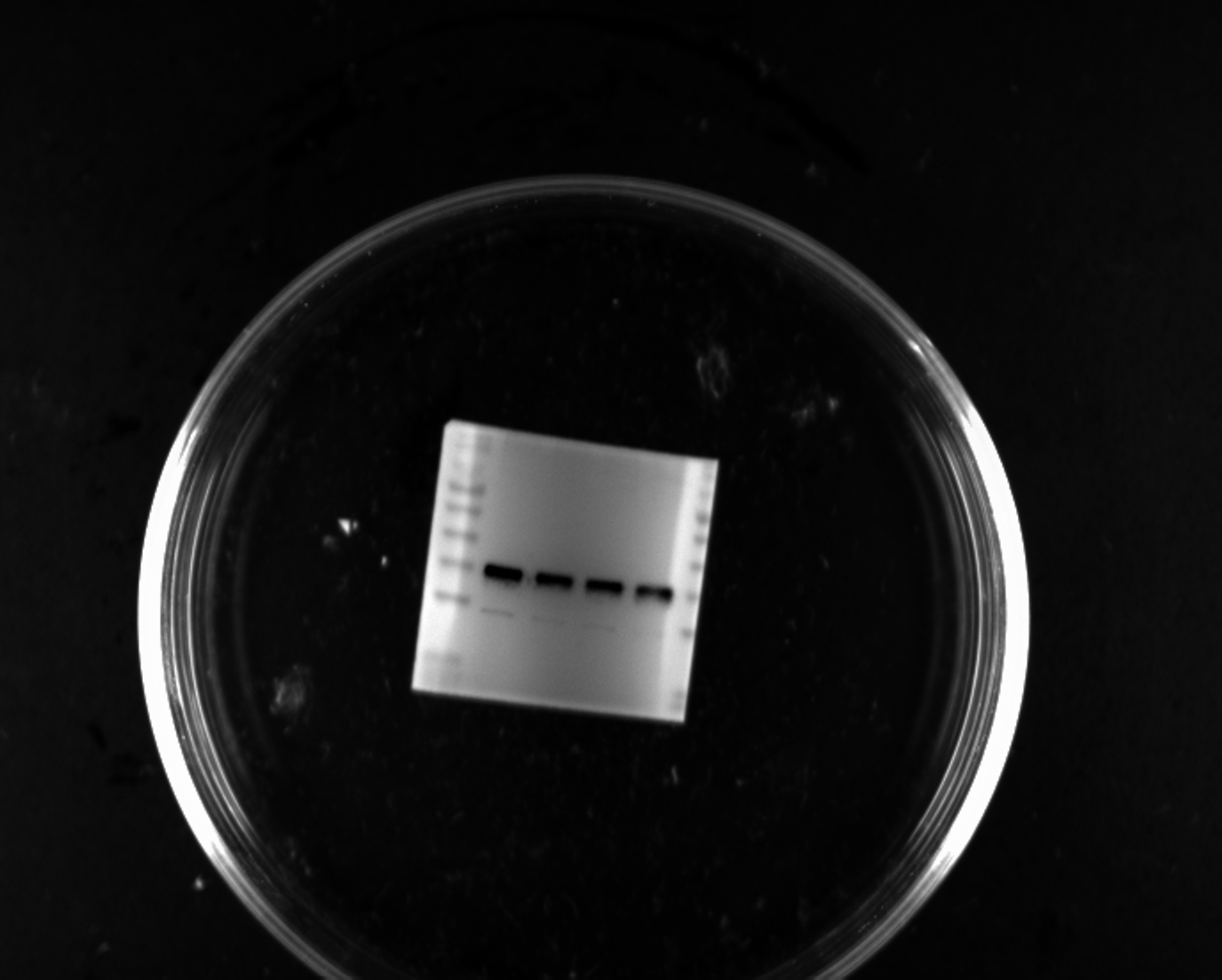


**NCTC1469: NQO-1**


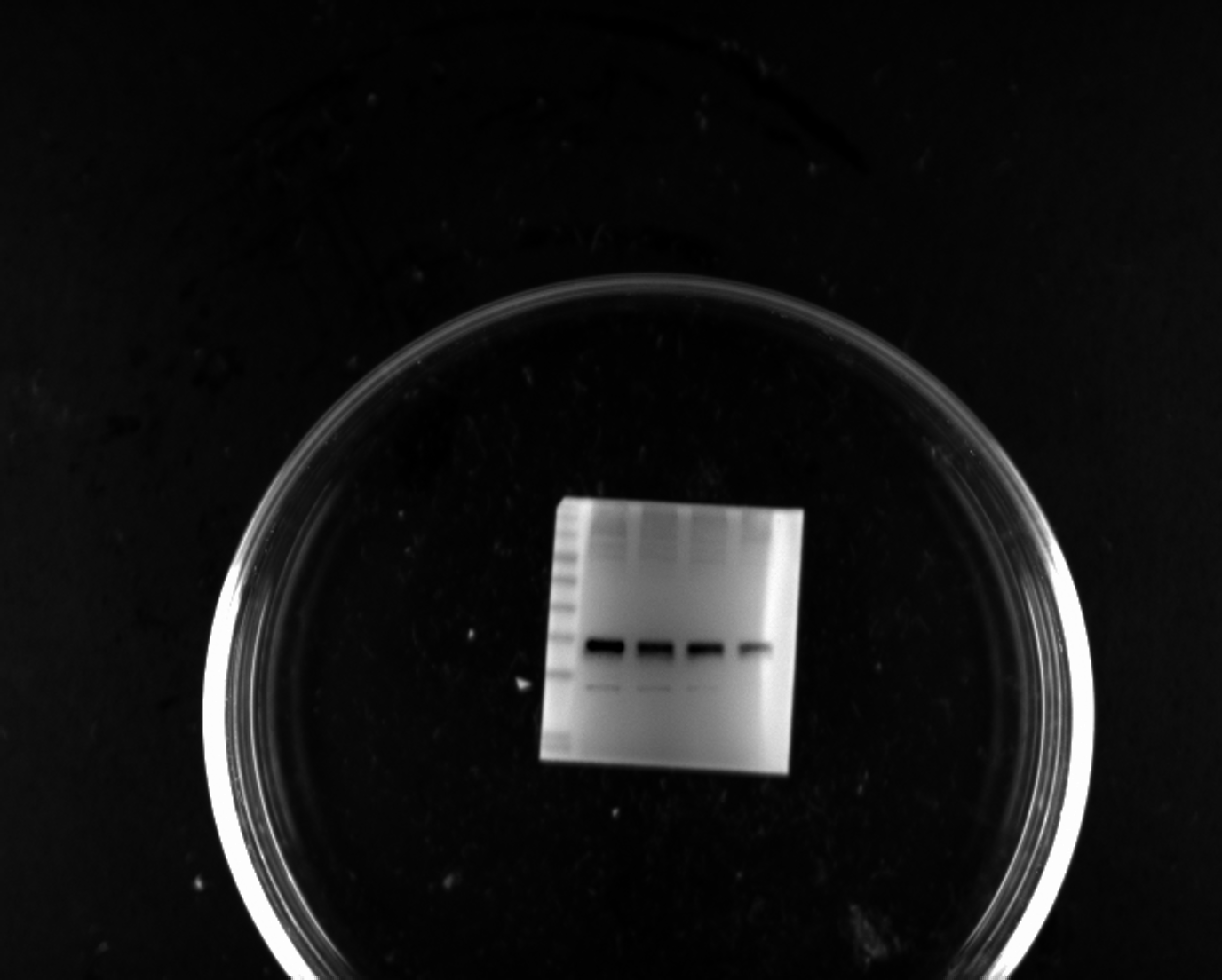


**NCTC1469: β-actin**


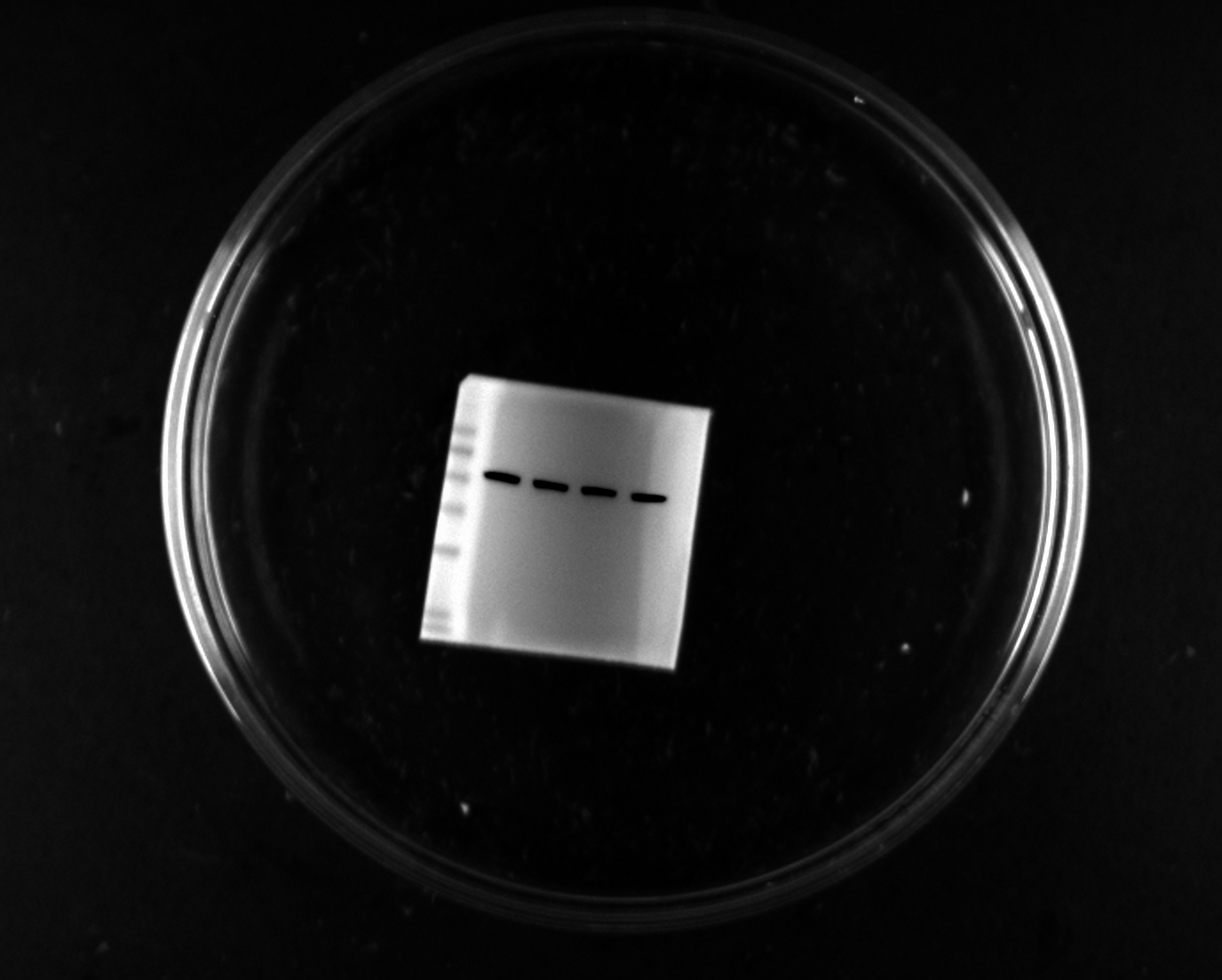


**BNLCL.2: Nuclear Nrf2**


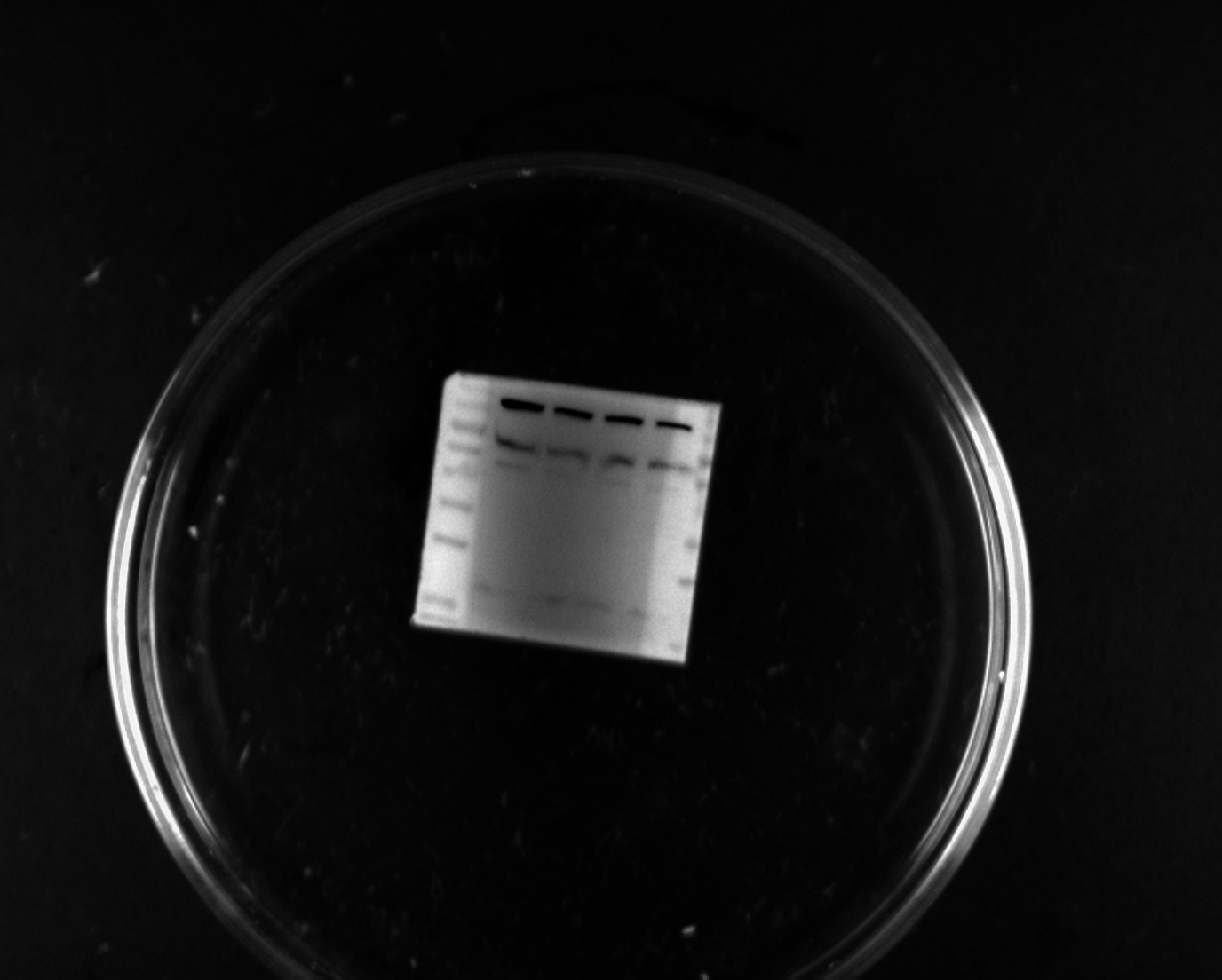


**BNLCL.2: LaminB**


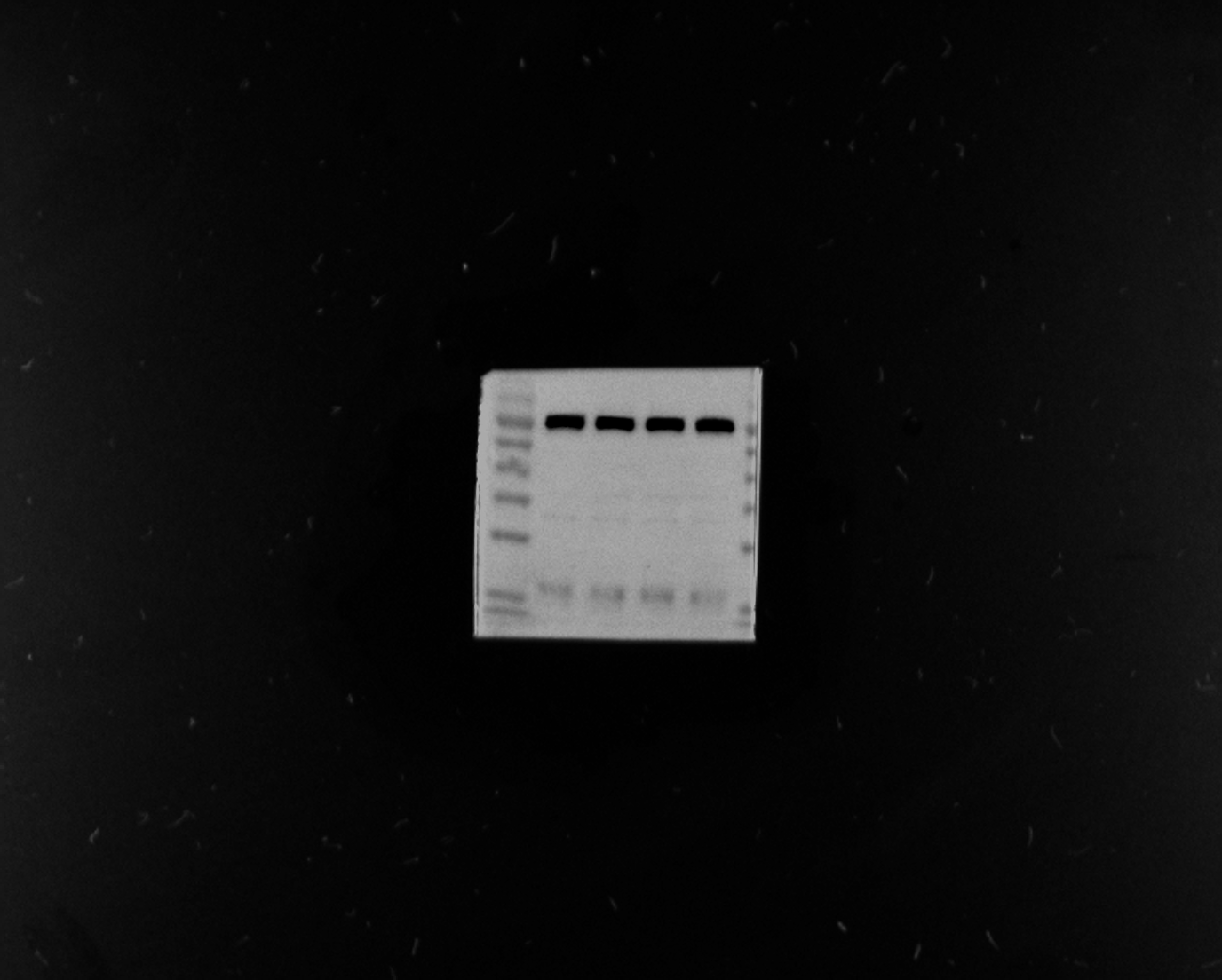


**BNLCL.2: Nrf2**


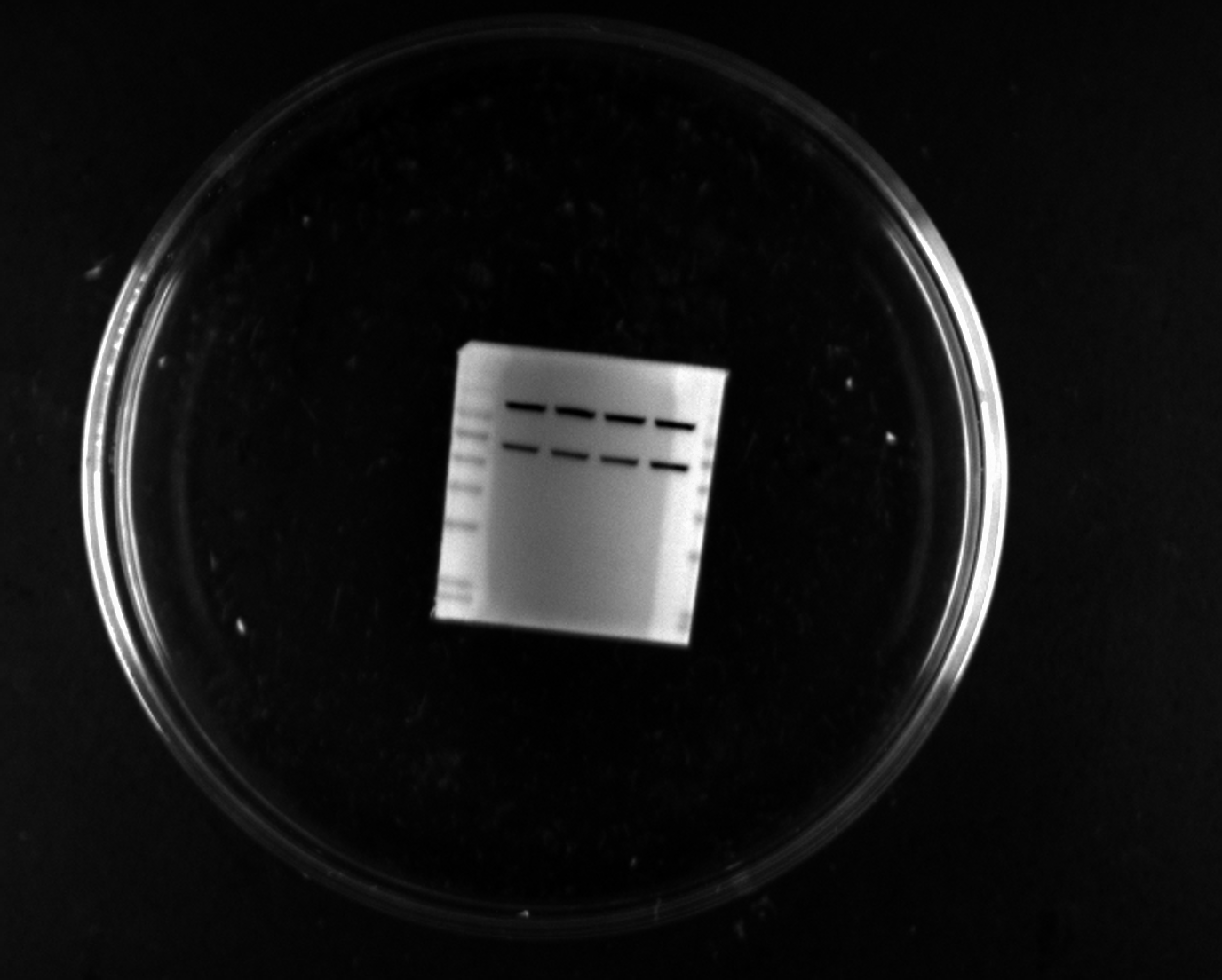


**BNLCL.2: HO-1**


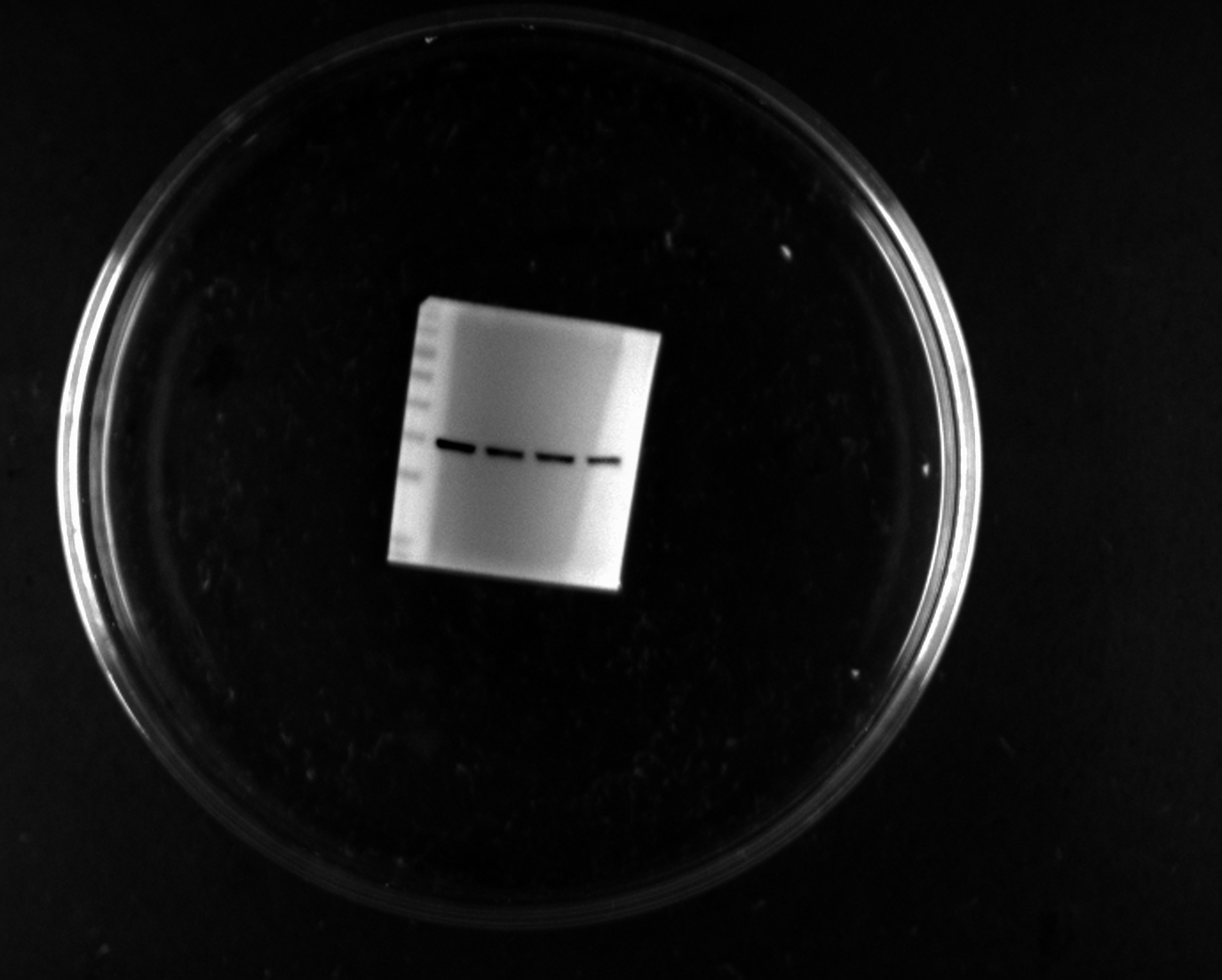


**BNLCL.2: NQO-1**


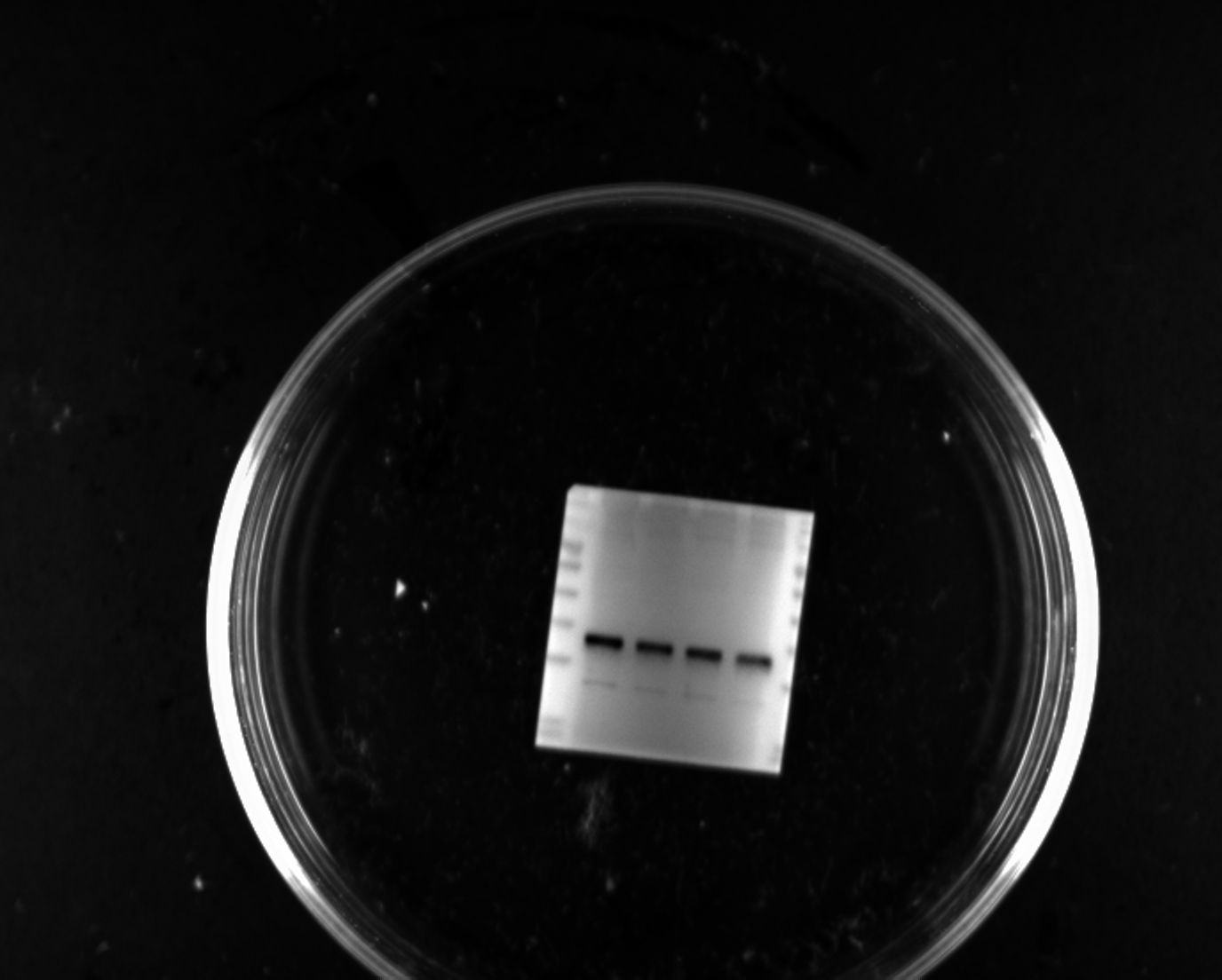


**BNLCL.2: β-actin**


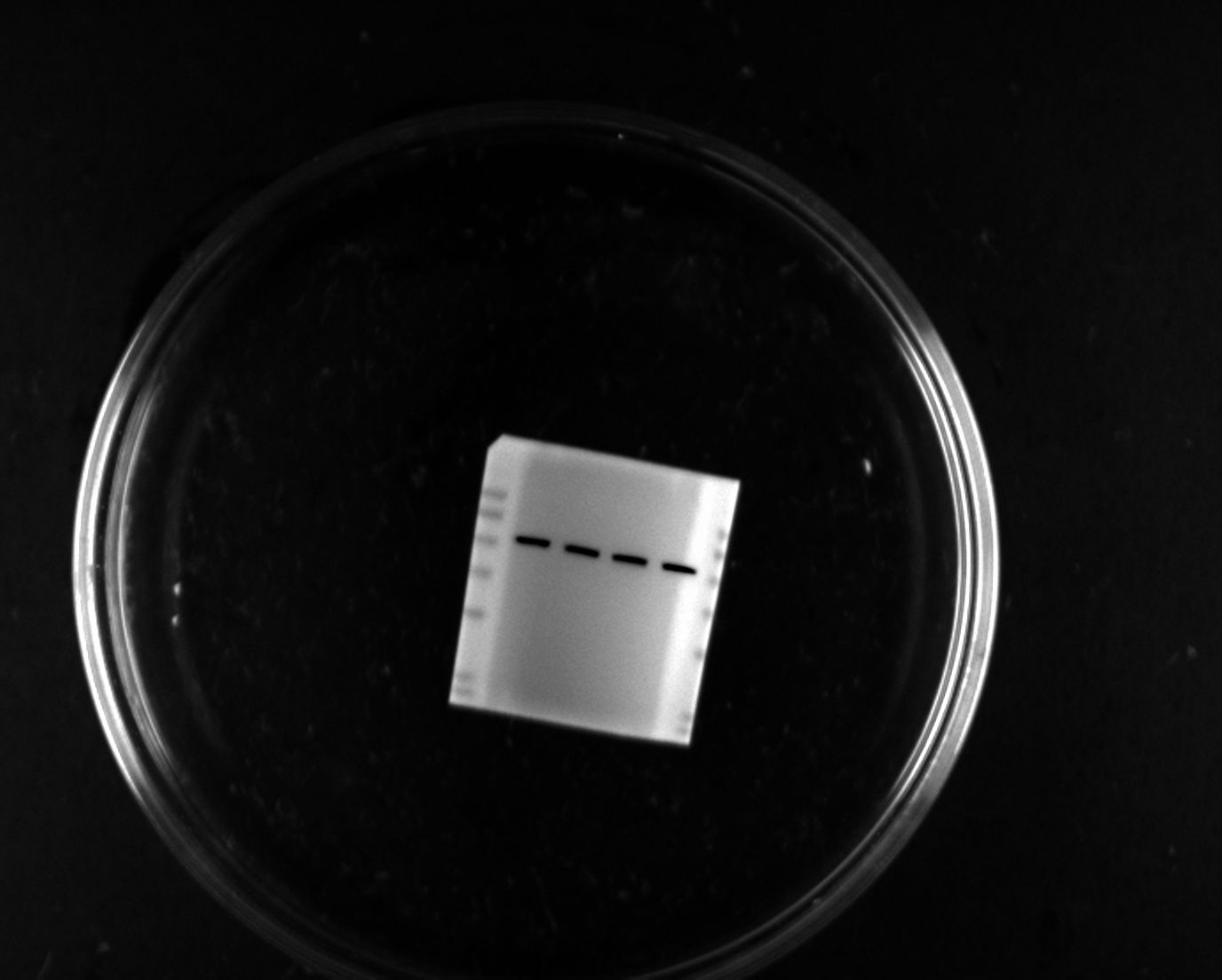


**Figure 6**

**CBX7**


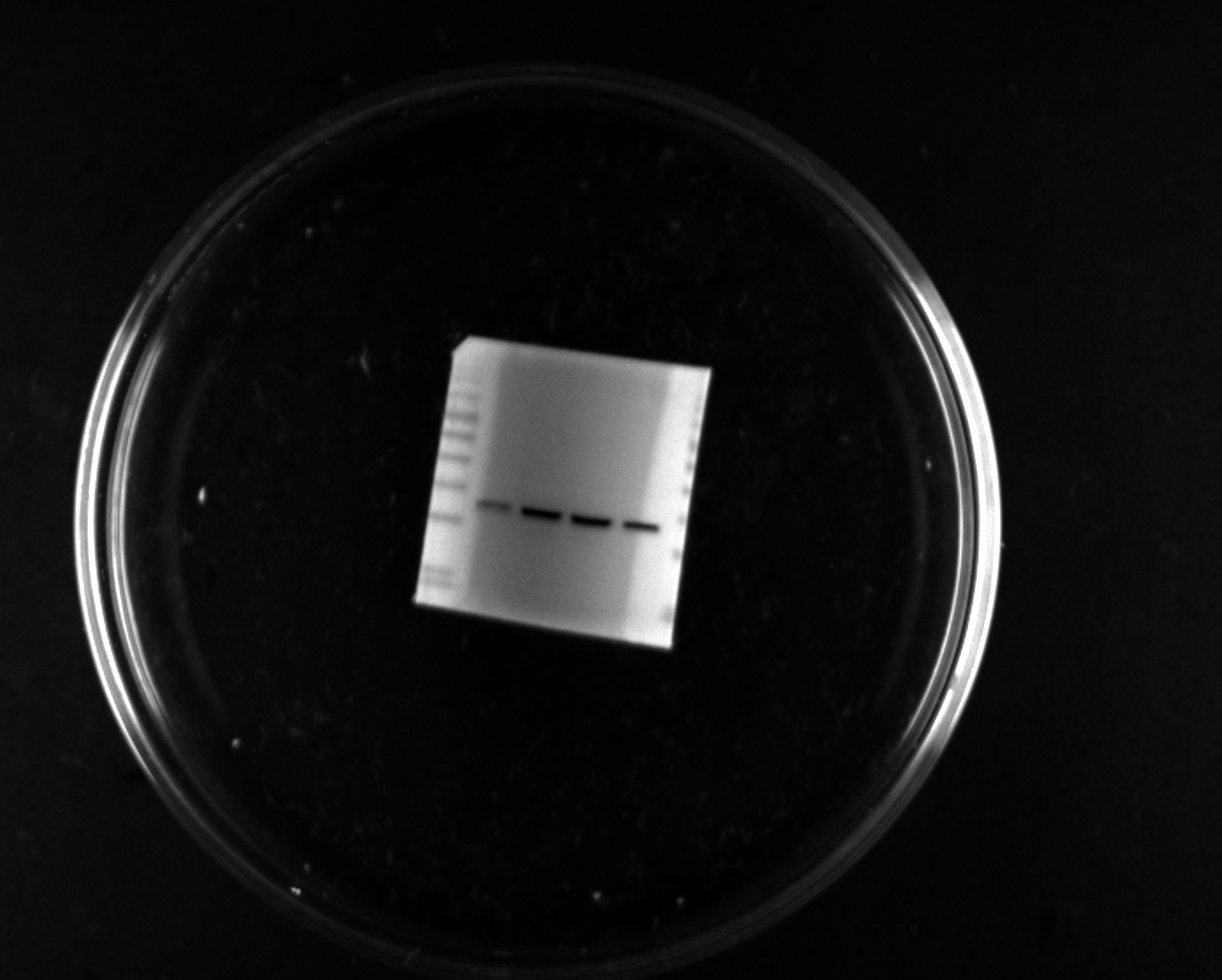


**BMI1**


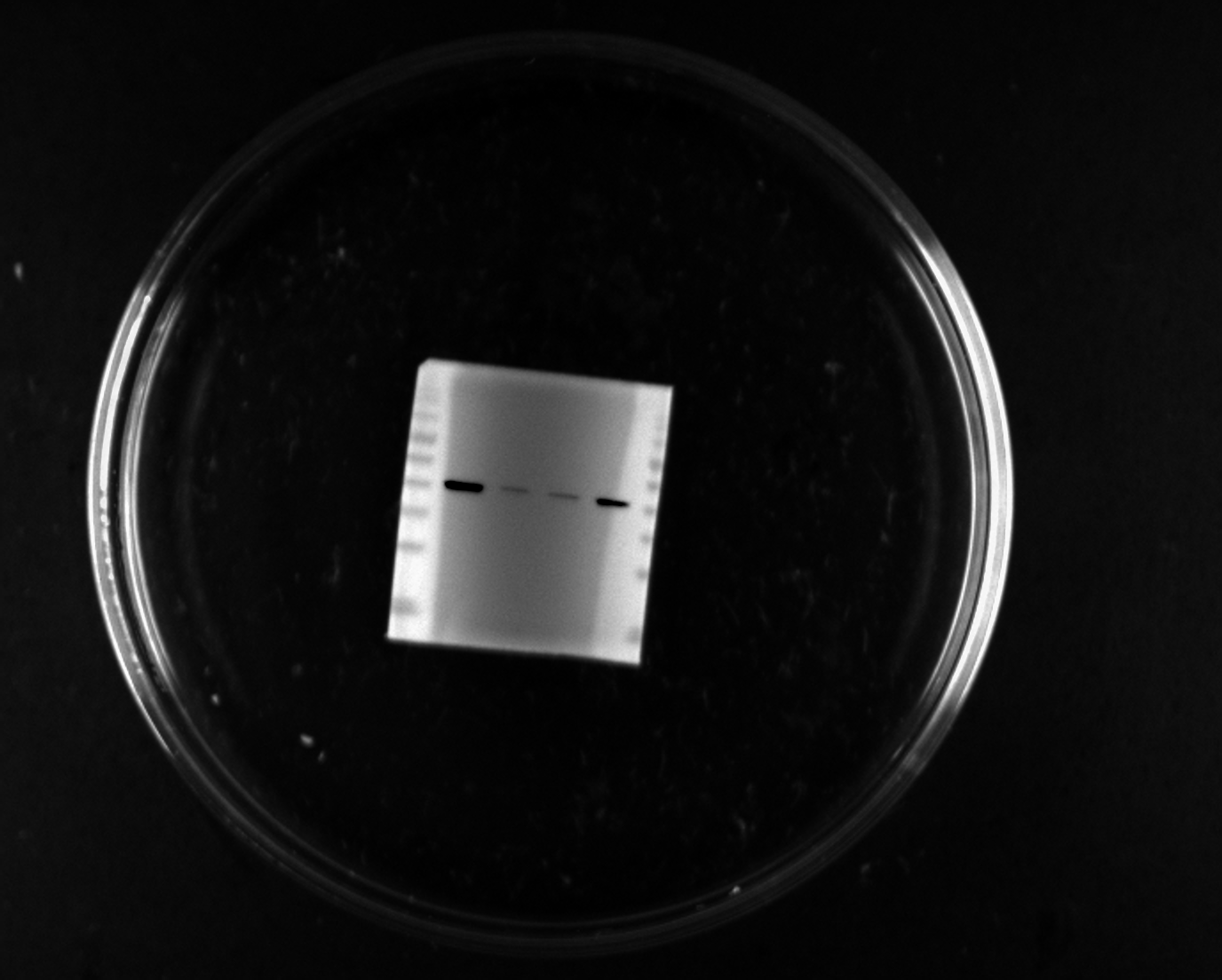


**Nrf2**


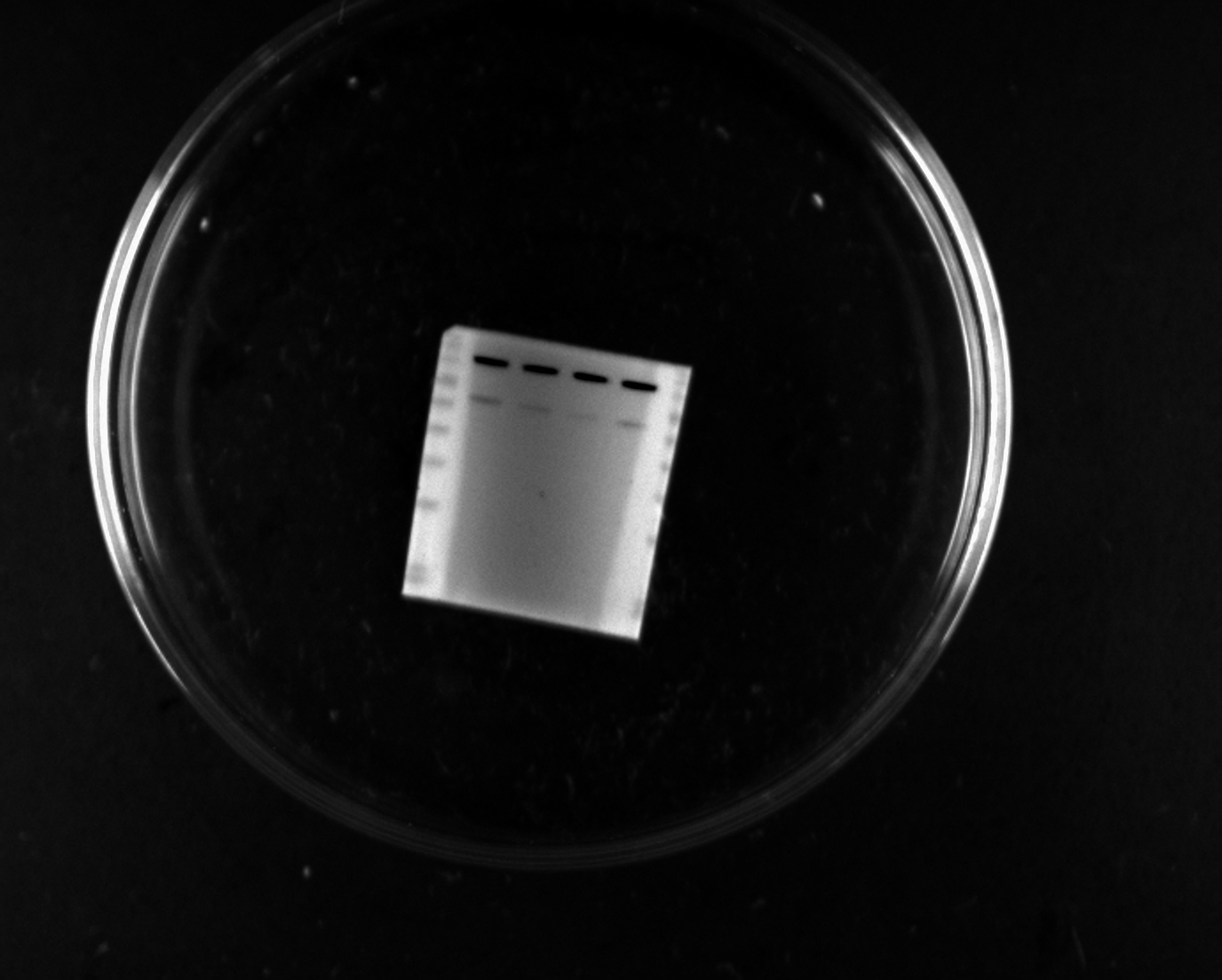


**HO-1**


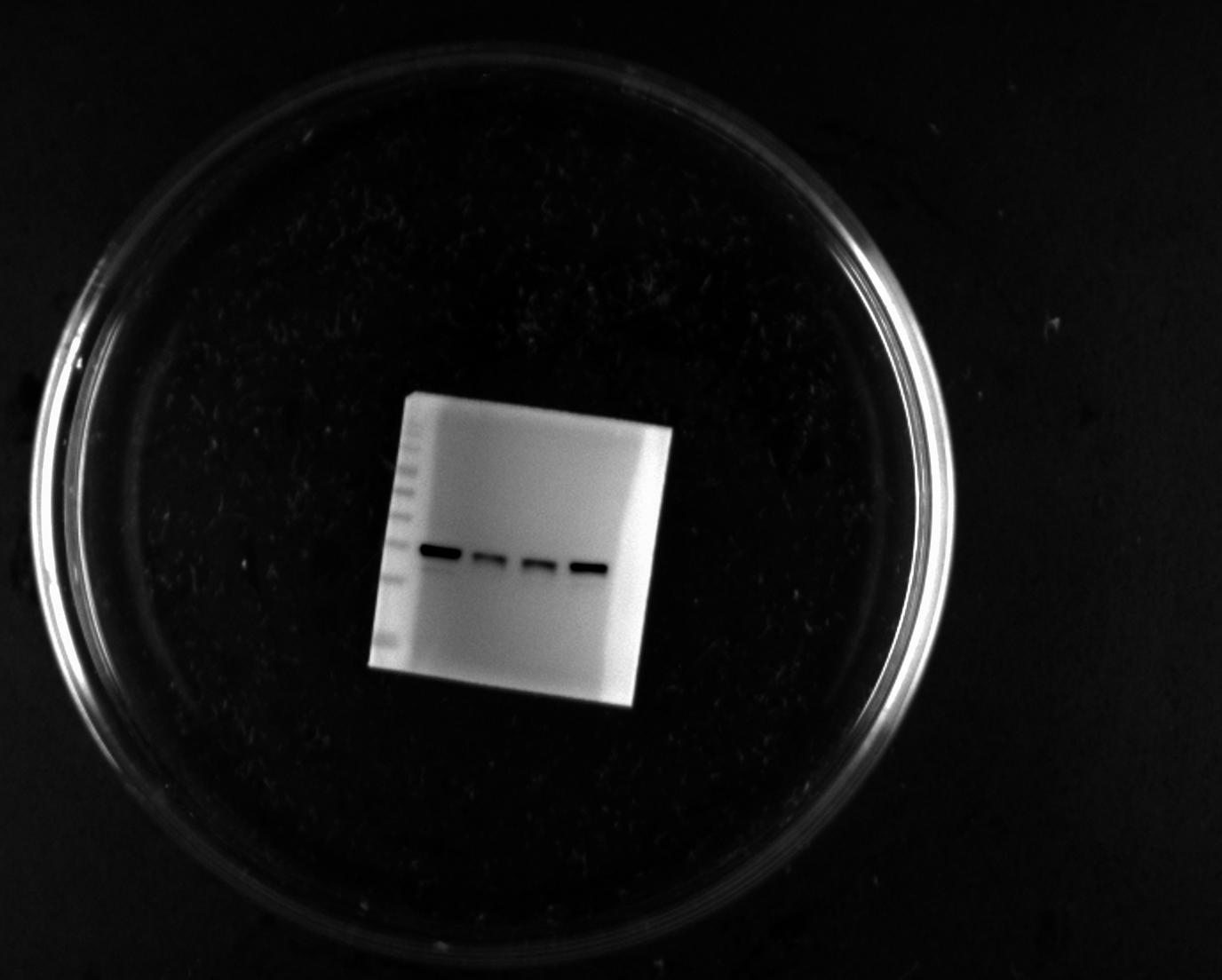


**NQO-1**


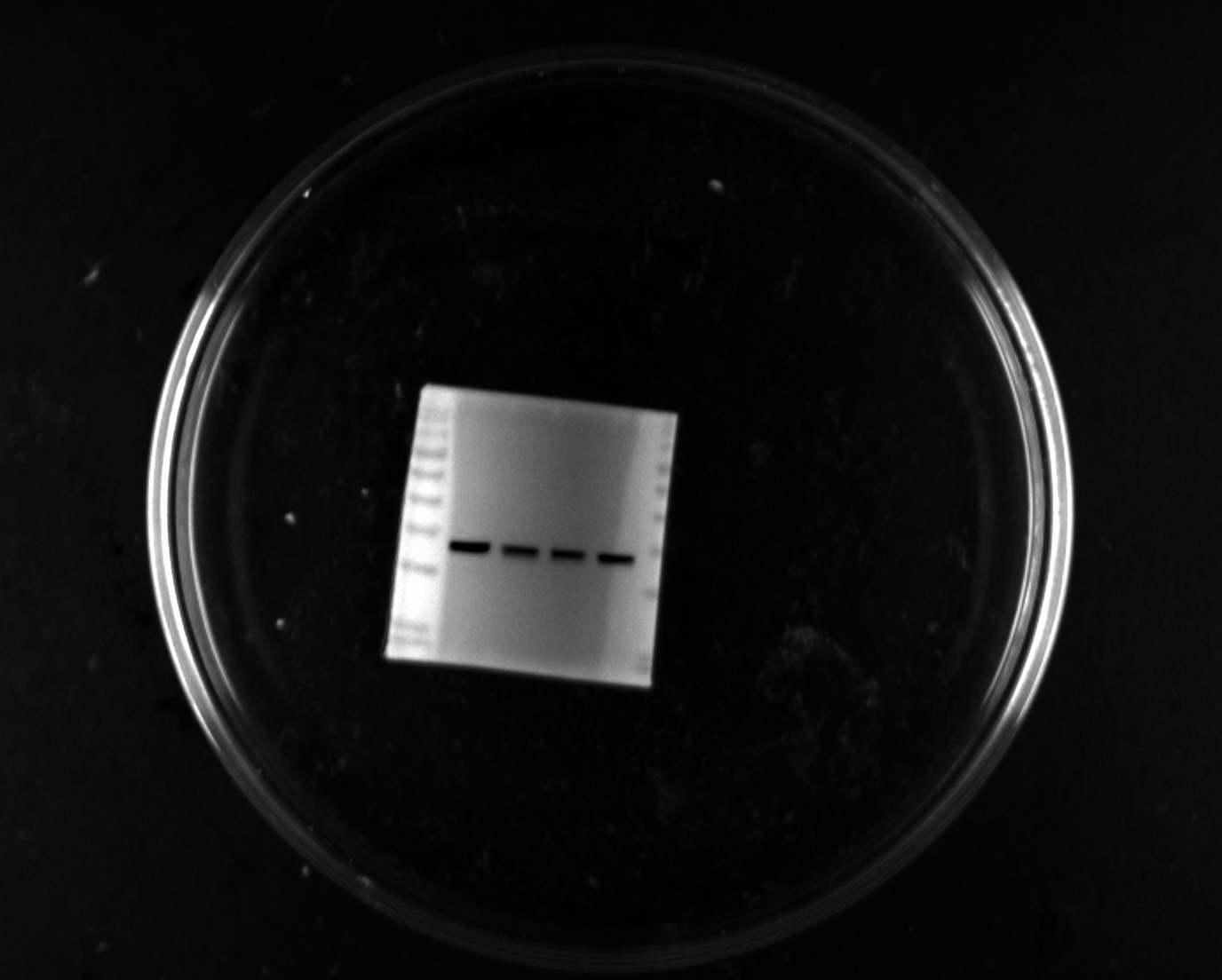


**β-actin**


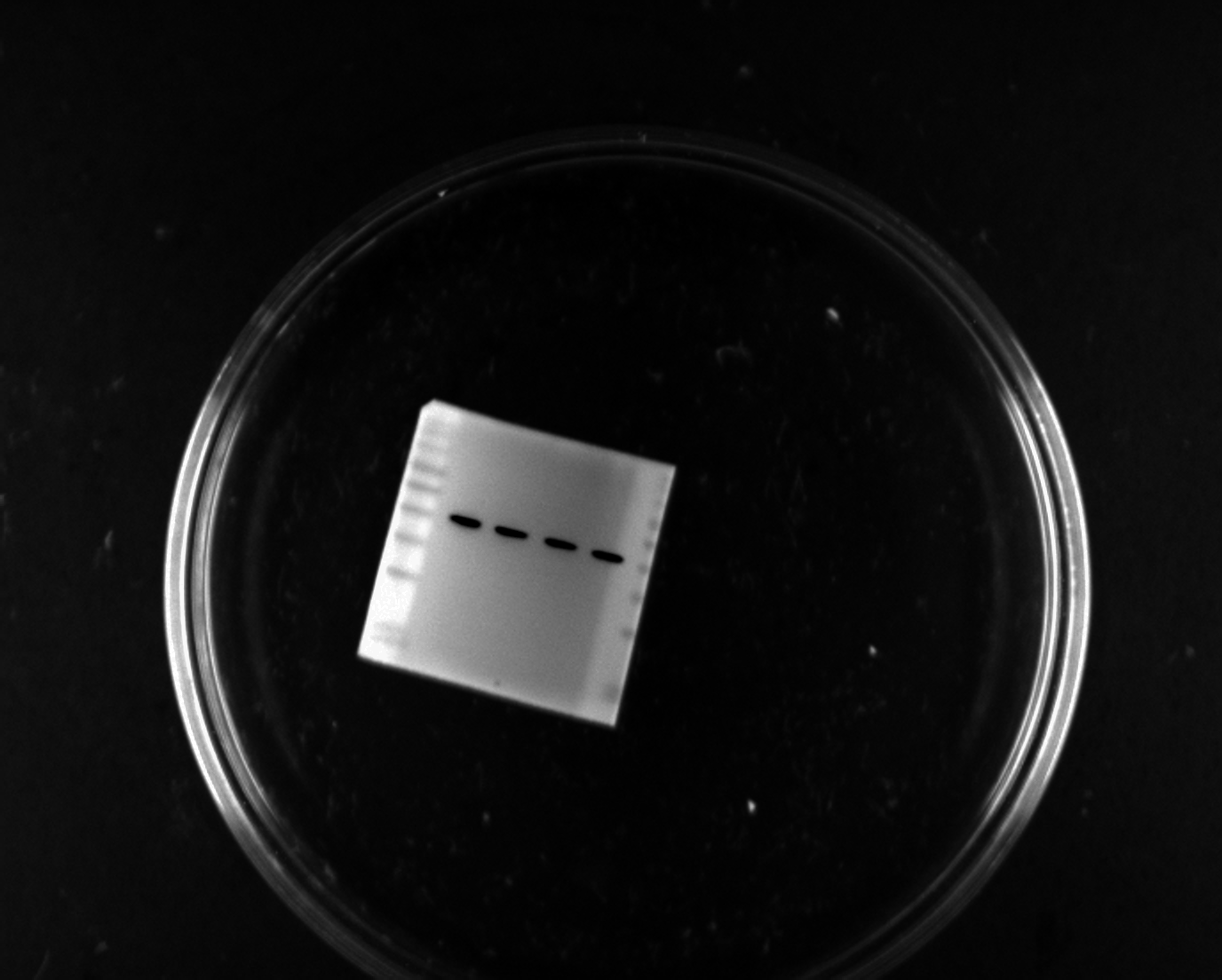


**Nuclear Nrf2**


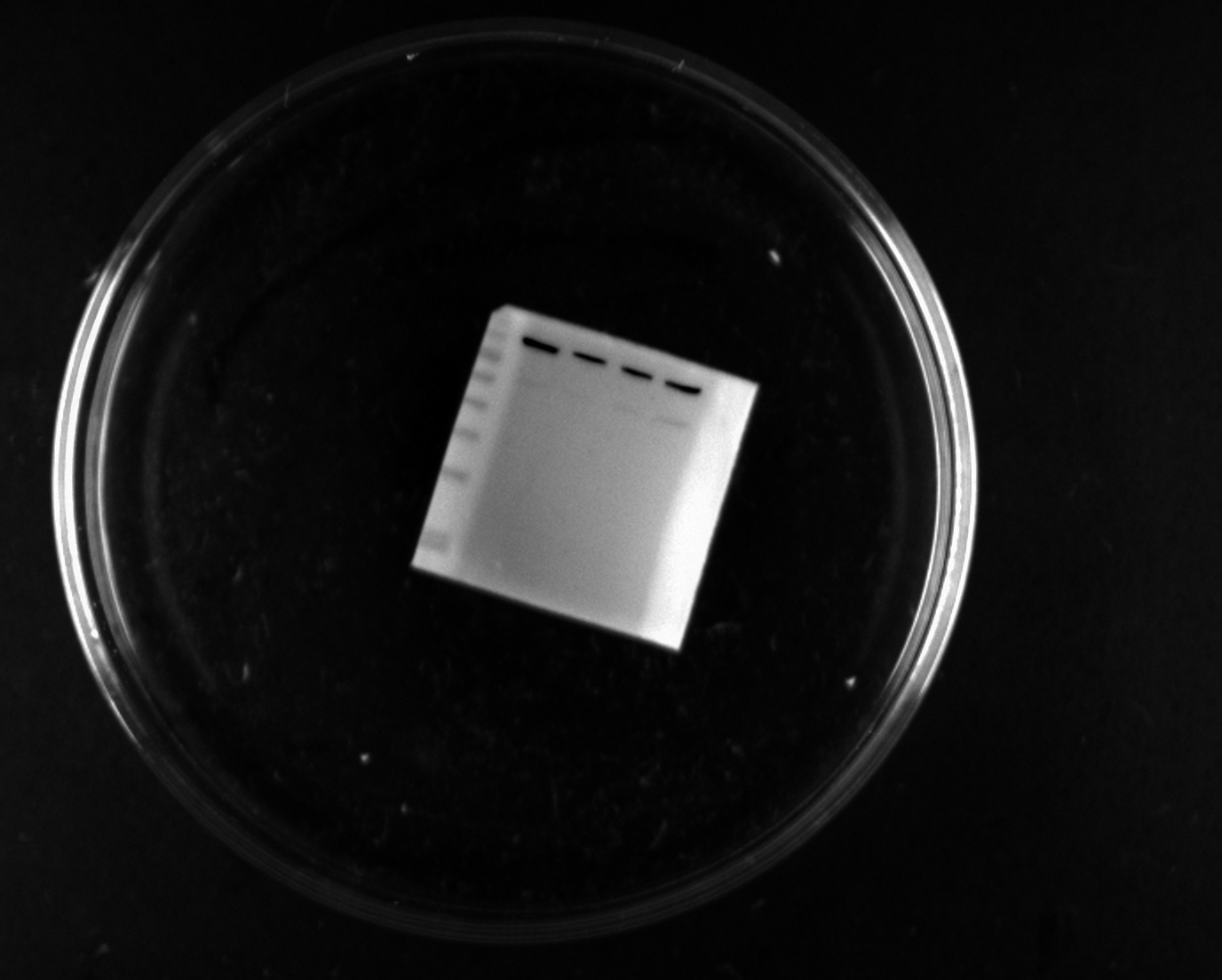


**LaminB**


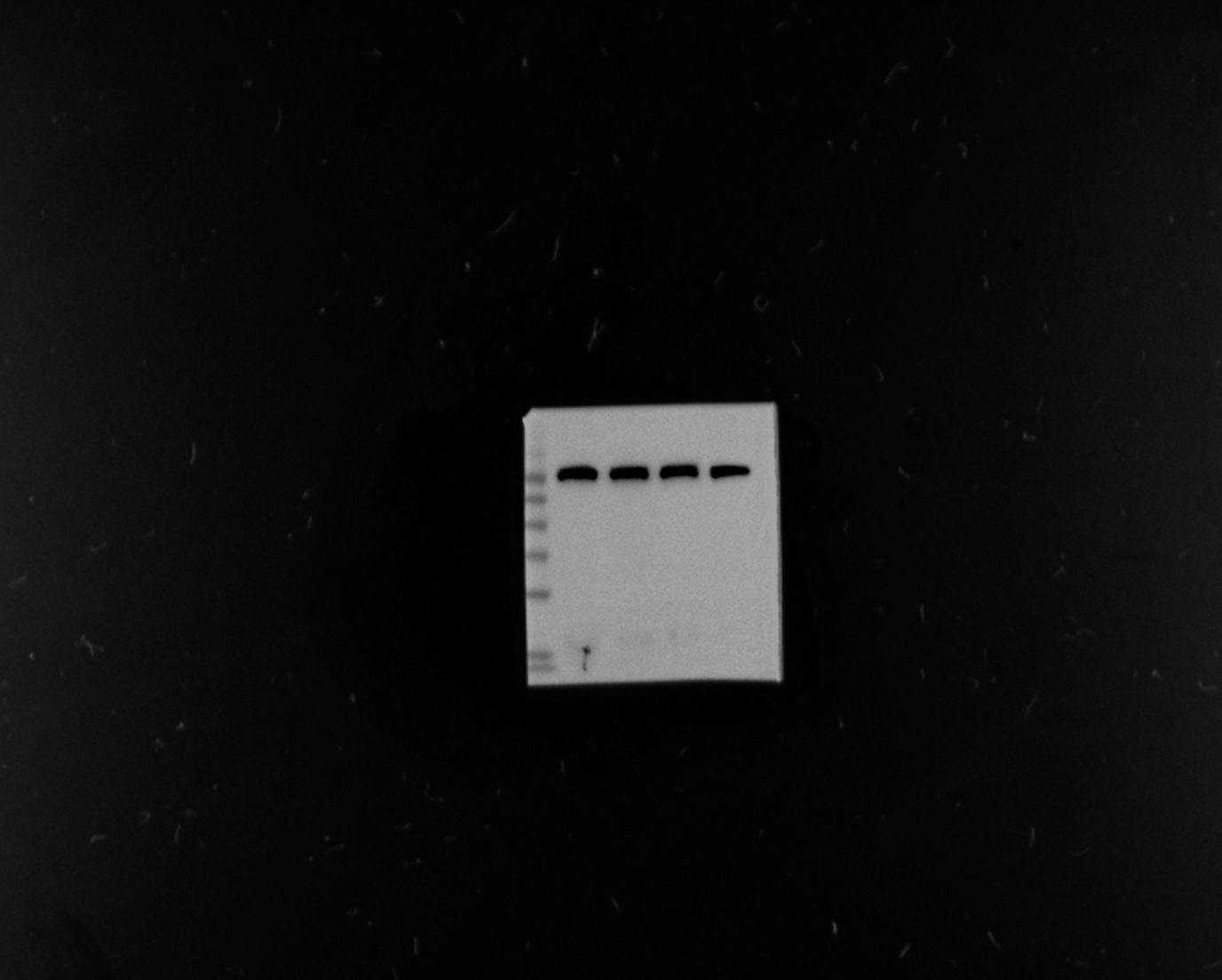

Supplement: Supplementary file 1 — Supplementary Information. [file 41598_2024_58248_MOESM1_ESM.docx]
